# Interpersonal determinants of diet quality and eating behaviors in people aged 13–30 years: A systematic scoping review

**DOI:** 10.1111/obr.13835

**Published:** 2024-09-14

**Authors:** Tanya Braune, Laura Kudlek, Christina Xiao, Hao Tang, Élisabeth Demers‐Potvin, Holly A. Harris, Erin Fitzsimons‐West, Jean Adams, Eleanor M. Winpenny

**Affiliations:** ^1^ MRC Epidemiology Unit University of Cambridge Cambridge UK; ^2^ Centre NUTRISS—Nutrition, Santé et Société, Institut sur la Nutrition et les Aliments Fonctionnels Université Laval Québec Canada; ^3^ Department of Psychology, Education & Child Studies Erasmus University Rotterdam Rotterdam The Netherlands; ^4^ School of Clinical Medicine University of Cambridge Cambridge UK; ^5^ Mohn Centre for Children's Health and Wellbeing, School of Public Health Imperial College London London UK

**Keywords:** diet quality, eating behaviors, interpersonal determinants, young people

## Abstract

Adolescence is an important period of increasing independence, when adolescents experience changing influences of family and friends on their diets as they transition into adulthood. We conducted a scoping review to map the literature on interpersonal determinants of diet quality and eating behaviors among individuals aged 13–30 years. We searched seven literature databases, and following screening, 329 papers were included. Determinants were grouped according to sub‐categories of the Determinants of Nutrition and Eating framework: family structure (*n* = 122), social influences (*n* = 121), parental behaviors (*n* = 90), family food culture (*n* = 83), social support (*n* = 69), parental feeding styles (*n* = 24), parental attitudes/beliefs (*n* = 8), and parental resources/risk factors (*n* = 6), and we added two new sub‐categories: parenting style (*n* = 74) and partner behaviors (*n* = 6). Fruit/vegetable (*n* = 143) and sugar‐sweetened beverage (*n* = 102) intake were the most commonly measured diet outcomes, and breakfast consumption (*n* = 41) and fast food/takeaway intake (*n* = 39) the most commonly examined eating behaviors. This review highlights the gaps in the literature, both across the determinant sub‐categories and also the relative paucity of longitudinal evidence and lack of evidence in emerging adults, particularly outside of university settings. Future research should focus on these areas to provide stronger evidence to support better design of interventions for this age group.

**Abbreviations**
Add Health: National Longitudinal Study of Adolescent Health
ARFS: Australian Recommended Food Score
BMI: body mass index
DASH: Dietary Approached to Stop Hypertension
DGI: Dietary Guidelines Index
DONE: Determinants of Nutrition and Eating
DQI: Diet Quality Index
DTP: Doctoral Training Partnership
ENDORSE: Environmental Determinants of Obesity in Rotterdam School children
FLASHE: Family Life, Activity, Sun, Health and Eating
GSHS: Global School‐Based Student Health Survey
GUTS: Growing Up Today Study
HBSC: Health Behavior in School‐Aged Children
HEI: Healthy Eating Index
HELENA: Healthy Lifestyle in Europe by Nutrition in Adolescence
INQ: Index of Nutritional Quality
MRC: Medical Research Council
PeNSE: National School Health Survey
PRISMA‐ScR: Preferred Reporting Items for Systematic Review and Meta‐analysis Protocols Extension for Scoping Reviews
Project EAT: Project Eating and Activity over Time
SEP: socioeconomic position
SSBs: sugar‐sweetened beverages
UK: United Kingdom
UPFs: ultra‐processed foods
USA: United States of America
WHO: World Health Organization
YEP: Youth Eating Patterns

## 1 INTRODUCTION

The prevalence of people with overweight and obesity is rapidly increasing,^1^, ^2^, ^3^ particularly among adolescents.^4^ With growing evidence linking diet with overweight, obesity, and chronic diseases,^5^ public health recommendations continue to emphasize the value of a healthy diet during adolescence to help reduce the risk of chronic disease development later in life, including cardiovascular disease, type 2 diabetes, and certain cancers.^6^, ^7^, ^8^, ^9^, ^10^, ^11^, ^12^, ^13^ It has been shown that nutrition‐related conditions that develop during adolescence, such as obesity, adiposity, and elevated body mass index (BMI), can continue into adulthood and, through parenthood, affect subsequent generations.^4^, ^14^, ^15^ Despite the known health risks due to unhealthy diets, studies demonstrate that adolescents are not meeting the frequently used guideline recommending “five‐a‐day” fruit and vegetable portions, and their intake of sugar‐sweetened beverages (SSBs) and fast food is high.^16^ To reduce the risk of these adverse health effects, it is therefore of particular importance to better understand what factors influence diets in adolescence that may contribute to the establishment of dietary habits long‐term.

Dietary intakes and associated eating behaviors are complex and can be characterized in a variety of different ways. For instance, a “healthy diet” can be characterized by higher levels of fruit and vegetable consumption, diversity and balance of food groups, or lower intake of fats or sugars.^17^ Additionally, there are established and validated indicators such as the Dietary Approached to Stop Hypertension (DASH) diet,^18^, ^19^ the Healthy Eating Index (HEI),^20^ or adherence to the Mediterranean diet^21^, ^22^ that attempt to quantify the quality of people's diets and their relationship with health outcomes. In addition to these measures, eating behaviors concerning where, when, and how individuals consume food defined by consumption habits and food choices also provide an indication of the healthiness of an individual's eating pattern.^23^

Adolescence is defined by the World Health Organization (WHO) as a life stage between the ages of 10–19 years.^24^ The Lancet proposes an extension of this as the period of adolescent growth encompassing 10–24 years.^25^ This definition also includes the transitional phase of life between adolescence and young adulthood that has been coined as “emerging adulthood,” comprising of individuals aged 18–25.^26^ Emerging adulthood in particular is a period of change during which young people experience a variety of influences on their diets in the transition from adolescence to early adulthood.^27^ One important sphere of influence is the “interpersonal,” referring to the relationships between people, including family, friends, community members, and acquaintances.^28^ Interpersonal factors are of particular importance during adolescence and young adulthood as they are experiencing social reorientation. This psychological shift involves a transition in influence from parents to peers, marked by an increase in time spent with peers,^29^ particularly during early and middle adolescence.^30^ Studies have shown that interpersonal determinants such as parental diet, peer modelling, and shared family meals positively affect children's and adolescents' overall diet quality.^31^, ^32^, ^33^ For example, a 10‐year longitudinal study in adolescents and young adults has demonstrated that shared mealtimes predict healthier dietary intake,^34^ showing the potential importance of how the interpersonal aspects of eating impact diet quality.

As adolescents become older, they gain more independence in relation to their food choices and preferences, becoming less influenced by their parents or caregivers and more influenced by their peers.^35^ Their eating behaviors are often characterized by an increase in fast food intake, eating outside of the home, and meal skipping.^28^ A pooled qualitative study and literature review investigating influences on adolescents' food choices demonstrated that socializing with friends around food took precedence over other factors such as taste or healthiness and gave adolescents an increasing sense of belonging, identity, and autonomy.^16^ However, there has not been a systematic synthesis and mapping of the broad scope of evidence on interpersonal influences on diet in this particular age group. Previously published reviews have provided a much broader perspective on all determinant levels (individual, environmental, policy, and interpersonal)^27^ and do not capture specific details within the interpersonal level, or have narrowed their focus down to one determinant category, for example, social norms or parental influences.^36^

This review included individuals aged at least 13 years, as the focus is on mid‐adolescence and the emerging adult period, and no older than 30 years. The age range extends up to 30 years to ensure that papers studying the interpersonal influences during early adulthood are captured, reducing the likelihood of narrowing the search to solely include family influences. Because there is a clear need to improve diets in adolescents and young adults, studying and understanding how diet quality and eating behavior patterns are established through interpersonal influences from their social environment could allow for better targeted interventions and recommendations for this age group.^31^

A scoping review is a useful method to categorize and map existing literature, gauge the breadth of research, provide a summary of its thematic focus, and identify preliminary questions for systematic reviews.^37^ Despite the large evidence base on interpersonal determinants of diet quality and eating behaviors in young people, no other reviews have synthesized the full extent of current knowledge around interpersonal determinants or specifically focussed on this developmental life stage. Therefore, our aims in this scoping review were to:

1. Summarize the extent of the current research evidence on interpersonal determinants of diet and eating behaviors in young people aged 13–30 years.
2. Map the evidence against an existing conceptual framework of interpersonal determinants to identify gaps in the evidence base.
3. Identify opportunities for future research to enhance our understanding of potential causal mechanisms of healthy/unhealthy diets and eating behaviors in this population.

## 2 METHODS

A systematic scoping review method was followed according to published guidelines.^38^, ^39^, ^40^ The protocol was written according to the Preferred Reporting Items for Systematic Review and Meta‐analysis Protocols Extension for Scoping Reviews (PRISMA‐ScR)^41^ and registered with International Prospective Register of Systematic Reviews (PROSPERO) on March 16, 2022 (CRD42022316031). The PRISMA‐ScR checklist can be found in Table S1.

### 2.1 Conceptual framework for evidence mapping

Conceptual frameworks have been important tools in gaining a better understanding of the complex influences on diet and eating behaviors experienced by young people. In order to map the available evidence on interpersonal determinants, five conceptual frameworks were considered for this review. These frameworks were: (1) Commission on Social Determinants of Health Conceptual Framework,^42^ (2) The “Innocenti” Framework,^43^ (3) The Lancet Series on Adolescent Nutrition Conceptual Framework for Adolescent Growth and Nutrition,^44^ (4) A Conceptual Framework for Understanding Adolescent Eating Behaviors,^28^ and (5) The Determinants of Nutrition and Eating (DONE) Framework.^45^, ^46^ The only framework that considered both the breadth of interpersonal influence across family and peer influence domains and included sufficient detail on different aspects of interpersonal factors was the DONE Framework. Therefore, we chose to use this as the framework against which to map the literature. As defined by the DONE framework, the interpersonal level contains a social and cultural category, and in this review we focussed on the social category. The social category contains sub‐categories, within which examples of determinants are provided. Table 1 outlines these examples of determinants under the nine sub‐categories of the social category at the interpersonal level. Where possible, determinants were classified by the primary reviewer (TB) into these existing sub‐categories, and additional sub‐categories were added, as required, to capture the breadth of available evidence.

**TABLE 1:** The sub‐categories of the social category in the interpersonal level of the Determinants of Nutrition and Eating (DONE) framework, with examples of determinants.^46^

Sub‐category	Explanation	Examples of determinants
Family structure	“Composition and cohesion of the family/household”	Caregiving role, family cohesion, family composition, household size
Family food culture	“Food culture existing in the family/household”	Family food culture, family preferences, household food processing (cooking), household food processing (growing)
Household socioeconomic status	“Socio‐economic aspects of the family/household”	Household budget constraints, household food security, household income, household socio‐economic status, low parental income, parental education level, parental food insecurity, parental income, parental occupation
Social influences	“Diet‐ and eating‐related influences from others in the environment”	Inter‐generational influences on diet, decisional power in groups, eating occasion, peer modelling, perceived important other's dieting, presence of others, social facilitation of dieting, social norms, social relationships
Social support	“Diet‐ and eating‐related support from others in the environment”	Bonding with parents, community recommendations, frequency of social contacts, parental recommendations, peer cooking practices and skills, salesperson recommendations, social support, social ties
Parental resources and risk factors	“Parental resources and constraints relevant for diet and eating”	Parental depression, parental food market knowledge, parental food product knowledge, parental nutrition knowledge, parental time constraints
Parental attitudes and beliefs	“Parental thoughts and beliefs about food and eating”	Parental body dissatisfaction, parental food ethics, parental risk aversion, parental lay food theories, parental perceived food safety, parental perception of child's weight, parental trust in food certification, parental trust in food distribution, parental trust in food labelling, parental trust in food producers, parental weight control concerns, parental weight control goal, parental willingness‐to‐pay
Parental behaviors	“Parental food‐ and eating‐related behaviors”	Parental conformity to tradition, parental food habits, parental food processing (cooking), parental food production (growing), parental frugality, parental lifestyle, parental modelling, parental smart shopping
Parental feeding styles	“How parents go about feeding their children”	Food used as an incentive, parental complementary feeding practices, parental emotional feeding, parental food restriction, parental instrumental feeding, parental portion size habits, parental pressure to eat

### 2.2 Information sources and search strategy

Seven electronic databases were searched: Medline, Embase, PsychINFO, Web of Science, SCOPUS, ASSIA, and Global Health. Relevant reviews were identified during screening. Although these were not included, their reference lists were examined for additional eligible papers. Thirty texts were added for full text screening using this method.

Search strategies were formatted for each database and checked with a medical librarian at the University of Cambridge. Articles were not restricted by country or date, and the search results could include articles in any language. Searches were initially conducted on February 28, 2022 and repeated on March 27, 2023. Terms relating to interpersonal determinants (e.g., [interpersonal], [“social determinant*”]), diet quality (e.g., [diet*], [“healthy eating habit*”]) or eating behaviors (e.g., [“eating behavio*”], [“fast food”]), adolescence and young adulthood (e.g., [adolescen*], [“young adult”]), and observational methodology (e.g., [“observational”]) were combined during the search. See Table S2 for the detailed search strategy used in Medline.

### 2.3 Screening

Search results were imported to and automatically de‐duplicated in Covidence (Covidence Systematic Review Software, Veritas Health Innovation, Melbourne, Australia. Available at www.covidence.org). An additional 142 duplicate references were manually removed during screening. All screening was conducted through Covidence, and Mendeley Reference Manager software was used to manage all references.

A random sub‐sample of 300 papers was pilot screened by five reviewers (TB, LK, HAH, EFW, and EMW) according to the eligibility criteria and assessed for agreement. After the pilot screening, the inclusion and exclusion criteria were refined. Title/abstract screening was conducted by the primary reviewer (TB) or one of four secondary reviewers (LK, HAH, EFW, and EMW). Once all titles and abstracts were screened, the eligibility criteria were further refined, notably to exclude interpersonal determinants relating to household socioeconomic position (SEP). This was due to the high volume of included texts and the research team's decision that SEP fell outside of the scope of this review, as this related more to resources and structural or circumstantial factors as opposed to interpersonal influences. Therefore, any papers that investigated socioeconomic measures (e.g., parental education, parental income, and parental occupation) as their only exposure variable were excluded. Table 2 outlines the final eligibility criteria used.

**TABLE 2:** Inclusion and exclusion criteria.

	Inclusion criteria	Exclusion criteria
Setting	Any country, any language, human studies.	
Population	Adolescents and young adults: Mean age of participants should fall within 13–30 years old. If no mean age reported, middle of age range should fall within 13–30 years old.	Any other age group. Institutionalized or clinical populations. Pregnant and lactating women only.
Exposures	Interpersonal determinants related to social aspects (i.e., family, parental, peer, and partner). These do not have to be related to food/diet. Examples include but are not limited to DONE framework examples (see Table 1). Social media use is only considered eligible if measured in terms of peer behaviors on social media, rather than exposure to marketing/celebrities/influencers.	Papers only assessing structural factors such as food systems, the physical food environment, or SEP.
Outcomes	Diet quality score/index or dietary pattern/diversity, including scores developed by authors. Food groups or macronutrients used as an indicator of diet quality, for example, fruit and vegetables, sugar‐sweetened beverages, ultra‐processed foods, total energy intake, and fat intake. Eating behaviors relating to eating outside of the home, fast food consumption or takeaways, meal/breakfast skipping, or snacking behaviors.	Alcohol/smoking/substance use only Eating disorders (anorexia, bulimia, etc.), including emotional/binge eating. Weight loss behaviors including drive for thinness or dieting to lose weight. Measures of malnutrition or nutritional status, for example, individual biomarkers, stunting, wasting, and BMI. Food insecurity, food insufficiency, or undernutrition. Nutrition knowledge and intentions (rather than intake).
Study types	Qualitative and quantitative observational studies.	Any other study designs (interventions, trials, case–control studies, reviews).
Publication type	Journal articles to include only peer reviewed research.	Any other type of publication (e.g., conference abstracts, study protocols, reports, dissertations, or books).

Abbreviations: BMI, body mass index; DONE, Determinants of Nutrition and Eating; SEP, socioeconomic position.

Full texts were retrieved for all but 10 papers after title/abstract screening. The retrieved papers were assessed based on the same eligibility criteria outlined in Table 2. Full text screening was conducted by the primary reviewer (TB) independently in duplicate with one of three secondary reviewers (LK, CX, HT). More than 80% inter‐rater agreement was achieved prior to further discussions. All conflicts were resolved through one‐on‐one discussions between the primary reviewer and each secondary reviewer.

### 2.4 Data charting

Pilot data charting was carried out in duplicate by working independently using a form to collect data from 10% of included papers by the primary reviewer (TB) and one secondary reviewer (EDP). For this sub‐sample, inconsistencies in pilot data charting were due to differences in wording rather than content; therefore, all remaining data extraction was conducted by the primary reviewer only. The following data items were collected for the included papers:

- General information: Title, author, country
- Study characteristics: Year of data collection, dataset used or location of data collection (school/university/city), aim, study design, sample size, mean age/middle of age range, and standard deviation
- Exposures: Interpersonal determinant(s) studied, DONE framework categorization (if applicable)
- Outcomes: Diet quality measure(s)/eating behavior(s) assessed
- If qualitative: Key themes and outcome discussed

The “middle age” of all participants studied in the included papers was derived from rounding the mean age reported, or if a paper only reported a range, the middle of the range was taken. Ten papers did not specify an age range or mean; however, these referred to their populations as adolescents/teens or high school/first year college/undergraduate/university students and therefore were still included as these were considered to fall within the inclusion criteria.

During data charting, interpersonal determinants studied were classified using the DONE framework sub‐categories, where possible. Grouping was conducted subjectively by the primary author (TB), with discussion with two co‐authors (JA and EMW). This helped identify new determinants and sub‐categories outside of the framework. All data extraction was managed through Covidence.

### 2.5 Synthesis of results

A narrative synthesis of findings from the included papers was carried out using the United Kingdom's (UK) Economic and Social Research Council's guidance.^47^ The results were displayed in bar charts and tables to summarize the context of the research conducted in the included papers, the interpersonal determinants covered by the literature, how they were categorized within the DONE framework, and the diet quality measures and eating behaviors explored.

## 3 RESULTS

### 3.1 Selection of sources of evidence

**FIGURE 1:**
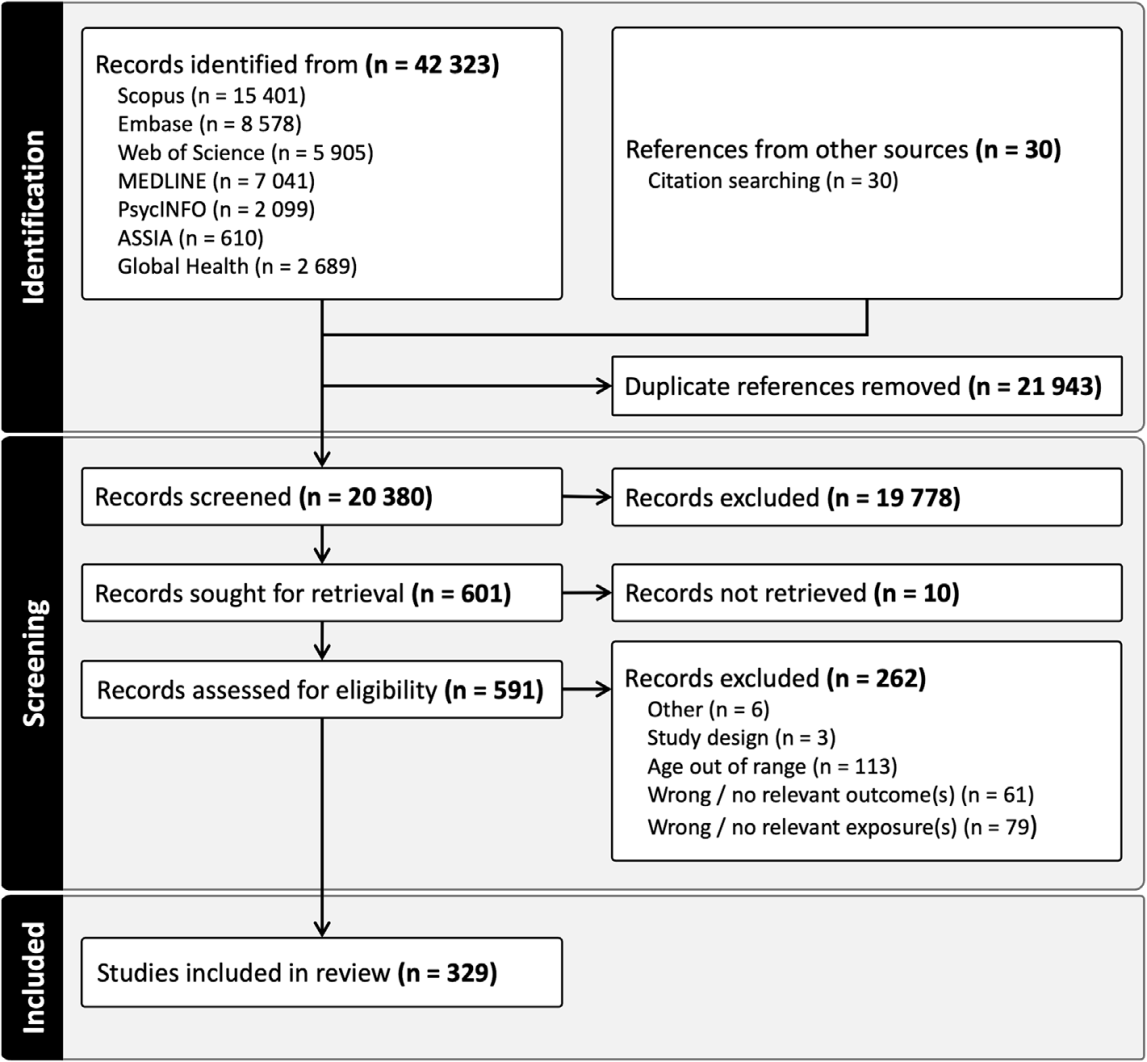
Preferred Reporting Items for Systematic Reviews and Meta‐analyses (PRISMA) flow diagram.

Title and abstract screening was conducted in 20,380 papers, and 601 papers were retained for full text review, of which 329 papers were included in the synthesis. A summary and references of these papers can be found in Table S3. The full table of data extracted for all included papers can be found at the Open Science Framework link: https://osf.io/fx7qe. See Figure 1 for the details of the results of the literature search.

### 3.2 Description of included papers

Of the included papers, the majority (*n* = 265, >80%) used a cross‐sectional design, with less than 15% longitudinal papers (*n* = 45), 5% qualitative papers (*n* = 16), and <1% mixed methods papers (*n* = 3). Data from a total of 91 countries were studied. Table 3 shows the countries in which participants were recruited in the papers identified, noting that some papers recruited participants from more than one country. Almost a third of papers recruited participants from the United States of America (USA), and high‐income countries were highly represented in this topic in comparison to low‐ or middle‐income countries, based on the World Bank classification.^48^ The full table of countries can be found in Table S4.

**TABLE 3:** Number of papers by country with ≥10 published papers.

Country	*n*	%
USA*	105	31.9
Australia*	27	8.2
Brazil	15	4.6
The Netherlands*	15	4.6
The United Kingdom (UK)*	15	4.6
Belgium*	14	4.3
Iran	13	4.0
Germany*	12	3.6
Italy*	12	3.6
Norway*	12	3.6
Spain*	12	3.6
Japan*	12	3.6
Canada*	10	3.0
Sweden*	10	3.0

* High income countries based on World Bank classification.^48^

The settings within which participants were recruited included schools (*n* = 181), cities or regions (*n* = 59), universities (*n* = 57), online (*n* = 15), households (*n* = 7), birth cohorts (*n* = 7), and clinical (*n* = 3). For papers studying participants aged 20 years and older (*n* = 69), 65% of recruitment took place in a university setting (*n* = 45). For those focussing on participants aged 19 years and younger (*n* = 250), 68% of recruitment took place in a school setting (*n* = 169).

Many included papers used specific datasets for analyses. Table 4 demonstrates which dataset was used in which countries with at least three papers published. One in three papers from the USA were conducted using the Project Eating and Activity over Time (Project EAT) dataset, a longitudinal study collecting information on adolescents and young adults since 1997 in Minnesota, USA.^49^ Other datasets with a higher number of papers, such as Health Behavior in School‐Aged Children (HBSC), Healthy Lifestyle in Europe by Nutrition in Adolescence (HELENA), and Global School‐Based Student Health Survey (GSHS), are representative of a wider range of countries.

**TABLE 4:** Number of papers by dataset with at least three published papers.

Name of dataset	Country	*n*	%
Project EAT	USA	32	9.7
HBSC	Multiple*	12	3.6
Family Life, Activity, Sun, Health and Eating (FLASHE)	USA	12	3.6
National School Health Survey (PeNSE)	Brazil	9	2.7
Youth Eating Patterns (YEP)	Australia	5	1.5
HELENA	Austria, Belgium, France, Germany, Greece, Hungary, Italy, Spain, Sweden	4	1.2
GSHS	Indonesia, Iran, Laos, Malawi, Egypt, Jordan, Maldives, Dominica, Grenada, Jamaica, Fiji, Malaysia, Mongolia, Indonesia, Philippines, Thailand	5	1.5
Environmental Determinants of Obesity in Rotterdam School children (ENDORSE)	The Netherlands	3	0.9
National Longitudinal Study of Adolescent Health (Add Health)	USA	3	0.9
Growing Up Today Study (GUTS)	USA	3	0.9

Abbreviations: GSHS, Global School‐Based Student Health Survey; HBSC, Health Behavior in School‐Aged Children; HELENA, Healthy Lifestyle in Europe by Nutrition in Adolescence; Project EAT, Project Eating and Activity over Time.

* Albania, Austria, Belgium, Bulgaria, Canada, Croatia, Czech Republic, Denmark, Estonia, France, Finland, Germany, Greece, Greenland, Hungary, Iceland, Ireland, Israel, Italy, Latvia, Lithuania, Luxembourg, Malta, Moldova, The Netherlands, North Macedonia, Norway, Poland, Portugal, Romania, Russian Federation, Slovakia, Slovenia, Spain, Sweden, Switzerland, UK, Ukraine, USA.

#### 3.2.1 Participants

Over 60% of papers were in participants with a middle age of 15 years and under (*n* = 205), 5% of papers (*n* = 16) investigated individuals with a middle age between 16 and 19 years, and about 20% of papers (*n* = 69) were conducted in participants with a middle age between 20 and 30 years, with the highest number focussing on the 20–23 year range (*n* = 53). This could be attributed to most papers recruiting from either schools or universities for their study populations, as mentioned above.

#### 3.2.2 Interpersonal determinants

**FIGURE 2:**
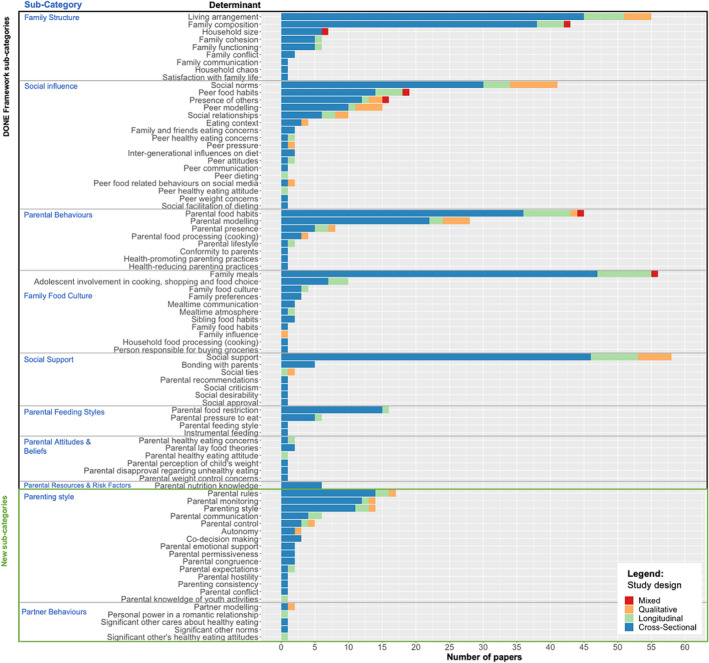
Determinants studied in included papers, presented by Determinants of Nutrition and Eating (DONE) framework sub‐categories and new sub‐categories.

A total of 83 different determinants were identified in the literature. These were grouped into the eight sub‐categories of the DONE framework: (1) family structure (*n* = 122), (2) family food culture (*n* = 83), (3) social influences (*n* = 121), (4) social support (*n* = 69), (5) parental resources and risk factors (*n* = 6), (6) parental attitudes and beliefs (*n* = 8), (7) parental behaviors (*n* = 90), and (8) parental feeding styles (*n* = 24). New determinants were identified in all these sub‐categories, in particular within social influences (11 new determinants) and family food culture (8 new determinants). Two new sub‐categories were identified and suggested as additions to the framework: parenting style (*n* = 74) and partner behaviors (*n* = 6). Table 5 demonstrates how the determinants identified were categorized using the DONE framework and which of the DONE framework determinants lacked published evidence in this age group. Examples mentioned by the framework that were considered to fall outside of the scope of this review have been removed; new examples of determinants are marked by an asterisk (*), and new sub‐categories are shaded in grey. For further details on the range of determinants per paper and categorization, see Table S3. Figure 2 summarizes the distribution of determinants in the included papers.

**TABLE 5:** Mapping the interpersonal determinants in the included papers against the Determinants of Nutrition and Eating (DONE) framework sub‐categories, including two new sub‐categories.

Sub‐category	Explanation	Determinants
Family structure (*n* = 122)	“Composition and cohesion of the family/household”	Caregiving role (*n* = 0) Family cohesion (*n* = 6) Family composition (*n* = 43) Household size (*n* = 7) Family functioning* (*n* = 6) Living arrangement (who you live with)* (*n* = 55) Satisfaction with family life* (*n* = 1) Household chaos* (*n* = 1) Family communication* (*n* = 1) Family conflict* (*n* = 2)
Family food culture (*n* = 83)	“Food culture existing in the family/household”	10 Family food culture (*n* = 4) Family preferences (*n* = 3) Household food processing (cooking) (*n* = 1) Family meals* (*n* = 56) Adolescent involvement in food shopping/meal preparation/food choice* (*n* = 10) Family food habits* (*n* = 1) Family influence* (*n* = 1) Mealtime atmosphere* (*n* = 2) Mealtime communication* (*n* = 2) Person responsible for buy groceries* (*n* = 1) Sibling food habits* (*n* = 2)
Social influences (*n* = 121)	“Diet‐ and eating‐related influences from others in the environment”	Inter‐generational influences on diet (*n* = 2) Decisional power in groups (*n* = 0) Eating occasion (*n* = 0) 22 Peer modelling (*n* = 15) Presence of others (*n* = 16) Social facilitation of dieting (*n* = 1) Social norms (*n* = 41) Social relationships (*n* = 10) Eating context* (*n* = 4) Family and friends eating concerns* (*n* = 2) Peer attitudes* (*n* = 2) Peer communication* (*n* = 1) Peer dieting* (*n* = 1) Peer food habits* (*n* = 19) Peer food‐related behaviors on social media* (*n* = 1) Peer healthy eating attitudes* (*n* = 1) Peer healthy eating concerns* (*n* = 2) Peer pressure* (*n* = 2) Peer weight concerns* (*n* = 1)
Social support (*n* = 69)	“Diet‐ and eating‐related support from others in the environment”	Bonding with parents (*n* = 5) Community recommendations (*n* = 0) Frequency of social contacts (*n* = 0) 39 Parental recommendations (*n* = 1) Peer cooking practices and skills (*n* = 0) Salesperson recommendations (*n* = 0) Social support (*n* = 57) Social ties (*n* = 2) Social approval* (*n* = 1) Social criticism* (*n* = 1) Social desirability* (*n* = 1)
Parental resources and risk factors (*n* = 6)	“Parental resources and constraints relevant for diet and eating”	Parental food market knowledge (*n* = 0) Parental food product knowledge (*n* = 0) 45 Parental nutrition knowledge (*n* = 6) Parental time constraints (*n* = 0)
Parental attitudes and beliefs (*n* = 8)	“Parental thoughts and beliefs about food and eating”	Parental body dissatisfaction (*n* = 0) Parental food ethics (*n* = 0) Parental risk aversion (*n* = 0) Parental lay food theories (*n* = 2) Parental perceived food safety (*n* = 0) Parental perception of child's weight (*n* = 1) Parental weight control concerns (*n* = 1) Parental weight control goal (*n* = 0) 49 Parental healthy eating attitude* (*n* = 1) Parental healthy eating concerns* (*n* = 1) Parental disapproval regarding unhealthy eating* (*n* = 1)
Parental behaviors (*n* = 90)	“Parental food‐ and eating‐related behaviors”	Parental conformity to tradition (*n* = 0) Parental food habits (*n* = 45) Parental food processing (cooking) (*n* = 4) Parental lifestyle (*n* = 2) Parental modelling (*n* = 28) Conformity to parents* (*n* = 1) Health‐promoting parenting practices* (*n* = 1) Health reducing parenting practices* (*n* = 1) Parental presence* (*n* = 8)
Parental feeding styles (*n* = 24)	“How parents go about feeding their children”	Food used as an incentive (*n* = 0) Parental emotional feeding (*n* = 0) 60 Parental food restriction (*n* = 16) Parental instrumental feeding (*n* = 1) Parental portion size habits (*n* = 0) Parental pressure to eat (*n* = 6) Parental feeding style* (*n* = 1)
Parenting style (*n* = 74)	General parenting	64 Parental monitoring* (*n* = 14) Adolescent autonomy* (*n* = 3) Parenting style* (*n* = 14) Co‐decision making* (*n* = 3) Parental communication* (*n* = 6) Parental conflict* (*n* = 1) Parental congruence* (*n* = 1) Parental control* (*n* = 5) Parental emotional support* (*n* = 2) Parental expectations* (*n* = 2) Parental hostility* (*n* = 1) Parental knowledge of youth activities* (*n* = 1) Parental permissiveness* (*n* = 2) Parental rules* (*n* = 17) Parenting consistency* (*n* = 1)
Partners behaviors (*n* = 6)	Partner food‐ and eating‐related behaviors	Partner modelling* (*n* = 2) Partner norms* (*n* = 1) Partner attitudes towards healthy eating* (*n* = 1) Partner cares about healthy eating* (*n* = 1) Personal power in a romantic relationship* (*n* = 1)

#### 3.2.3 Diet quality and eating behaviors

**FIGURE 3:**
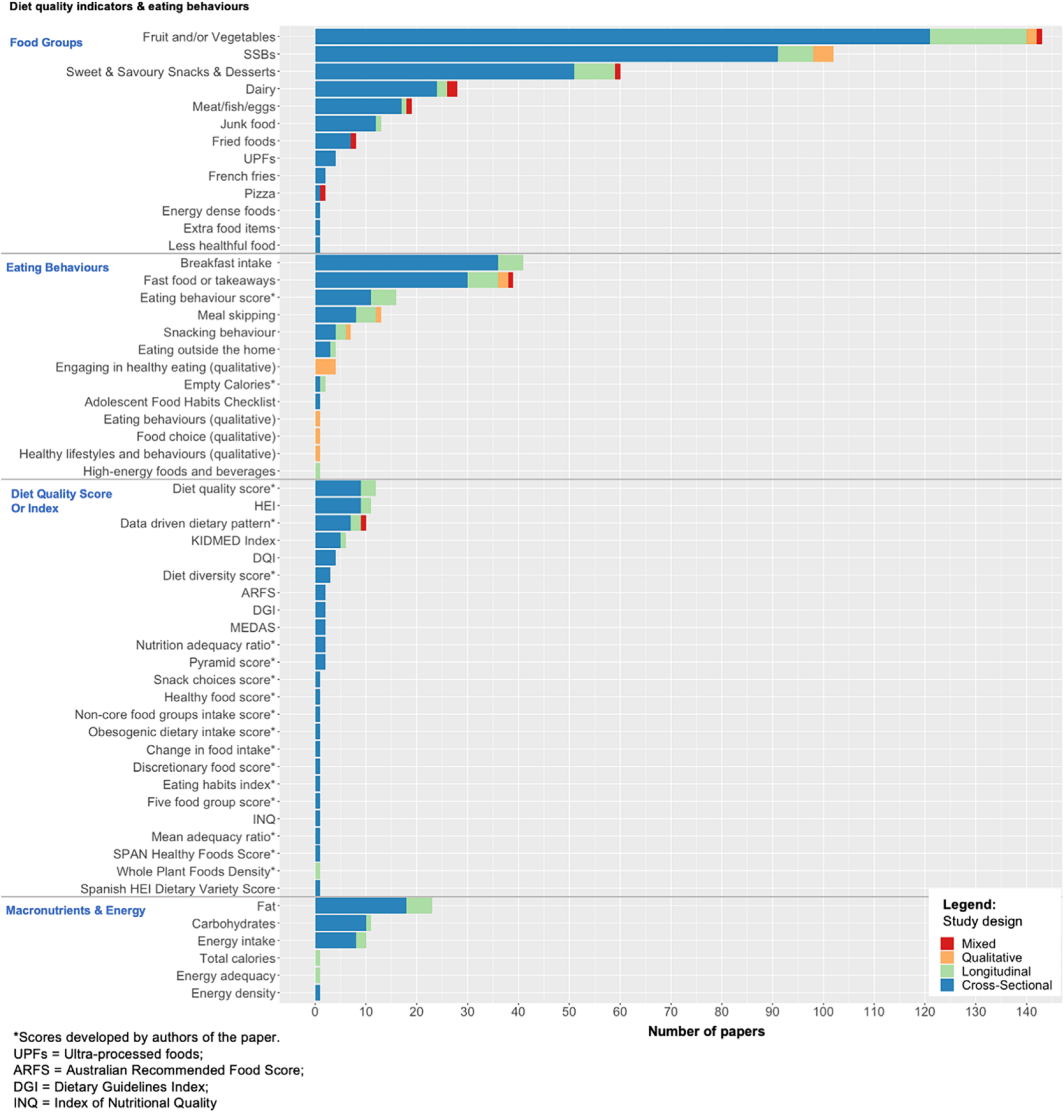
Diet quality and eating behavior measures studied in included papers.

A wide range of diet quality measures and eating behaviors were considered in the included papers, with almost 40% of papers investigating more than one outcome, and the maximum number of outcomes studied in one paper was eight. Intake of fruit and vegetables (*n* = 143) and SSBs (*n* = 102) were most widely reported as indicators of diet quality. Established indices of diet quality were not widely used (*n* = 29), but included the HEI (*n* = 11), KIDMED Index or MEDAS (measures of adherence to the Mediterranean diet) (*n* = 8), and the Diet Quality Index (DQI) (*n* = 4). Over half of papers using a diet quality score or index opted to create their own diet quality or eating behavior scores (*n* = 40). In terms of eating behaviors, fast food and takeaways intake (*n* = 39) and breakfast frequency (*n* = 41) were common measures used; however, snacking behavior was only assessed in seven papers, with many more papers assessing snack food intake (*n* = 60). Figure 3 shows details of the diet quality measures and eating behaviors assessed in the included papers, broadly categorized into (1) food groups (*n* = 384), (2) eating behaviors (*n* = 131), (3) diet quality score or index (*n* = 69), and (4) macronutrients and energy (*n* = 47).

## 4 DISCUSSION

### 4.1 Summary of evidence

The main objective of this review was to summarize the interpersonal determinants of diet quality and eating behaviors that have been studied and identify gaps in the evidence base. In total, 329 papers were included, and 83 determinants were identified. These were categorized within the eight existing DONE framework sub‐categories of the social category within the interpersonal level, and two new sub‐categories were added. Overall, we found that the literature was primarily focused on family structure (e.g., living arrangement, family composition, and household size) and social influences (e.g., peer behaviors, modelling, and social norms). There was less of a focus on DONE sub‐categories such as parental feeding styles, which would typically relate to younger children, which is understandable given our focus on adolescents in this review. There were also few papers focused on parental attitudes and beliefs or parental resources and risk factors and how these might influence adolescents' diets.

A higher number of participants studied were recruited in the USA, with about 30% of these derived from the Project EAT cohort. Younger adolescents (15 years or less) have been the main focus of research in this area (>60%), with much less emphasis on individuals aged 16–19 (~5%) and 20 years or older outside of a university setting (35%). Additionally, more than 80% of papers used a cross‐sectional design, which may not capture the changing nature of the interpersonal determinants studied, particularly in adolescents and young adults where changes in social circumstances are frequent.

The majority of included papers only assessed one aspect of the diet, such as fruit and vegetables or SSB intake, rather than looking at the diet as a whole. Papers that did use a more holistic approach to understanding the diet often calculated their own scores in an attempt to quantify diet quality, as opposed to using established scores such as the DASH^18^ diet or the Mediterranean diet score.^21^ This makes it challenging to compare outcomes across these papers. This may reflect data availability. Although comparability may be improved through the use of validated measures, using an overall diet may not always be the best way to understand interpersonal determinants of behaviors. There have been suggestions that social influences may affect intake of different foods differently, and unpicking these may provide an understanding of which aspects of the diet are distinctively influenced by members in adolescents' social circles.^50^ Therefore, it may be valuable to standardize food group measures further in terms of definitions (e.g., what is considered healthy versus unhealthy foods) and metrics (e.g., frequency of intake, portion sizes).^51^, ^52^

#### 4.1.1 Gaps and suggestions for expanding the interpersonal level and social category of the DONE framework

Although the DONE framework provides a broad summary of potential interpersonal determinants, the determinants assessed in the identified papers did not all map onto this framework. This may be due to the framework not addressing the adolescent population specifically and not distinguishing partners from family/peers. Moreover, it does not address the changing role of parents during adolescence, for example, as adolescents become more involved in food purchasing and preparation. There is a much stronger focus on young children and adulthood without considering the more transitional phase of adolescence. We propose two additional sub‐categories: parenting style and partner behaviors. Parenting style included determinants related to general parenting such as strictness, permissiveness, and monitoring. This new sub‐category was added as these determinants fell outside of the scope of the other sub‐categories (e.g., parental feeding style was too specific to how parents feed their children). The importance of this sub‐category is reflected by a total of 74 papers investigating this topic. Partner behaviors were added as another sub‐category. We distinguished partner relationships from both peer and family relationships. There is an increasing emergence and importance of romantic relationships in adolescence and young adulthood,^53^ yet partners in this age group may not yet be considered “family.”

#### 4.1.2 Transitions and new generations

Emerging adults are experiencing a time of transition, for example, leaving home, starting a job, going to university, and starting a romantic relationship or cohabitation.^54^ These life changes all tie into shifting social factors that may be influencing their diet, demonstrating the variable nature of interpersonal determinants in this age group. However, these changes cannot be properly understood only through cross‐sectional studies alone. This review reveals the scarcity of relevant longitudinal analyses and qualitative studies, which may leave gaps in understanding how the influence of these determinants on diet and eating behaviors is changing over time and therefore contributing to dietary habits later in life. Although qualitative studies may be able to interrogate changing influences over time without a longitudinal design, only three of the 16 qualitative papers included explicitly addressed life transitions. In addition, participants in the included papers were primarily younger than 15 years, with a shortage of research in the 16–24 years age category, who are experiencing these transitions.

Moreover, it is also important to consider interpersonal determinants that are relevant to new generations and how they are experiencing a changing world. Adolescents now have access to their social networks not only in person but also through social media.^55^ In this review, the influence of peer food‐related social media behaviors was only evaluated in one qualitative paper^56^ and one cross‐sectional paper.^57^ A growing number of adolescents have access to different social media platforms, which are often used by young people to share where they eat or where they can find good food.^16^

Therefore, future research should focus on longitudinal and qualitative analyses to understand the transient nature of determinants of diet quality and eating behaviors in this age group, as well as identifying emerging determinants, such as peer social media behaviors, that may not have been considered in previous frameworks or research. A deeper investigation into analyses across multiple timepoints could help provide further context on how the identified determinants may be contributing to the establishment of dietary habits in the long term.

### 4.2 Strengths and limitations of this review

This review's main strength is that it provides a comprehensive overview of the existing literature on interpersonal determinants of diet quality and eating behaviors in 13–30 year olds. The broad search strategy and inclusion criteria allowed for an extensive search to be conducted to include as much relevant literature as possible, including those published in other languages. However, the review was driven by the terms in the search strategy as guided by the DONE framework. Although we attempted to include a wide range of terms for exposures, outcomes, and age group, we may not have captured all relevant papers, particularly if they fell outside of the DONE framework's categories. However, this was mitigated during title/abstract screening by including papers that studied determinants outside of the DONE framework and adapting the inclusion and exclusion criteria accordingly through discussions on relevance with the reviewing team. Moreover, all full texts were double‐screened by reviewers with >80% inter‐rater agreement prior to further discussion. Similarly high agreement was seen during pilot data extraction, allowing for it to be completed by the primary reviewer.

Using the DONE framework to systematically classify determinants helped demonstrate the gaps in the literature, as well as provide an opportunity to evaluate and expand upon this existing theoretical framework, focussing on the adolescent and young adult population. However, categorization of interpersonal determinants within the DONE framework sub‐categories was completed by only one reviewer, and others may approach classification differently, particularly where terminology used differed from the framework examples. Additionally, the framework does not provide a detailed explanation of the definitions of their determinants; therefore, these could have been interpreted differently by other researchers. Moreover, although using the DONE framework was a helpful guide to identify gaps and to provide structure, there is no definitive way of classifying interpersonal determinants, particularly because there was no special focus on the adolescent to young adult period in this framework.

## 5 CONCLUSION

To our knowledge, this review is the first to summarize the broad scope of available evidence investigating the interpersonal determinants of diet quality and eating behaviors in adolescents and young adults aged 13–30 years. The evidence suggests that interpersonal determinants are broadly studied cross‐sectionally, particularly around family structure and social influences. We found fewer papers focused on parental attitudes and beliefs, parental resources and risk factors, or partner behaviors and how these might influence adolescents' diets. The DONE framework could be enhanced with additions that pertain more to the adolescent and young adult population (e.g., partner behaviors), providing a more comprehensive conceptual framework for this area of research.

This review provides an important overview of interpersonal determinants that could be individually systematically reviewed for their strength and direction of association with diet quality and/or eating behaviors. Additionally, it can help guide the direction of future studies and analyses to be more geared towards longitudinal and qualitative investigations, with additional focus on emerging adults experiencing transitions.

## AUTHOR CONTRIBUTIONS

Tanya Braune designed and wrote the review protocol, performed the literature searches, screened titles/abstracts and full texts, extracted and analyzed the data, interpreted results, managed references, and drafted the manuscript. Eleanor M. Winpenny and Jean Adams supported the conceptualization of the study, interpreting results, and drafting the manuscript. Laura Kudlek, Holly A. Harris, Erin Fitzsimons‐West, and Eleanor M. Winpenny participated in the title/abstract screening. Laura Kudlek, Christina Xiao, and Hao Tang participated in the full text screening. Élisabeth Demers‐Potvin contributed to piloting data extraction. All authors reviewed drafts of the manuscript.

## CONFLICT OF INTEREST STATEMENT

No conflicts of interest.

## Supporting information


**Table S1.** Preferred Reporting Items for Systematic reviews and Meta‐Analyses extension for Scoping Reviews (PRISMA‐ScR) Checklist.
**Table S2**. Detailed search strategy for MEDLINE.
**Table S3**. Included papers and categorisation of determinants (for further details on study characteristics, see the Open Science Framework link: https://osf.io/fx7qe).
**Table S4**. Countries studied across all included papers, by study design.
