# Supplementary material for: Interpersonal determinants of diet quality and eating behaviors in people aged 13–30 years: A systematic scoping review
Source: Obes Rev. 2024 Sep 14;26(1):e13835. doi: 10.1111/obr.13835 (PMC11611438; doi:10.1111/obr.13835)
Supplement: Supplementary file 1 — Table S1. Preferred Reporting Items for Systematic reviews and Meta‐Analyses extension for Scoping Reviews (PRISMA‐ScR) Checklist. Table S2. Detailed search strategy for MEDLINE. Table S3. Included papers and categorisation of determinants (for further details on study characteristics, see the Open Science Framework link: https://osf.io/fx7qe). Table S4. Countries studied across all included papers, by study design. [file OBR-26-e13835-s001.pdf]

Supporting Information

Title
Interpersonal determinants of diet quality and eating behaviours in people aged 13-30 years: a systematic scoping review.

Authors
Tanya Braune[1] (TB), Laura Kudlek[1] (LK), Dr Christina Xiao[1] (CX), Hao Tang[1] (HT), Élisabeth Demers-Potvin[2] (EDP), Dr Holly A. Harris (HAH)[1,4], Erin Fitzsimons-West[3] (EFW), Prof Jean Adams[1] (JA), Dr Eleanor M. Winpenny[1] (EMW).

Departments and Institutions
[1]MRC Epidemiology Unit, University of Cambridge, Cambridge, United Kingdom
[2]Centre NUTRISS - Nutrition, santé et société, Institut sur la nutrition et les aliments fonctionnels, Université Laval, Québec, Canada
[3]School of Clinical Medicine, University of Cambridge, Cambridge, United Kingdom
[4]Department of Psychology, Education & Child Studies, Erasmus University Rotterdam, Rotterdam, The Netherlands

Key words
Young people, interpersonal determinants, diet quality, eating behaviours

Running title
Scoping review of interpersonal determinants of diet quality and eating behaviours in young people.

Acknowledgements
We would like to acknowledge the support of Clinical School librarian, Dr Veronica Phillips, in reviewing the search strategy for this scoping review. Additionally, we acknowledge Dr Luiza Ricardo (Portuguese), Shayan Aryannezhad (Persian), Dr Monica Koo (Korean), Dr Jakub Sobiecki (Polish), and Rizka Maulida (Japanese) for their work in translating papers published in languages other than English during data extraction.

Correspondence
Tanya Braune, MRC Epidemiology Unit, University of Cambridge, Level 3 Institute of Metabolic Science, University of Cambridge School of Clinical Medicine, Cambridge CB2 0SL, United Kingdom
Email: tanya.braune@mrc-epid.cam.ac.uk

Potential conflicts of interest
No conflicts of interest.

Abbreviations
MRC, Medical Research Council; BMI, body mass index; DASH, Dietary Approached to Stop Hypertension; WHO, World Health Organisation; PRISMA-ScR, Preferred Reporting Items for Systematic Review and Meta-analysis Protocols Extension for Scoping Reviews; DONE, Determinants of Nutrition and Eating; SEP, socioeconomic position; USA, United States of America; UK, United Kingdom; Project EAT, Project Eating and Activity over Time; HBSC, Health Behaviour in School-Aged Children; HELENA, Healthy Lifestyle in Europe by Nutrition in Adolescence; GSHS, Global School-Based Student Health Survey; FLASHE, Family Life, Activity, Sun, Health and Eating; PeNSE, National School Health Survey; YEP, Youth Eating Patterns; ENDORSE, Environmental Determinants of Obesity in Rotterdam School children; Add Health, National Longitudinal Study of Adolescent Health; GUTS, Growing Up Today Study; SSBs, sugar sweetened beverages; HEI, Healthy Eating Index; DQI, Diet Quality Index; UPFs, Ultra-processed foods; ARFS, Australian Recommended Food Score; DGI, Dietary Guidelines Index; INQ, Index of Nutritional Quality; DTP, Doctoral Training Partnership.

Interpersonal determinants of diet quality and eating behaviours in people aged 13-30 years: a systematic scoping review.

Table S1: Preferred Reporting Items for Systematic reviews and Meta-Analyses extension for Scoping Reviews (PRISMA-ScR) Checklist

SECTION	ITEM	PRISMA-ScR CHECKLIST ITEM	REPORTED ON PAGE #
**TITLE**			
Title	1	Identify the report as a scoping review.	Title Page
**ABSTRACT**			
Structured summary	2	Provide a structured summary that includes (as applicable): background, objectives, eligibility criteria, sources of evidence, charting methods, results, and conclusions that relate to the review questions and objectives.	1
**INTRODUCTION**			
Rationale	3	Describe the rationale for the review in the context of what is already known. Explain why the review questions/objectives lend themselves to a scoping review approach.	1-3
Objectives	4	Provide an explicit statement of the questions and objectives being addressed with reference to their key elements (e.g., population or participants, concepts, and context) or other relevant key elements used to conceptualize the review questions and/or objectives.	3
**METHODS**			
Protocol and registration	5	Indicate whether a review protocol exists; state if and where it can be accessed (e.g., a Web address); and if available, provide registration information, including the registration number.	3
Eligibility criteria	6	Specify characteristics of the sources of evidence used as eligibility criteria (e.g., years considered, language, and publication status), and provide a rationale.	5 (Table 2)
Information sources*	7	Describe all information sources in the search (e.g., databases with dates of coverage and contact with authors to identify additional sources), as well as the date the most recent search was executed.	4
Search	8	Present the full electronic search strategy for at least 1 database, including any limits used, such that it could be repeated.	4 (Supporting information Table 2)
Selection of sources of evidence†	9	State the process for selecting sources of evidence (i.e., screening and eligibility) included in the scoping review.	4-5
Data charting process‡	10	Describe the methods of charting data from the included sources of evidence (e.g., calibrated forms or forms that have been tested by the team before their use, and whether data charting was done independently or in duplicate) and any processes for obtaining and confirming data from investigators.	6
Data items	11	List and define all variables for which data were sought and any assumptions and simplifications made.	6
Critical appraisal of individual	12	If done, provide a rationale for conducting a critical appraisal of included sources of evidence; describe the methods used and how this	N/A

sources of evidence§		information was used in any data synthesis (if appropriate).	
Synthesis of results	13	Describe the methods of handling and summarizing the data that were charted.	6
**RESULTS**			
Selection of sources of evidence	14	Give numbers of sources of evidence screened, assessed for eligibility, and included in the review, with reasons for exclusions at each stage, ideally using a flow diagram.	7
Characteristics of sources of evidence	15	For each source of evidence, present characteristics for which data were charted and provide the citations.	7 (Supporting Information Table 4).
Critical appraisal within sources of evidence	16	If done, present data on critical appraisal of included sources of evidence (see item 12).	N/A
Results of individual sources of evidence	17	For each included source of evidence, present the relevant data that were charted that relate to the review questions and objectives.	Supporting Information Table 4 and link: https://osf.io/fx7qe
Synthesis of results	18	Summarize and/or present the charting results as they relate to the review questions and objectives.	7-9
**DISCUSSION**			
Summary of evidence	19	Summarize the main results (including an overview of concepts, themes, and types of evidence available), link to the review questions and objectives, and consider the relevance to key groups.	9-11
Limitations	20	Discuss the limitations of the scoping review process.	12
Conclusions	21	Provide a general interpretation of the results with respect to the review questions and objectives, as well as potential implications and/or next steps.	13
**FUNDING**			
Funding	22	Describe sources of funding for the included sources of evidence, as well as sources of funding for the scoping review. Describe the role of the funders of the scoping review.	14

JBI = Joanna Briggs Institute; PRISMA-ScR = Preferred Reporting Items for Systematic reviews and Meta-Analyses extension for Scoping Reviews.
* Where *sources of evidence* (see second footnote) are compiled from, such as bibliographic databases, social media platforms, and Web sites.
† A more inclusive/heterogeneous term used to account for the different types of evidence or data sources (e.g., quantitative and/or qualitative research, expert opinion, and policy documents) that may be eligible in a scoping review as opposed to only studies. This is not to be confused with *information sources* (see first footnote).
‡ The frameworks by Arksey and O'Malley (6) and Levac and colleagues (7) and the JBI guidance (4, 5) refer to the process of data extraction in a scoping review as data charting.
§ The process of systematically examining research evidence to assess its validity, results, and relevance before using it to inform a decision. This term is used for items 12 and 19 instead of "risk of bias" (which is more applicable to systematic reviews of interventions) to include and acknowledge the various sources of evidence that may be used in a scoping review (e.g., quantitative and/or qualitative research, expert opinion, and policy document).

From: Tricco AC, Lillie E, Zarin W, O'Brien KK, Colquhoun H, Levac D, et al. PRISMA Extension for Scoping Reviews (PRISMAScR): Checklist and Explanation. Ann Intern Med. 2018;169:467–473. doi: 10.7326/M18-0850.

Interpersonal determinants of diet quality and eating behaviours in people aged 13-30 years: a systematic scoping review.

Table S2: Detailed search strategy for MEDLINE.

**1**	Interpersonal determinants	interpersonal.tw. OR<br>family.tw. OR<br>parental.tw. OR<br>parent.tw. OR<br>parents.tw. OR<br>paternal.tw. OR<br>maternal.tw. OR<br>father*.tw. OR<br>mother*.tw. OR<br>peer*.tw. OR<br>friend*.tw. OR<br>"social influence*".tw. OR<br>"social interaction*".tw. OR<br>"social determinant*".tw. OR<br>"social relationship*".tw. OR<br>"social norm*".tw. OR<br>"eating occasion*".tw. OR<br>"shared meal*".tw. OR<br>"social support".tw. OR<br>"caregiver* recommendation*".tw. OR<br>"social ties".tw. OR<br>"social contact*".tw. OR<br>"social network*".tw. OR<br>"social desirability".tw. OR<br>"caregiver* resource*".tw. OR<br>"caregiver* attitude*".tw. OR<br>"caregiver* belief*".tw. OR<br>"caregiver model*".tw. OR<br>"caregiver* cook*".tw. OR<br>"feeding style*".tw. OR<br>"caregiver* feeding style*".tw. OR<br>"food restriction*".tw. OR<br>"food parenting practices".tw. OR<br>"home food".tw. OR<br>"home food environment".tw. OR<br>"social environment".tw. OR<br>psychosocial.tw. OR<br>"psycho-social".tw. OR<br>"household socioeconomic status".tw. OR<br>"household socio economic status".tw. OR<br>"household socioeconomic position".tw. OR<br>"household socio economic position".tw. OR<br>"household socioeconomic class".tw. OR<br>"household socio economic class".tw. OR<br>Social Determinants of Health [MeSH]
**2**	Diet Outcomes	food*.tw. OR<br>beverage*.tw. OR<br>diet*.tw. OR<br>fruit*.tw. OR<br>vegetable*.tw. OR<br>dairy.tw. OR<br>"soft drink*".tw. OR<br>"fizzy drink*".tw. OR<br>soda*.tw. OR<br>SSB.tw. OR

		SSBs.tw. OR<br>“sugar sweetened beverage*”.tw. OR<br>“sugar-sweetened beverage*”.tw. OR<br>sugar.tw. OR<br>“unhealthy eating habit*”.tw. OR<br>“unhealthy food choice*”.tw. OR<br>“healthy eating habit*”.tw. OR<br>“healthy food choice*”.tw. OR<br>“energy dens*”.tw. OR<br>“energy-dens*”.tw. OR<br>“junk food”.tw. OR<br>“nutrient-poor”.tw. OR<br>“nutrient poor”.tw. OR<br>“nutrient rich”.tw. OR<br>“nutrient-rich”.tw. OR<br>“ultra processed food”.tw. OR<br>“ultra-processed food”.tw. OR
	Eating behaviours	cook*.tw. OR<br>“food preparation”.tw. OR<br>“eating behavio*”.tw. OR<br>meal*.tw. OR<br>snack*.tw. OR<br>“dining out”.tw. OR<br>breakfast.tw. OR<br>dinner.tw. OR<br>lunch.tw. OR<br>supper.tw. OR<br>“fast food”.tw. OR<br>fastfood.tw. OR<br>“fast-food”.tw. OR<br>restaurant.tw. OR<br>“take away”.tw. OR<br>takeaway.tw. OR<br>takeout.tw. OR<br>“take out”.tw. OR<br>“take-out”.tw. OR<br>“eating out”.tw. OR<br>“away from home”.tw. OR<br>“out of home”.tw. OR<br>“away-from-home”.tw. OR<br>“out-of-home”.tw. OR<br>“home cook*”.tw. OR<br>“home prepar*”.tw. OR<br>Food [MeSH] OR<br>Diet [MeSH] OR<br>Beverages [MeSH] OR
**3**	Age range	adolescen*.tw. OR<br>teen*.tw. OR<br>student*.tw. OR<br>“young adult*”.tw. OR<br>“early adult*”.tw. OR<br>“emerging adult*”.tw. OR<br>youth*.tw. OR<br>“young people”.tw. OR<br>“young person”.tw. OR<br>freshm*.tw. OR

		Adolescent [MeSH]
**4**	Study type	observational.tw. OR<br>“cross-sectional”.tw. OR<br>“cross sectional”.tw. OR<br>cohort.tw. OR<br>longitudinal.tw. OR<br>prospective.tw. OR<br>“follow-up stud*”.tw. OR<br>“follow up stud*”.tw. OR<br>Observational Study [MeSH] OR<br>Longitudinal [MeSH] OR<br>Cross-Sectional [MeSH]
**5**	Additional filters	Humans [Limits]
**6**		1 AND 2 AND 3 AND 4 AND 5

Interpersonal determinants of diet quality and eating behaviours in people aged 13-30 years: a systematic scoping review.
Table S3: Included papers and categorisation of determinants (for further details on study characteristics, see the Open Science Framework link: https://osf.io/fx7qe).

Glossary
FV = Fruit and Vegetables
SSBs = Sugar-Sweetened Beverages
DQI = Diet Quality Index
ARFS = Australian Recommended Food Score
UPFs = Ultra-Processed Foods
HEI = Healthy Eating Index
MEDAS = Adherence to the Mediterranean Diet
DGI = Dietary Guidelines Index
EHI = Eating Habits Index
CS = Cross-sectional
L = Longitudinal
Qual = Qualitative
*Scores developed by authors

Reference	Country	Study design	Setting	n participants	Middle-age	Outcome(s)	Determinant(s)	Determinant Sub-Category									
								Family structure	Family food culture	Social influence	Social support	Parental Resources & Risk Factors	Parental attitudes & beliefs	Parental behaviours	Parental feeding styles	Parenting style	Partner behaviours
Abar et al[1]	USA	L	Households	5419	13	FV	Family routines, perceived parental knowledge of youth activities		✓							✓	
Abizari and Ali[2]	Ghana	CS	School	366	15	Data driven dietary pattern*	Living with parents	✓									
Agustina et al[3]	Indonesia	CS	City/region	238	15	DQI for Adolescents	Family size	✓									
Al-Naggar et al[4]	Malaysia	CS	University	1100	22	FV	Living status (with parents, with roommates)	✓									
Al-Otaibi[5]	Saudi Arabia	CS	University	960	21	FV	Household member's won't eat FV		✓								
Ali et al[6]	Jordan	CS	School	1013	15	Breakfast intake	Mother's encouragement to have breakfast				✓						
Alimoradi et al[7]	Iran	CS	City/region	553	16	Fast food	Family's interest in fast food, friends' effect on fast food intake, eating companion (alone, friends or family)		✓	✓							

Reference	Country	Study design	Setting	n participants	Middle-age	Outcome(s)	Determinant(s)	Determinant Sub-Category									
								Family structure	Family food culture	Social influence	Social support	Parental Resources & Risk Factors	Parental attitudes & beliefs	Parental behaviours	Parental feeding styles	Parenting style	Partner behaviours
Almutairi et al[8]	Saudi Arabia	CS	University	1656	25	Eating Behaviour Score*	Family structure, place of residence	✓									
Alolabi et al[9]	Syria	CS	University	728	22	Eating Behaviour Score*	Place of accommodation (family, friends, alone)	✓									
Alsunni and Badar[10]	Saudi Arabia	CS	School	367	22	FV	Residency (living with family, alone or sharing)	✓									
Aluqmany et al[11]	Saudi Arabia	CS	School	600	17	Energy drinks (SSBs)	Family member/close friend intake		✓	✓							
Alzahrani et al[12]	Saudi Arabia	CS	University	378	21	Eating Behaviour Score*	Living arrangement (alone vs family), number of family members	✓									
Ambrosini et al[13]	Australia	CS	Birth cohort	1631	14	Data driven dietary pattern*	Single- or dual-parent family	✓									
Anastacio-Landa et al[14]	Mexico	CS	School	296	13	SSBs	Parental hostility									✓	
Appannah et al[15]	Australia	L	Birth cohort	1377	14	Data driven dietary pattern*	Two-parent family structure, family functioning	✓									
Arabi-Mianrood et al[16]	Iran	CS	University	248	21	Eating Behaviour Score*	Relationship score (getting on with parents, friends, siblings)			✓							
Arcan et al[17]	USA	L	School	509 pairs	15	FV, dairy	Parents' intake							✓			
Arora et al[18]	India	CS	School	1814	14	Breakfast intake	Peer's influence (peers care about eating healthy food), parents' influence (parental encouragement to eat healthy food)			✓	✓						
Arroyo et al[19]	USA	L	University	380	20	Eating Behaviour Score*	Co-rumination (talking with friends about weight)			✓							
Ashton et al[20]	Australia	Qual	City/region	61	20	Engaging in healthy eating (qualitative)	Social influences - social inclusion and social environment including peers and family. Barriers - social context of eating, emphasis on the role of peers, social norms for men			✓							
Azeredo et al[21]	Brazil	CS	School	109104	13	Diet Quality Score*	Meals with parents		✓								

Interpersonal determinants of diet quality and eating behaviours in people aged 13-30 years: a systematic scoping review.

Reference	Country	Study design	Setting	n participants	Middle-age	Outcome(s)	Determinant(s)	Determinant Sub-Category									
								Family structure	Family food culture	Social influence	Social support	Parental Resources & Risk Factors	Parental attitudes & beliefs	Parental behaviours	Parental feeding styles	Parenting style	Partner behaviours
Backman et al[22]	USA	L	School	780	16	Total calories, fat, FV	Normative beliefs (mother, sibling, friend, father, coach), support and encouragement				✓						
Badrasawi et al[23]	Palestine	CS	City/region	193	13	Breakfast intake	Family type (nuclear or extended), number of family members	✓									
Bagherniya et al[24]	Iran	CS	City/region	172	13	FV, junk food	Nutritional social support				✓						
Baig et al[25]	Oman	CS	School	3468	14	Diet Quality Score*	Parental involvement (check homework, understand problems, know what you're doing in your free time, go through your things)									✓	
Bailey et al[26]	India	Qual	City/region	38	29	Food choice (qualitative)	Family influence		✓								
Baker et al[27]	USA	L	School	279	15	Eating Behaviour Score*	Social norms			✓							
Baldwin et al[28]	Australia	CS	City/region	1005	21	ARFS	Social eating scale (social influence on eating)			✓							
Baltaci et al[29]	USA	CS	City/region	191 dyads	12	FV, SSBs, snacks, fast food	Father's food parenting practices (setting expectations/limits, role modelling, home availability), family meals		✓					✓			
Barco Leme and Tucunduva Philippi[30]	Brazil	CS	School	253	16	FV, dairy, meat/fish/eggs, sweet and savoury snacks	Parents support, family meals		✓		✓						
Bau et al[31]	Germany	CS	School	1519	12	Diet Quality Score*	Family situation (single or dual parent)	✓									
Bauer et al[32]	USA	CS	School	253 dyads	15	SSBs, FV	Parental intake, family meal frequency, encouragement to eat healthy		✓		✓			✓			
Bauer et al[33]	USA	L	School	806	12	Fast food	Family meals, maternal/paternal concern with healthy eating, friends' concern for healthy eating, friends dieting, adult supervision (during meals)		✓	✓			✓	✓			
Befort et al[34]	USA	CS	City/region	228	15	FV, fat	Home availability, meals with family		✓								
Benetou et al[35]	Greece	CS	School	3525	13	Diet Quality Score*	Eating with family		✓								

Reference	Country	Study design	Setting	n participants	Middle-age	Outcome(s)	Determinant(s)	Determinant Sub-Category									
								Family structure	Family food culture	Social influence	Social support	Parental Resources & Risk Factors	Parental attitudes & beliefs	Parental behaviours	Parental feeding styles	Parenting style	Partner behaviours
Berge et al[36]	USA	CS	School	58 sibling pairs	14	FV, fast food, SSBs, breakfast intake	Parental intake, sibling intake		✓					✓			
Berge et al[37]	USA	CS	School	585	24	FV	Family meals		✓								
Berge et al[38]	USA	CS	School	2108	14	FV, SSBs, fat, breakfast intake, fast food	Adolescent/mother/father involvement in meal preparation		✓					✓			
Berge et al[39]	USA	CS	School	1212		FV	Significant other cares about healthy eating										✓
Berge et al[40]	USA	CS	School	200 families	14	FV, fast food, SSBs, breakfast intake	Resident and non-resident parental behaviours							✓			
Berge et al[41]	USA	CS	School	2348	14	FV, breakfast intake, SSBs, fast food	Parent weight-related conversations about eating						✓				
Berge et al[42]	USA	CS	School	40	14	FV	Characteristics and frequency of family meals, family functioning	✓	✓								
Berge et al[43]	USA	L	School	2516	15	FV	Parenting style (authoritative, authoritarian, permissive, neglectful)									✓	
Bibi Nabihah et al[44]	Malaysia	CS	City/region	300	26	FV	Influence of family			✓							
Billon et al[45]	France	CS	Clinical	398 families	14	Breakfast intake	Father's, mother's and siblings intake		✓					✓			
Bohara et al[46]	Nepal	CS	City/region	538	16	Junk food	Family type (nuclear or joint), living status (parents or others), eating context	✓		✓							
Borlu et al[47]	Turkey	CS	University	246	24	FV	Number of people in the household, accommodation type (home or dorm)	✓									
Bourdeaudhuij and Oost[48]	Belgium	CS	City/region	104 dyads	15	Healthy Food Score*, fat, FV, SSBs, snacks	Social support, family modelling, friends modelling, food interactions restriction/obligation rules			✓	✓			✓	✓	✓	
Boylan et al[49]	USA	L	School	305	14	FV	Parental supervision, expectations and communication									✓	
Brindal et al[50]	Australia	Mixed	City/region	157	20	Fast food	Eating in groups			✓							
Bruening et al[51]	USA	CS	School	2043	14	SSBs, fast food	Friend group and best friend intake			✓							

Reference	Country	Study design	Setting	n participants	Middle-age	Outcome(s)	Determinant(s)	Determinant Sub-Category									
								Family structure	Family food culture	Social influence	Social support	Parental Resources & Risk Factors	Parental attitudes & beliefs	Parental behaviours	Parental feeding styles	Parenting style	Partner behaviours
Burgess-Champoux et al[52]	USA	L	School	677	12	FV, SSBs, meal skipping, fast food, total energy	Family meals		✓								
Burke et al[53]	Australia	L	City/region	219	9	Fat, total energy	Parental intake							✓			
Campbell et al[54]	Australia	CS	Birth cohort	350	13	Snacks, high-energy fluids (SSBs), takeaway foods	Parenting style (authoritarian, permissive, indulgent), mother's and father's intake, parental pressure to eat, family conflict, praise child for eating healthy, parenting consistency	✓			✓			✓	✓	✓	
Chan et al[55]	Australia	CS	City/region	133	24	Five Food Group Score*, discretionary food score*	Social context (social interaction), eating companion			✓							
Chan et al[56]	Australia	CS	City/region	133	24	Energy density	Social context (social interaction)			✓							
Chang et al[57]	Taiwan	CS	University	606	23	Energy drinks (SSBs)	Live away from parents' home	✓									
Chansukree and Rungjindarat[58]	Thailand	CS	University	1200		Adapted Australian DGI	Social support from parents and peers				✓						
Chaves et al[59]	Brazil	CS	School	53274	14	FV, SSBs, snacks	Family composition, parental monitoring, main meal with family	✓	✓							✓	
Cheikh Ismail et al[60]	United Arab Emirates	CS	University	529	23	Eating Behaviour Score*	Residential type (with family or not)	✓									
Chen et al[61]	USA	CS	School	743	16	Data driven dietary pattern*	Family structure (mother, father, both)	✓									
Choi and Choi[62]	South Korea	CS	School	510	17	Breakfast intake, breakfast type (fast food)	Person they eat out with (family, friend or alone), persons who have breakfast together (all family members, father/mother, brothers/sisters, alone), who prepares breakfast (mother, father, myself)		✓	✓					✓		
Christofaro et al[63]	Brazil	CS	City/region	2526	15	FV, fried foods, snacks, SSBs	Mother's and father's behaviours							✓			
Chung et al[64]	USA	CS	School	5248	12	Breakfast intake, SSBs	Living with parents (dual vs single), communication with parents, monitoring by parents, spending time with friends,	✓		✓						✓	

Reference	Country	Study design	Setting	n participants	Middle-age	Outcome(s)	Determinant(s)	Determinant Sub-Category									
								Family structure	Family food culture	Social influence	Social support	Parental Resources & Risk Factors	Parental attitudes & beliefs	Parental behaviours	Parental feeding styles	Parenting style	Partner behaviours
							number of friends, communication with friends										
Cluskey and Grobe[65]	USA	Qual	University	19	19	Healthy lifestyles and behaviours (qualitative)	Linked Lives — Others (eg, life partners, roommates) provide a lifestyle environment that influences and models behaviors. Transition — A life change in social roles or responsibilities			✓							✓
Cornelius et al[66]	USA	L	City/region	157 couples	17	Healthy eating behaviours	Relationship power (personal power in a relationship)										✓
Cusatis et al[67]	USA	CS	School	242	16	Pyramid score*, Diet Quality Score*	Conformity to parents							✓			
Cutler et al[68]	USA	L	School	2322	15	FV, snacks	Family meal frequency, parental and peer support		✓		✓						
Darfour-Oduro et al[69]	Dominica, Egypt, Fiji, Grenada, Indonesia, Jamaica, Jordan, Malaysia, Malawi, Maldives, Mongolia, Philippines, Thailand	CS	School	89843	15	FV	Parental supervision, connectedness, bonding				✓					✓	
De Bruijn et al[70]	The Netherlands	CS	Clinical	208	15	SSBs	Parenting practices regarding soft drink consumption (measure of parental strictness)								✓		

Reference	Country	Study design	Setting	n participants	Middle-age	Outcome(s)	Determinant(s)	Determinant Sub-Category									
								Family structure	Family food culture	Social influence	Social support	Parental Resources & Risk Factors	Parental attitudes & beliefs	Parental behaviours	Parental feeding styles	Parenting style	Partner behaviours
De la Haye et al[71]	Australia	L	School	378	13	Junk food	friends' food intake (descriptive peer norms, injunctive peer norms, descriptive adult norms)			✓							
De Souza et al[72]	Brazil	CS	School	102072	15	Data driven dietary pattern*	Household composition (single vs dual parent or without)	✓									
DeJong et al[73]	The Netherlands	CS	School	1089	14	Breakfast intake	Subjective norm, modelling by parents and friends, meals with parents		✓	✓				✓			
Delbosq et al[74]	Italy	CS	School	2145	14	FV, snacks, SSBs, breakfast intake	Family meals, family support, mother-child communication, mother's monitoring (general)		✓		✓					✓	
Deliens et al[75]	Belgium	CS	University	425	21	Energy drinks (SSBs)	Social norm, social support, modelling from partner/parents/friends, family rules			✓	✓			✓		✓	✓
Deliens et al[76]	Belgium	CS	University	185	20	FV, fat	Residency (student residence or home), social support, social criticism, social norm, modelling	✓		✓	✓						
Deliens et al[77]	Belgium	Qual	University	35	20	Eating behaviours (qualitative)	Parental control, social support from family and friends, peer pressure, living arrangement	✓		✓	✓					✓	
Demissie et al[78]	USA	CS	School	11458	16	FV, dairy, fried foods, pizza, SSBs	Eating dinner with parent/guardian		✓								
Dewi et al[79]	Indonesia	CS	School	375	16	Diet Quality Score*	Household size, family type, maternal nutrition literacy, mother's eating habits/norms, subjective norms	✓		✓		✓					
Dickens and Ogden[80]	UK	L	School	93 dyads	18	Eating Behaviour Score*	Parental control (overt, covert, pressure to eat), parental modelling							✓		✓	
Doegah and Acquah[81]	Ghana	Qual	School	16	21	Engaging in healthy eating (qualitative)	Social support, upbringing				✓					✓	

Reference	Country	Study design	Setting	n participants	Middle-age	Outcome(s)	Determinant(s)	Determinant Sub-Category									
								Family structure	Family food culture	Social influence	Social support	Parental Resources & Risk Factors	Parental attitudes & beliefs	Parental behaviours	Parental feeding styles	Parenting style	Partner behaviours
Dong et al[82]	China	L	City/region	5201 dyads	12	Eating outside the home, snacking behaviour, meat/fish/eggs	Parent diet, household structure	✓						✓			
Dwyer et al[83]	USA	CS	Online	1443 dyads	14	FV	Parental intake							✓			
El Tantawi et al[84]	Saudi Arabia	CS	City/region	478	13	Snacks, SSBs	Mother's/father's/friend's practices			✓				✓			
Elstgeest et al[85]	Australia	L	Birth cohort	6534	27	Data driven dietary pattern*	Change in living arrangement	✓									
Endalifer et al[86]	Ethiopia	CS	School	411	16	Dietary diversity	Family size	✓									
Eto et al[87]	Japan	Mixed	City/region	598	13	Data driven dietary pattern*	Family structure (family size, family form, number of siblings), family meals	✓	✓								
Feldman et al[88]	USA	CS	School	4746	14	FV, SSBs, fried foods, snacks, calories	Family meals with or without TV		✓								
Ferrara et al[89]	Italy	L	University	2001	22	Eating Behaviour Score*	Relationship status (live with family, live alone, other)	✓									
Feunekes et al[90]	The Netherlands	Mixed	School	361 networks	15	Meat/fish/eggs, dairy, pizza, fried foods, snacks, dairy, FV	Intake resemblance between parents and friends			✓				✓			
Figueroa et al[91]	USA	CS	Online	1649 dyads	14	SSBs	Mother's and father's SSB intake							✓			
Fink et al[92]	USA	CS	Households	1992	14	FV, SSBs	Family meal frequency		✓								
Fismen et al[93]	Norway	CS	School	4475	13	FV, snacks, SSBs	Family structure (single/dual/step parents or other)	✓									
Fleary and Ettienne[94]	USA	CS	Online	1859 dyads	14	FV, junk food, SSBs	Food parenting practices (encouragement, rules/limits), parental eating behaviours				✓			✓	✓	✓	
Fondevila-Gascón et al[95]	Spain	CS	University	161	21	UPFs	Residence type (living away from family)		✓								
Forleo et al[96]	Italy	CS	University	548	25	Meat/fish/eggs, dairy, FV, snacks, SSBs	Living with family	✓									
Franko et al[97]	USA	CS	City/region	2379	9	SSBs, breakfast intake, dairy, FV	Family cohesion	✓									

Interpersonal determinants of diet quality and eating behaviours in people aged 13-30 years: a systematic scoping review.

Reference	Country	Study design	Setting	n participants	Middle-age	Outcome(s)	Determinant(s)	Determinant Sub-Category									
								Family structure	Family food culture	Social influence	Social support	Parental Resources & Risk Factors	Parental attitudes & beliefs	Parental behaviours	Parental feeding styles	Parenting style	Partner behaviours
French et al[98]	USA	CS	School	4745	15	Fast food	Family eats meals together, parent concerns about healthy eating, peer health and weight concern		✓	✓				✓			
Fu et al[99]	China	CS	School	3595	14	FV, SSBs, junk food	Parental control, modelling, co-decision making							✓		✓	
Galimov et al[100]	Germany	L	School	6902	13	Energy drinks (SSBs)	Peer usage			✓							
Gan et al[101]	Malaysia	CS	School	421	13	SSBs	Peer social influence, parenting practices (restrictive food parenting)			✓					✓		
Ganasegeran et al[102]	Malaysia	CS	University	132	22	Eating Behaviour Score*	Living arrangement (with family)	✓									
Garrido-Fernandez et al[103]	Spain	CS	School	8068	15	Breakfast intake, snacks	Family type	✓									
Gebremariam et al[104]	Norway	CS	School	742	13	SSBs, snacking behaviour, FV	Parental modelling, parental rules, accessibility							✓		✓	
Gebremariam et al[105]	Norway	CS	School	706	13	Breakfast intake	Parental modelling, rules and co-participation at breakfast							✓		✓	
Gebremariam et al[106]	Norway	CS	School	440		SSBs, FV	Parental rules related to SSBs/FV									✓	
Gevers et al[107]	The Netherlands	CS	School	329	13	Snacks	General parenting (nurturance, structure, behavioural control, coercive control, overprotection), restrictive snacking rules, parental congruence								✓	✓	
Gillman et al[108]	USA	CS	City/region	16202	11	FV, dairy, meat/fish/eggs, snacks, fried foods, eating outside of the home, SSBs, fat	Family meal frequency		✓								
Glozah and Pevalin[109]	Ghana	CS	School	770	16	Eating Behaviour Score*	Perceived social support from family				✓						

Interpersonal determinants of diet quality and eating behaviours in people aged 13-30 years: a systematic scoping review.

Reference	Country	Study design	Setting	n participants	Middle-age	Outcome(s)	Determinant(s)	Determinant Sub-Category									
								Family structure	Family food culture	Social influence	Social support	Parental Resources & Risk Factors	Parental attitudes & beliefs	Parental behaviours	Parental feeding styles	Parenting style	Partner behaviours
Goñi Murillo et al[110]	Spain	CS	School	465	17	Diet diversity score*	Family meal frequency, involvement in family food choice, pressure to eat		✓						✓		
Graham et al[111]	USA	CS	University	1201	21	FV	Family modelling, friends modelling			✓				✓			
Granner et al[112]	USA	CS	School	843	13	FV, snack choices score*	Peer/family normative beliefs, family dinners, social food attributes (social influence)		✓	✓			✓				
Grunseit et al[113]	Australia	Qual	University	51	25	Takeaway foods	Social support, living with parents, social context and interactions	✓		✓	✓						
Gulati et al[114]	India	CS	City/region	1800 dyads	13	FV, meat/fish/eggs, dairy, carbohydrates, sweet and savours snacks, SSBs	Mother's intake							✓			
Gummeson et al[115]	Sweden	CS	City/region	218	13	Dairy	Subjective norms, normative beliefs, friends influences			✓			✓				
Haddad and Sarti[116]	Brazil	CS	School	209601	14	Diet Quality Score*	Family status (living with both parents or other)	✓									
Haddad et al[117]	Brazil	CS	School	172560		Data driven dietary pattern*	Meals with parents, living with both parents (family type)	✓	✓								
Haidar et al[118]	USA	CS	School	6716	14	FV, SSBs, SPAN Healthy Foods Score*	Parental disapproval regarding unhealthy eating, parent/peer support				✓		✓				
Haines et al[119]	USA	CS	City/region	6382	20	Fast food, SSBs	Family functioning, family structure, parent-adolescent relationships	✓			✓						
Haley et al[120]	Canada	L	School	1096 across 3 years	13	Diet Quality Score*	Family size	✓									
Hallström et al[121]	Austria, Belgium, France, Germany, Greece, Hungary,	CS	School	2672	14	Breakfast intake, SSBs, dairy, meat/fish/eggs, FV, snacks	Family structure (single parent, shared care or traditional)	✓									

Reference	Country	Study design	Setting	n participants	Middle-age	Outcome(s)	Determinant(s)	Determinant Sub-Category									
								Family structure	Family food culture	Social influence	Social support	Parental Resources & Risk Factors	Parental attitudes & beliefs	Parental behaviours	Parental feeding styles	Parenting style	Partner behaviours
	Italy, Spain, Sweden																
Hamade et al[122]	Japan	CS	University	75	21	Fat, carbohydrates, FV, meat/fish/eggs, dairy, sweet and savoury snacks, SSBs	Living with family vs alone, cooking behaviours and skills	✓	✓								
Hanson et al[123]	USA	CS	School	902 dyads	15	FV, dairy	Household availability, served at meals, parental intake							✓			
Harker et al[124]	Germany	CS	University	305	21	FV, dairy, meat/fish/eggs	Residence (dependent or independent)	✓									
Hasan et al[125]	Bangladesh	CS	School	213	15	SSBs	Parent intake of SSBs, friend intake of SSBs			✓				✓			
Hassan et al[126]	Malaysia	CS	City/region	300	21	Eating Behaviour Score*	Place of living (hostel or others)	✓									
Hattersley et al[127]	Australia	Qual	University	35	24	SSBs	Social cues for consumption (contexts in which young adults socialise, family influence)			✓							
Haugland et al[128]	Norway	CS	City/region	13269	15	Meal skipping, breakfast intake, FV, meat/fish/eggs, snacks, SSBs, energy drinks	Parental monitoring (general), parental emotional support, parent-offspring conflict									✓	
Hawkins et al[129]	UK	CS	University	369	22	FV, snacks, SSBs	Descriptive and injunctive norms			✓							
Hayek et al[130]	Lebanon	L	School	341	16	KIDMED score, breakfast intake, snacking behaviour, FV, snacks, fast food	Parenting style (neglectful, permissive, authoritarian, authoritative)									✓	
Hong et al[131]	South Korea	CS	School	222662		Meal skipping	Family members (living with parents or not)	✓									
Hormenu[132]	Ghana	CS	School	1311	12	Data driven dietary pattern*	Parental communication									✓	
Huamancayo-Espíritu and Perez-Cardenas[133]	Peru	CS	University	371	23	FV	Living with parents in charge of food	✓									

Reference	Country	Study design	Setting	n participants	Middle-age	Outcome(s)	Determinant(s)	Determinant Sub-Category									
								Family structure	Family food culture	Social influence	Social support	Parental Resources & Risk Factors	Parental attitudes & beliefs	Parental behaviours	Parental feeding styles	Parenting style	Partner behaviours
Ilesanmi et al[134]	Nigeria	CS	School	294	15	FV	Living arrangement, parent family type (monogamous vs polygamous), number of siblings, supportive social environment	✓			✓						
Imoisili et al[135]	USA	CS	Online	1555 dyads	14	SSBs	Parent SSB intake							✓			
Isabirye et al[136]	Uganda	CS	City/region	598	14	Diet diversity score*	Living with both / one parent / guardian, household size	✓									
Johnson et al[137]	USA	CS	School	1457	14	FV	Family and friends support				✓						
Jones and Robinson[138]	UK	L	University	340	21	Snacks, SSBs	Peer norms			✓							
Jorgensen et al[139]	Denmark	CS	School	6269	13	Breakfast intake	Family structure (traditional, single-parent, reconstructed)	✓									
Karimi-Shahanjarini et al[140]	Iran	CS	School	739	13	Junk food	Perceived parental control of junk food intake, subjective norm			✓					✓		
Kassem and Lee[141]	USA	CS	School	564	15	SSBs	Subjective norm			✓							
Kawasaki et al[142]	Japan	CS	University	203	20	FV, meat/fish/eggs	Resident status	✓									
Kelles and Adair[143]	Philippines	L	City/region	1885 pairs	11	Energy adequacy, fat, carbohydrates	Mother's dietary intake							✓			
Keski-Rahkonen et al[144]	Finland	CS	Birth cohort	5448 twins and their parents (4660)	16	Breakfast intake	Mother's and father's breakfast eating,							✓			
Khalaf et al[145]	Saudi Arabia	CS	University	663	20	FV, breakfast intake, dairy, SSBs, fast food, French fries, snacks, energy drinks (SSBs)	Number of siblings	✓									
Kilanowski[146]	USA	Qual	City/region	24	13	FV	Mothers influence on healthy eating, friends encourage unhealthy food choices				✓				✓		

Reference	Country	Study design	Setting	n participants	Middle-age	Outcome(s)	Determinant(s)	Determinant Sub-Category									
								Family structure	Family food culture	Social influence	Social support	Parental Resources & Risk Factors	Parental attitudes & beliefs	Parental behaviours	Parental feeding styles	Parenting style	Partner behaviours
Kim et al[147]	USA	CS	City/region	106	14	Calories, carbohydrates, fat	Perceived parenting style (nurturing vs control)									✓	
Kim et al[148]	Korea	CS	University	476	21	Meal skipping, meat/fish/eggs, dairy, snacks, SSBs, fast food	Type of residence (parents, self-boarding, lodging, dorm)	✓									
Klaiber et al[149]	Canada	L	University	67	20	HEI	Perceived social support, social integration			✓	✓						
Kobayashi et al[150]	Japan	CS	University	1336 families	18	Carbohydrates, FV, SSBs, meat/fish/eggs, energy intake, eating outside of the home, sweet and savoury snacks	Cohabitation with mother and grandmother	✓		✓							
Kobayashi et al[151]	Japan	CS	University	4017	18	Fat	Living status (with family or alone)	✓		✓							
Koehn et al[152]	UK	Qual	City/region	16	17	Engaging in healthy eating (qualitative)	Changes in priorities - greater independence, external influences - parents cook, peer modelling, social activities			✓				✓		✓	
Kremers et al[153]	The Netherlands	CS	School	643	16	FV	Parenting style (authoritative, authoritarian, indulgent, neglectful)									✓	
Kumari and Jain[154]	India	CS	School	100	14	FV, sweet snacks	Family type (joint, nuclear)	✓									
Kwon et al[155]	South Korea	CS	School	186227	15	Fast food	Household type (single, dual or no parents)	✓									
LaCaille et al[156]	USA	Qual	University	49	19	Engaging in healthy eating (qualitative)	Living off campus, cooking & shopping for self , social support, friends and family influence	✓	✓	✓	✓						
Lahmann et al[157]	Australia	CS	University	2017	20	Energy intake, fat, carbohydrates, sweet snacks, FV, dairy, meat/fish/eggs	Mother resemblance in dietary intake							✓			
Lally et al[158]	UK	CS	School	264	16	FV, SSBs, snacks	Injunctive/actual social norms			✓							

Reference	Country	Study design	Setting	n participants	Middle-age	Outcome(s)	Determinant(s)	Determinant Sub-Category									
								Family structure	Family food culture	Social influence	Social support	Parental Resources & Risk Factors	Parental attitudes & beliefs	Parental behaviours	Parental feeding styles	Parenting style	Partner behaviours
Larson et al[159]	USA	CS	School	2507	14	FV, dairy, SSBs, energy intake, fat	Breakfast with family frequency		✓								
Larson et al[160]	USA	CS	School	827	15	HEI	Family breakfast frequency		✓								
Larson et al[161]	USA	CS	School	3699	14	FV, fried foods, SSBs, fat	Food preparation & shopping involvement, family meal frequency		✓								
Larson et al[162]	USA	CS	School	1687	20	FV, SSBs, fast food, energy intake, fat	Eating with others			✓							
Larson et al[163]	USA	CS	School	2540	14	Snacks	Involvement in at-home food preparation, family meal frequency, perceived encouragement to eat healthy food, parental restriction of high-calorie food, peer attitudes/behaviours and weight related behaviours		✓		✓						
Larson et al[164]	USA	L	School	1710	15	FV, SSBs, energy intake, fat, breakfast intake, meal skipping	Family meal frequency		✓								
Larson et al[165]	USA	L	School	1686	15	Fast food	Parental/peer support for healthy eating, living situation, meal preparation and shopping involvement, family meal frequency	✓	✓		✓						
Larson et al[166]	USA	L	School	1495	15	FV	Food preparation & shopping involvement, parental and peer support for healthy eating, family meal frequency		✓	✓	✓				✓		
Larson et al[167]	USA	L	School	1130	15	FV	Parents' /friends' /significant other's healthy eating attitudes, food preparation involvement		✓	✓			✓				✓
Larson et al[168]	USA	L	School	2052	25	FV, dairy, SSBs, energy intake, fat	Shared meals			✓							
Lau et al[169]	USA	L	University	532	21	Eating Behaviour Score*	Parent health behaviours, peer influence, living at home	✓		✓				✓			

Reference	Country	Study design	Setting	n participants	Middle-age	Outcome(s)	Determinant(s)	Determinant Sub-Category									
								Family structure	Family food culture	Social influence	Social support	Parental Resources & Risk Factors	Parental attitudes & beliefs	Parental behaviours	Parental feeding styles	Parenting style	Partner behaviours
Lazzeri et al[170]	Belgium, Canada, Croatia, Czech Republic, Denmark, Estonia, France, Finland, Germany, Greenland, Hungary, Ireland, Latvia, Lithuania, Macedonia, Netherlands, Norway, Poland, Portugal, Russian Federation, Slovenia, Spain, Sweden, Switzerland, UK, Ukraine, USA	CS	School	455391	13	Breakfast intake	Family structure (living with two parent, one parent or others)	✓									
Lebron et al[171]	USA	CS	School	280 dyads	13	FV, sweet snacks	Mealtime communication		✓								
Lebron et al[172]	USA	CS	School	280	13	FV, sweet snacks	Mealtime communication		✓								

Reference	Country	Study design	Setting	n participants	Middle-age	Outcome(s)	Determinant(s)	Determinant Sub-Category									
								Family structure	Family food culture	Social influence	Social support	Parental Resources & Risk Factors	Parental attitudes & beliefs	Parental behaviours	Parental feeding styles	Parenting style	Partner behaviours
LeCroy et al [173]	USA	CS	City/region	1214	14	Obesogenic dietary intake score*	Parental feeding style (controlling, pressuring, disciplinary, tracking, indulgent)								✓		
LeCroy et al[174]	USA	CS	City/region	740	12	HEI, FV, dairy, empty calories	Peer support for FV intake				✓						
Lenne et al[175]	USA	CS	Online	1717 dyads	14	FV, junk food	Parenting style (responsiveness, autonomy-granting, authoritative)									✓	
Levin et al[176]	UK	CS	School	26626	13	Breakfast intake	Family structure (single/dual/step parents or other)	✓									
Lien et al[177]	Norway	CS	City/region	613	14	FV	Parental monitoring, positive relationship with parents/peers, perceived parental evaluation of diet,			✓						✓	
Lipsky et al [178]	USA	L	School	566	16	HEI, Whole Plant Foods Density*, Empty Calories*	Family meal frequency		✓								
Lipsky et al [179]	USA	L	School	2785	16	FV, SSBs, snacks	Family meals		✓								
Loth et al[180]	USA	CS	School	2383 dyads	14	FV, SSBs, snacks	Parental modelling, overall food restriction							✓	✓		
Loucaides et al[181]	Republic of Cyprus	CS	School	1966	14	FV	Parental support, friends intake, family eating patterns		✓	✓	✓						
Lundeen et al[182]	USA	CS	Online	990 dyads	14	SSBs	Parental SSB intake, parental knowledge of conditions related to SSB intake					✓		✓			
Ma and Hample[183]	USA	CS	Online	1657	14	FV, SSBs, less healthful food	Parental control, modelling, co-decision making							✓		✓	
MacFarlane et al[184]	Australia	CS	School	1448 dyads	13	Snacks, extra food items	Parental concern about adolescent weight						✓				
Madani et al[185]	Saudi Arabia	CS	School	1408	15	SSBs	Parental intake, friends intake			✓				✓			
Mahjabin et al[186]	Bangladesh	CS	School	710	13	Diet diversity score*	Mother's nutritional knowledge					✓					
Maia et al[187]	Brazil	CS	School	10926	15	Data driven dietary pattern*	Meals with parents/guardians		✓								
Mario Arango-Paternina et al[188]	Colombia	CS	School	988	15	FV, fast food	Friendship network behaviours			✓							

Reference	Country	Study design	Setting	n participants	Middle-age	Outcome(s)	Determinant(s)	Determinant Sub-Category									
								Family structure	Family food culture	Social influence	Social support	Parental Resources & Risk Factors	Parental attitudes & beliefs	Parental behaviours	Parental feeding styles	Parenting style	Partner behaviours
Martens et al[189]	The Netherlands	CS	School	601	13	FV, snacks, breakfast intake	Subjective norm, social support, perceived behaviour of mother and father			✓	✓			✓			
Martins et al[190]	Brazil	CS	School	102072	15	FV, SSBs, snacks, UPFs, fast food, fried foods	Family meals		✓								
Mathur et al[191]	India	Qual	School	13 focus groups + 14 interviews	15	SSBs	Social norms			✓							
Matsumoto et al[192]	Japan	CS	School	288 dyads	13	Carbohydrates, fat, FV, SSBs, meat/fish/eggs, dairy, sweet and savoury snacks	Mother's nutrition knowledge level					✓					
Mehlig et al[193]	Belgium, Cyprus, Estonia, Germany, Hungary, Italy, Spain, Sweden	CS	City/region	1603	13	Fast food	Friends behaviours			✓							
Melbye et al[194]	Norway	CS	School	440	14	FV	Family structure, family functioning, family cohesion, family conflict, parental healthy eating guidance, parental positive encouragement for vegetable consumption, family meals	✓	✓		✓				✓		
Mellin et al[195]	USA	CS	School	9957	14	Breakfast intake, FV, snacks	Family connectedness, parental expectations, parental monitoring	✓									
Merten et al[196]	USA	L	School	7788	15	Breakfast intake	Parental presence in the morning							✓			
Michels et al[197]	Austria, Belgium, France, Germany,	CS	School	2081	14	DQI for Adolescents	Parental influence, friends influence (social norms, social support)			✓	✓						

Reference	Country	Study design	Setting	n participants	Middle-age	Outcome(s)	Determinant(s)	Determinant Sub-Category									
								Family structure	Family food culture	Social influence	Social support	Parental Resources & Risk Factors	Parental attitudes & beliefs	Parental behaviours	Parental feeding styles	Parenting style	Partner behaviours
	Greece, Hungary, Italy, Spain, Sweden																
Mieziene et al[198]	Lithuania	CS	City/region	1336	22	MEDAS	Family and social support, social trust, communication, collaboration, participation, distant communication, cohabitation	✓		✓	✓						
Mieziene et al[199]	Lithuania	CS	School	1863	16	MEDAS	Family support				✓						
Miller et al[200]	Australia	Qual	City/region	59	25	SSBs	Rules, modelling, social norms			✓				✓		✓	
Mirhadyan et al[201]	Iran	CS	School	341	16	Junk food	Household composition (living with parents, only father, only mother or others)	✓									
Mirkarimi et al[202]	Iran	CS	School	500	16	Fast food	Subjective norms			✓							
Mitri et al[203]	Lebanon	CS	School	798	15	KIDMED Index	Number of meals with family, autonomy, social support and peers,		✓		✓					✓	
Monge-Rojas et al[204]	Costa Rica	CS	City/region	813	15	FV, SSBs, UPFs, fast food	Family meals, parenting styles (authoritative, authoritarian)		✓							✓	
Moraes et al[205]	Brazil	CS	School	429	14	FV	Descriptive norms, injunctive norms			✓							
Morton et al[206]	Canada	CS	School	857	14	Adolescent Food Habits Checklist	Transformational parenting									✓	
Mwafi et al[207]	Jordan	CS	University	503	21	Fast food	Living with family or in a dorm, eating companion (friends, alone, family, colleagues)	✓		✓							
Nagahata et al[208]	Japan	CS	University	386	21	Breakfast intake	Living arrangement (home vs independently)	✓									
Nakade et al[209]	Japan	CS	University	1455		FV	Living with family or not, presence of others during breakfast	✓		✓							

Reference	Country	Study design	Setting	n participants	Middle-age	Outcome(s)	Determinant(s)	Determinant Sub-Category									
								Family structure	Family food culture	Social influence	Social support	Parental Resources & Risk Factors	Parental attitudes & beliefs	Parental behaviours	Parental feeding styles	Parenting style	Partner behaviours
Nasirzadeh et al[210]	Iran	CS	School	264	14	FV	Social support (friend support), observational learning			✓	✓						
Navarro-Gonzalez et al[211]	Spain	CS	University	318	21	KIDMED Index	Living arrangement	✓									
Nelson Laska et al[212]	USA	CS	School	1687	20	Meal skipping, snacking behaviour, fast food, FV, energy intake, fat, SSBs	Living arrangement (rented, with parents, on campus)	✓									
Neumark-Sztainer et al [213]	USA	CS	School	3957	14	FV	Parental/peer support for healthy eating, family meals frequency, parental presence at meals, priority of family meals		✓		✓					✓	
Neumark-Sztainer et al [214]	USA	CS	School	36284	15	FV	Family connectedness	✓									
Neumark-Sztainer et al[215]	USA	CS	School	4746	14	FV, snacks, SSBs, energy intake, carbohydrates, fat	Family meal frequency		✓								
Nickelson et al[216]	USA	CS	School	4049	13	SSBs	Parental limits on SSBs								✓		
Niermann et al[217]	Germany	CS	School	198 families	14	FV	Family health climate (the shared perceptions and cognitions concerning a healthy lifestyle within a family i.e. experience of daily family life, the evaluation of health-related topics and expectations with respect to typical values, behavior routines and interaction patterns within the family)		✓								
O'Leary et al[218]	UK	CS	Online	603 households	15	SSBs	Adult household members behaviours							✓			
Oliveira et al[219]	Brazil	CS	School	14653	14	FV, dairy, French fries, fried foods, snacks, SSBs	Lunch at home with parents							✓			
Orlowski et al[220]	USA	CS	Online	1354 dyads	14	FV	parents promote FV intake, parents ensure FV intake				✓				✓		

Reference	Country	Study design	Setting	n participants	Middle-age	Outcome(s)	Determinant(s)	Determinant Sub-Category									
								Family structure	Family food culture	Social influence	Social support	Parental Resources & Risk Factors	Parental attitudes & beliefs	Parental behaviours	Parental feeding styles	Parenting style	Partner behaviours
Overcash et al[221]	USA	CS	Online	1657	14	FV, SSBs, junk food, fast food	Family meal frequency and characteristics		✓								
Pandey et al[222]	Nepal	CS	Online	461	22	FV	Social influence regarding vegetables			✓							
Papadaki and Mavrikaki[223]	Greece	CS	School	525	14	KIDMED Index	Living with both parents	✓									
Parikka et al[224]	Finland	CS	Clinical	944	15	Meal skipping, breakfast intake	Family type (nuclear, reconstituted, single-parent, other)	✓									
Park[225]	South Korea	CS	University	2250	23	Nutrition adequacy ratio*, mean adequacy ratio*, Index of Nutritional Quality*, Eating Behaviour Score*	Residential status (living with family at home, student accommodation, independently)	✓									
Parks et al[226]	USA	CS	Online	805	14	FV	Parental preferences, role modelling, encouragement, rules, pressure to eat		✓		✓			✓	✓	✓	
Pearson et al[227]	UK	CS	School	328	14	FV, snacking behaviour, breakfast intake	Parenting style, parental status (single/dual), sibling status (none/one or more)	✓								✓	
Pearson et al[228]	Australia	L	School	1850	13	FV, snacks	Perceived support for healthy eating by best friend/family, modelling of healthy eating by best friend/mother			✓	✓			✓			
Pedersen et al[229]	Denmark	CS	School	3054	14	Breakfast intake	Family functioning (close relations to parents, quality of family communication, family support), family structure (traditional or other)	✓									
Pelletier et al[230]	USA	CS	University	1000	21	FV, fast food, SSBs	Family/friends/significant other social norms, perceived behaviours of friends, living arrangement	✓		✓							✓
Peltzer and Pengpid[231]	Bangladesh, Barbados, Cameroon, China,	CS	University	17789	20	FV	Living arrangement (on campus, with parents), social support	✓			✓						

Reference	Country	Study design	Setting	n participants	Middle-age	Outcome(s)	Determinant(s)	Determinant Sub-Category									
								Family structure	Family food culture	Social influence	Social support	Parental Resources & Risk Factors	Parental attitudes & beliefs	Parental behaviours	Parental feeding styles	Parenting style	Partner behaviours
	Colombia, Egypt, Grenada, India, Indonesia, Ivory Coast, Jamaica, Kyrgyzstan, Laos, Madagascar, Mauritius, Namibia, Nigeria, Pakistan, Philippines, Russian Federation, Singapore, South Africa, Thailand, Tunisia, Turkey, Venezuela																
Pendergast et al[232]	Australia	CS	City/region	986	24	Meal skipping	Living situation (family, alone, flatmates)		✓								
Pengpid and Peltzer [233]	Indonesia	CS	School	11124	14	FV, SSBs	Parental support, peer support				✓						
Pengpid and Peltzer [234]	Bangladesh, Barbados, Cameroon, Colombia,	CS	University	21958	20	Breakfast intake	Living with or away from parents, social support	✓			✓						

Reference	Country	Study design	Setting	n participants	Middle-age	Outcome(s)	Determinant(s)	Determinant Sub-Category									
								Family structure	Family food culture	Social influence	Social support	Parental Resources & Risk Factors	Parental attitudes & beliefs	Parental behaviours	Parental feeding styles	Parenting style	Partner behaviours
	Egypt, Grenada, India, Indonesia, Ivory Coast, Jamaica, Kyrgyzstan, Laos, Madagascar, Malaysia, Mauritius, Namibia, Myanmar, Nigeria, Pakistan, Philippines, Russian Federation, Singapore, South Africa, Thailand, Tunisia, Turkey, Venezuela, Vietnam																
Pengpid and Peltzer[235]	Laos	CS	School	3683	16	FV	Peer/parental support				✓						
Perkins et al [236]	USA	CS	School	5841	15	SSBs, FV	Perceived and actual behavioural norms			✓							
Perkins et al [237]	USA	CS	School	3831	14	SSBs	Perceived peer norms			✓							
Pokorski et al[238]	Poland	CS	University	657	23	DQI, Change in Food Intake*	Place of residence, parental monitoring	✓								✓	

Reference	Country	Study design	Setting	n participants	Middle-age	Outcome(s)	Determinant(s)	Determinant Sub-Category									
								Family structure	Family food culture	Social influence	Social support	Parental Resources & Risk Factors	Parental attitudes & beliefs	Parental behaviours	Parental feeding styles	Parenting style	Partner behaviours
Prichard et al[239]	Australia	CS	University	112	19	Energy dense foods (EB), FV	Mother's intake, closeness				✓			✓			
Qiu et al[240]	China	CS	School	3819	14	Junk food, SSBs	Perceived parental attitudes							✓	✓	✓	
Rafiroiu et al[241]	USA	CS	City/region	2021	15	Pyramid score*	Family and friends eating concerns			✓							
Rezaeipour et al[242]	Iran	CS	School	270	13	Eating Behaviour Score*	Parental lifestyle							✓			
Riebl et al[243]	USA	CS	City/region	100	14	SSBs	Subjective norm, parental SSB intake			✓				✓			
Riediger et al[244]	Canada	CS	City/region	18524	15	FV	Living arrangements (single or dual parent, or others)	✓									
Riggsbee et al[245]	USA	Qual	School	30	14	FV, fast food	Parental influences - positive/negative role modelling, access							✓			
Rimal[246]	USA	CS	City/region	824	15	Eating Behaviour Score*	Parental dietary behaviours							✓			
Robinson et al[247]	UK	CS	University	1056	21	SSBs, snacks	Perceived descriptive peer dietary norms			✓							
Roesler et al[248]	USA	CS	School	992	14	SSBs	Peer norms			✓							
Rosenrauch et al[249]	Australia	L	School	1785	13	Breakfast intake, meal skipping	Friends level of support for healthy eating, friend eat healthy foods with you, friends encourage you not to eat 'junk' food			✓	✓						
Rossow and Rise[250]	Norway	CS	City/region	272 families	18	Fat	Parental intake							✓			
Salvy et al[251]	USA	L	School	2144	16	Diet Quality Score*	Familism (needs of the family override those of the individual), peer social functioning	✓		✓							
San Román Mata et al[252]	Spain	CS	University	597	19	KIDMED Index	Living arrangement	✓									
Santaliestra-Pasías et al[253]	Austria, Belgium, France, Germany, Greece, Hungary,	CS	School	1703	14	FV, carbohydrates, sweet and savoury snacks, dairy, meat/fish/eggs, SSBs	Eating companion during main meal occasions			✓							

Reference	Country	Study design	Setting	n participants	Middle-age	Outcome(s)	Determinant(s)	Determinant Sub-Category									
								Family structure	Family food culture	Social influence	Social support	Parental Resources & Risk Factors	Parental attitudes & beliefs	Parental behaviours	Parental feeding styles	Parenting style	Partner behaviours
	Italy, Spain, Sweden																
Santomauro et al[254]	Italy	CS	City/region	1127	16	KIDMED Index	Source of dietary information, relationship with parents				✓	✓					
Sato et al[255]	Japan	CS	School	933	15	FV	Social support				✓						
Schnettler et al [256]	Chile	CS	City/region	300 triads	13	Adapted HEI	Satisfaction with family life	✓									
Schnettler et al[257]	Chile	CS	City/region	300 dyads	13	Spanish HEI and Dietary Variety Score	Maternal food-related practices (control of snacking behaviour, presence during eating, child involvement in food choice)		✓					✓	✓		
Schnettler et al[258]	Chile	CS	City/region	430 families	13	Adapted HEI	Father's and mother's modelling							✓			
Scully et al[259]	Australia	CS	School	8392	14	Fast food	Go to fast food outlets with family/friends, social norms		✓	✓							
Seedat and Pillay[260]	South Africa	CS	University	353	19	Breakfast intake	Person responsible for buying food/groceries (self, parents/family, housemates), place of residence	✓	✓								
Sexton-Dhamu et al[261]	Australia	CS	City/region	625	24	DGI	Eating alone, social support from family/friends, living situation (friends, family, alone), involvement in food preparation & shopping	✓	✓	✓	✓						
Shahanjarini et al[262]	Iran	CS	School	790	13	Snacks, junk food	Influential people (friends, parents, siblings, healthcare professionals, media)			✓							
Sharma et al[263]	Germany	CS	University	205	21	FV, dairy, meat/fish/eggs,	Residence (independent or dependent)	✓									
Silva et al[264]	Brazil	CS	School	101996	15	Breakfast intake	Meals with parents, living with parents/mother/father/alone	✓	✓								
Silva et al[265]	Brazil	CS	School	16324	15	UPFs	Living with mother/both parents, number of people in household, frequency of meals with parents	✓	✓								
Simões et al[266]	Brazil	CS	School	1232	13	Breakfast intake	Meal context, lunch and dinner with family		✓								

Reference	Country	Study design	Setting	n participants	Middle-age	Outcome(s)	Determinant(s)	Determinant Sub-Category									
								Family structure	Family food culture	Social influence	Social support	Parental Resources & Risk Factors	Parental attitudes & beliefs	Parental behaviours	Parental feeding styles	Parenting style	Partner behaviours
Small et al[267]	USA	L	University	746		FV	Parental communication									✓	
Smith et al[268]	USA	CS	Households	1522	13	FV	Parental provision of fruit and vegetables at meals or as a snack							✓			
Stephens et al [269]	Australia	CS	School	1014	13	FV	Family/friends support for healthy eating, mother/father/friends' role modelling, meal-time atmosphere, family meal-time rules		✓	✓	✓			✓	✓	✓	
Stephens et al [270]	Australia	L	School	521	13	FV	Family meal time rules, vegetables served at dinner									✓	
Stephens et al [271]	Australia	L	School	1938	13	High-energy foods and beverages	Evening meal atmosphere, meal-time rules								✓	✓	
Stewart et al[272]	USA	L	School	3744	15	FV, sweet snacks, breakfast intake, meal skipping, fast food	Father's involvement, household structure (single, dual, step parents)	✓									
Stok et al[273]	Poland, Portugal, Netherlands, UK	CS	School	2764	13	Diet Quality Score*	Peer encouragement for healthy/unhealthy eating				✓						
Sugiyama et al[274]	Japan	CS	School	3635	13	Breakfast intake	Living status (living with or without mother), eating status at breakfast (with parents/siblings/alone)	✓		✓							
Tabak et al[275]	Poland	CS	School	605	13	FV, meal skipping	Family meal frequency		✓								
Tak et al[276]	The Netherlands	CS	School	1361	14	SSBs	Parental modelling, parental rules, parental norm			✓				✓		✓	
Tang et al[277]	Australia	CS	City/region	1003	21	ARFS	Social desirability score, social approval score				✓						
Tassitano et al[278]	Brazil	CS	University	717	20	FV	Peer and parental support				✓						

Reference	Country	Study design	Setting	n participants	Middle-age	Outcome(s)	Determinant(s)	Determinant Sub-Category									
								Family structure	Family food culture	Social influence	Social support	Parental Resources & Risk Factors	Parental attitudes & beliefs	Parental behaviours	Parental feeding styles	Parenting style	Partner behaviours
Thomson et al[279]	USA	CS	Online	1657 dyads	14	Junk food	Parenting practices (negative emotions, restriction, monitoring, modelling, child involvement)		✓					✓	✓	✓	
Toumpakari et al[280]	UK	CS	Households	666	14	Non-core food groups intake score*, energy intake	Eating companion (alone, parents/carers, parents & siblings, family & friends, friends), eating context (home, friend's/relative's house, school, eateries, on the go, activity/other places, work)			✓							
Vågstrand [281]	Sweden	CS	Birth cohort	474	16	SSBs	Mother's intake of SSBs, fruit juice and total energy							✓			
Vågstrand et al [282]	Sweden	CS	Birth cohort	471 dyads	16	Energy intake, fat, carbohydrates, snacks, FV, fast food, SSBs, dairy	Mother's intake							✓			
van den Bogerd et al[283]	The Netherlands	CS	University	717	22	FV	Housing situation (shared, alone, parents)	✓									
van den Broek et al[284]	The Netherlands	L	School	2051	13	Snacks	Mother's snacking							✓			
van der Horst et al[285]	The Netherlands	CS	School	383	13	SSBs	Modelling from parents/friends, social norms, social pressure, parenting style (strictness, involvement)			✓				✓		✓	
van der Horst et al[286]	The Netherlands	CS	School	1293	14	SSBs	Parents and friends modelling, parental norm			✓				✓			
Van Hulst et al[287]	Canada	CS	School	377	16	FV	Household chaos, dual-parent household	✓									
Van Lippevelde et al[288]	Belgium	CS	School	867	14	SSBs, snacks	Health-promoting/reducing parenting practices (scales), family type (traditional or other)	✓						✓			
Vanhelst et al[289]	Austria, Belgium, France, Germany,	CS	School	2943	14	DQI	Father, mother, brother, sister and best friend diet engagement and encouragement			✓							

Reference	Country	Study design	Setting	n participants	Middle-age	Outcome(s)	Determinant(s)	Determinant Sub-Category									
								Family structure	Family food culture	Social influence	Social support	Parental Resources & Risk Factors	Parental attitudes & beliefs	Parental behaviours	Parental feeding styles	Parenting style	Partner behaviours
	Greece, Hungary, Italy, Spain, Sweden																
Vaterlaus et al[290]	USA	Qual	University	34	20	Food choices (qualitative)	Connection between food and social media - increased food choices (e.g. recipes), shared pictures from peers of food, and as a distraction when making food choices.			✓							
Vejrup et al[291]	Norway	L	School	1504	12	FV	Parental intake							✓			
Verstraeten et al[292]	Ecuador	CS	School	784	13	SSBs	Parenting practices (rules, role modelling, permissiveness)							✓		✓	
Verzeletti et al[293]	Italy and Belgium	CS	School	14407	13	FV	Dinner with family, family rules		✓							✓	
Verzeletti et al[294]	Italy and Belgium	CS	School	14407	13	SSBs	Daily breakfast or evening meal with parents, restriction/obligation rules		✓							✓	
Videon et al[295]	USA	CS	School	18177	15	Breakfast intake, FV, dairy	Parental presence, family meals, autonomy with food decision making		✓					✓		✓	
Vogel et al[296]	UK	Qual	Households	74	15	Energy drinks (SSBs)	Peer and parental influence			✓				✓			
Walton et al[297]	USA	CS	City/region	2728	19	FV, SSBs, takeaway foods, fast food	Family structure (dual or single parent), family functioning, family dinner frequency	✓	✓								
Wang et al[298]	Japan	CS	City/region	2308		FV	Social support				✓						
Watts et al [299]	Canada	CS	City/region	173	13	Canadian HEI	Peer modelling, eating out with friends			✓							
Watts et al [300]	USA	CS	School	2793		SSBs	Parental encouragement for healthy eating, parental pressure to eat, parental restriction of unhealthy food, parental intake, frequency of family meals,		✓	✓	✓			✓	✓		

Reference	Country	Study design	Setting	n participants	Middle-age	Outcome(s)	Determinant(s)	Determinant Sub-Category									
								Family structure	Family food culture	Social influence	Social support	Parental Resources & Risk Factors	Parental attitudes & beliefs	Parental behaviours	Parental feeding styles	Parenting style	Partner behaviours
							parental modelling, peer intake, peer attitudes about healthy eating										
Watts et al [301]	USA	CS	School	2491	14	FV	Parental modelling, parental encouragement for healthy eating, parent communication, family meal frequency		✓		✓			✓			
Watts et al [302]	Canada	CS	City/region	165 dyads	13	FV, fat, SSBs, sweet and savoury snacks	Parent intake							✓			
Welsh et al [303]	USA	CS	City/region	75	14	FV, snacks, SSBs	Family cohesion, family meals	✓	✓								
Wills [304]	UK	Qual	School	31	20	Meal skipping, snacking behaviour	Living arrangement, adult surveillance, eating environment (presence of parents vs friends), peer influences, parental modelling	✓		✓				✓		✓	
Winpenny et al [305]	USA	L	School	2902	14	Fast food	Leaving the parental home, cohabitation		✓								
Winpenny et al [306]	Norway	L	City/region	1100	22	FV, snacks, SSBs	Living situation (parents, friends/others, spouse/partner, alone)		✓								
Woodruff et al [307]	Canada	CS	School	1293	12	Canadian HEI	Eating companion, family dinners		✓	✓							
Woodward et al [308]	Australia	CS	School	2082	13	Snacks, SSBs, meat/fish/eggs, FV, dairy	Parental and friends' intake			✓				✓			
Wouters et al [309]	The Netherlands	CS	School	1330	14	Snacks, SSBs	Friendship group consumption			✓							
Wrobleski et al [310]	USA	CS	City/region	216 dyads	13	HEI	Parental monitoring of diet, parental beliefs about nutrition, peer eating behaviour			✓			✓			✓	
Wu et al [311]	USA	CS	School	274	15	EHI	Social support for healthy eating				✓						
Xiang et al [312]	Malaysia	CS	University	422	19	Snacks	Perceived parental control of snacking								✓		
Xu et al [313]	USA	CS	Online	1890	14	Fast food, FV, sweet snacks	Parental intake, family meals		✓					✓			
Yasugi et al [314]	Japan	CS	University	290		Breakfast intake	Living arrangement	✓									

Interpersonal determinants of diet quality and eating behaviours in people aged 13-30 years: a systematic scoping review.

Reference	Country	Study design	Setting	n participants	Middle-age	Outcome(s)	Determinant(s)	Determinant Sub-Category									
								Family structure	Family food culture	Social influence	Social support	Parental Resources & Risk Factors	Parental attitudes & beliefs	Parental behaviours	Parental feeding styles	Parenting style	Partner behaviours
Yazdi Feyzabadi et al[315]	Iran	CS	School	1320	14	Snacking behaviour, snacks	Family structure (dual or single parent), social norms, perceived parental control	✓									
Young et al[316]	USA	CS	School	366	14	FV	Authoritative parenting, parent control, parent modelling, parent support, FV availability				✓			✓		✓	
Young and Fors[317]	USA	CS	School	3155	15	FV, breakfast intake	Parental monitoring, family communication	✓								✓	
Yuan et al[318]	China	CS	University	419	25	FV, SSBs, sweet and savoury snacks, eating outside of the home	Peer effects of dorm-mates			✓							
Yuhas et al[319]	USA	CS	Online	1560	14	SSBs	Social norms, perceived parenting practices			✓				✓	✓	✓	
Zaborskis et al[320]	Albania, Austria, Belgium, Bulgaria, Canada, Croatia, Czech Republic, Denmark, England, Estonia, Finland, France, Germany, Greece, Greenland, Hungary, Iceland,	CS	School	192755	13	FV, snacks, SSBs	Family structure (intact vs not)	✓									

Reference	Country	Study design	Setting	n participants	Middle-age	Outcome(s)	Determinant(s)	Determinant Sub-Category									
								Family structure	Family food culture	Social influence	Social support	Parental Resources & Risk Factors	Parental attitudes & beliefs	Parental behaviours	Parental feeding styles	Parenting style	Partner behaviours
	Ireland, Israel, Italy, Latvia, Lithuania, Luxembourg, Malta, Netherlands, North Macedonia, Norway, Poland, Portugal, Republic of Moldova, Romania, Russian Federation, Scotland, Slovakia, Slovenia, Spain, Sweden, Switzerland, Ukraine, and Wales.																
Zaborskis et al[321]	Lithuania	CS	School	17189	13	FV, snacks, SSBs, fast food	Family structure (intact, non-intact)	✓									
Zahra et al[322]	UK	CS	School	10645	14	Meal skipping, junk food	Authoritarian, authoritative, permissive, uninvolved, neutral									✓	
Zarychta et al[323]	Poland	L	School	100	16	Diet Quality Score*	Parental health behaviours, parental verbal pressure							✓	✓		

Interpersonal determinants of diet quality and eating behaviours in people aged 13-30 years: a systematic scoping review.

Reference	Country	Study design	Setting	n participants	Middle-age	Outcome(s)	Determinant(s)	Determinant Sub-Category									
								Family structure	Family food culture	Social influence	Social support	Parental Resources & Risk Factors	Parental attitudes & beliefs	Parental behaviours	Parental feeding styles	Parenting style	Partner behaviours
Zeinivanmoghadam et al[324]	Iran	CS	School	332	13	FV	Social support				✓						
Zhang et al[325]	China	CS	School	1090 dyads	13	Carbohydrates	Parental intake, knowledge, attitude and guidance behaviour					✓		✓			
Ziaei et al[326]	Iran	CS	School	1517	16	FV	Parental support, parental connectivity, peer support				✓						
Zolghadr et al[327]	Iran	CS	School	330	14	Diet Quality Score*	Social support				✓						
Zuercher et al[328]	USA	CS	Households	836	14	FV, dairy, meat	Head of household intake							✓			
Zytnick et al[329]	USA	CS	Households	815 dyads	14	Sports drinks (SSBs)	Caregiver attitudes toward sports drinks						✓				

Table S4: Countries studied across all included papers, by study design

Country	Cross-Sectional (n)	Longitudinal (n)	Qualitative (n)	Other (n)	Total (n)
Albania	1	0	0	0	1
Australia	14	8	4	1	27
Austria	5	0	0	0	5
Bangladesh	4	0	0	0	4
Barbados	2	0	0	0	2
Belgium	13	0	1	0	14
Brazil	15	0	0	0	15
Bulgaria	1	0	0	0	1
Cameroon	2	0	0	0	2
Canada	8	2	0	0	10
Chile	3	0	0	0	3
China	5	1	0	0	6
Colombia	3	0	0	0	3
Costa Rica	1	0	0	0	1
Croatia	2	0	0	0	2
Cyprus	2	0	0	0	2
Czech Republic	2	0	0	0	2
Denmark	4	0	0	0	4
Dominica	1	0	0	0	1
Ecuador	1	0	0	0	1
Egypt	3	0	0	0	3
Estonia	3	0	0	0	3
Fiji	1	0	0	0	1
Finland	4	0	0	0	4
France	7	0	0	0	7
Germany	11	1	0	0	12
Ghana	3	0	1	0	4
Greece	7	0	0	0	7
Greenland	2	0	0	0	2
Grenada	3	0	0	0	3
Hungary	7	0	0	0	7
Iceland	1	0	0	0	1
India	5	0	2	0	7
Indonesia	6	0	0	0	6
Iran	13	0	0	0	13
Ireland	2	0	0	0	2
Israel	1	0	0	0	1
Italy	11	1	0	0	12
Ivory Coast	2	0	0	0	2
Jamaica	3	0	0	0	3
Japan	11	0	0	1	12
Jordan	3	0	0	0	3
Kyrgyzstan	2	0	0	0	2
Laos	3	0	0	0	3
Latvia	2	0	0	0	2
Lebanon	1	1	0	0	2
Lithuania	5	0	0	0	5
Luxembourg	1	0	0	0	1
North Macedonia	2	0	0	0	2
Madagascar	2	0	0	0	2
Malawi	1	0	0	0	1
Malaysia	8	0	0	0	8
Maldives	1	0	0	0	1

Malta	1	0	0	0	1
Mauritius	2	0	0	0	2
Mexico	1	0	0	0	1
Mongolia	1	0	0	0	1
Myanmar	1	0	0	0	1
Namibia	2	0	0	0	2
Nepal	2	0	0	0	2
Nigeria	3	0	0	0	3
Norway	10	2	0	0	12
Pakistan	2	0	0	0	2
Peru	1	0	0	0	1
Philippines	3	1	0	0	4
Poland	5	1	0	0	6
Portugal	3	0	0	0	3
Republic of Moldova	1	0	0	0	1
Romania	1	0	0	0	1
Russian Federation	4	0	0	0	4
Saudi Arabia	8	0	0	0	8
Singapore	2	0	0	0	2
Slovakia	1	0	0	0	1
Slovenia	2	0	0	0	2
South Africa	3	0	0	0	3
South Korea	5	0	0	0	5
Spain	12	0	0	0	12
Sweden	10	0	0	0	10
Switzerland	2	0	0	0	2
Syria	1	0	0	0	1
Thailand	4	0	0	0	4
The Netherlands	13	1	0	1	15
Tunisia	2	0	0	0	2
Turkey	3	0	0	0	3
Uganda	1	0	0	0	1
United Kingdom	10	2	3	0	15
Ukraine	2	0	0	0	2
United Arab Emirates	1	0	0	0	1
United States of America	77	23	5	0	105
Venezuela	2	0	0	0	2
Vietnam	1	0	0	0	1

References

1. Abar CC, Clark G, Koban K. The Long-Term Impact of Family Routines and Parental Knowledge on Alcohol Use and Health Behaviors: Results from a 14 Year Follow-Up. *J Child Fam Stud*. 2017;26(9):2495-2504. doi:10.1007/s10826-017-0752-2
2. Abizari AR, Ali Z. Dietary patterns and associated factors of schooling Ghanaian adolescents. *J Health Popul Nutr*. 2019;38(1):5. doi:10.1186/s41043-019-0162-8
3. Agustina R, Rianda D, Setiawan EA. Relationships of Child-, Parents-, and Environment-Associated Determinants with Diet Quality, Physical Activity, and Smoking Habits Among Indonesian Urban Adolescents. *Food Nutr Bull*. 2022;43(1):44-55. doi:10.1177/03795721211046145
4. Al-Naggar RA, Bobryshev Y V., Mohd Noor NAB. Lifestyle Practice among Malaysian University Students. *Asian Pacific Journal of Cancer Prevention*. 2013;14(3):1895-1903. doi:10.7314/APJCP.2013.14.3.1895
5. Al-Otaibi HH. The Pattern of Fruit and Vegetable Consumption among Saudi University Students. *Glob J Health Sci*. 2013;6(2):155-162. doi:10.5539/gjhs.v6n2p155
6. Ali RA, Abdel Razeq NM, Al-Kloub MI, Alzoubi FA. Predictors of breakfast skipping among 14 to 16 years old adolescents in Jordan: The influential role of mothers. *Int J Nurs Pract*. 2019;25(6):e12778. doi:10.1111/ijn.12778
7. Alimoradi F, Jandaghi P, Khodabakhshi A, Javadi M, Zehni Moghadam SAH. Breakfast and fast food eating behavior in relation to socio-demographic differences among school adolescents in Sanandaj Province, Iran. *Electron Physician*. 2017;9(6):4510-4515. doi:10.19082/4510
8. Almutairi KM, Alonazi WB, Vinluan JM, et al. Health promoting lifestyle of university students in Saudi Arabia: a cross-sectional assessment. *BMC Public Health*. 2018;18(1):1093. doi:10.1186/s12889-018-5999-z
9. Alolabi H, Alchallah MO, Mohsen F, Marrawi M, Alourfi Z. Social and psychosocial factors affecting eating habits among students studying at the Syrian Private University: A questionnaire based cross-sectional study. *Heliyon*. 2022;8(5):e09451. doi:10.1016/j.heliyon.2022.e09451
10. Alsunni AA, Badar A. Fruit and vegetable consumption and its determinants among Saudi university students. *J Taibah Univ Med Sci*. 2015;10(2):201-207. doi:10.1016/j.jtumed.2014.11.003
11. Aluqmany R, Mansoor R, Saad U, Abdullah R, Ahamd A. Consumption of energy drinks among female secondary school students, Almadinah Almunawwarah, Kingdom of Saudi Arabia, 2011. *J Taibah Univ Med Sci*. 2013;8(1):60-65. doi:10.1016/j.jtumed.2013.01.009
12. Alzahrani SH, Saeedi AA, Baamer MK, Shalabi AF, Alzahrani AM. Eating Habits Among Medical Students at King Abdulaziz University, Jeddah, Saudi Arabia. *Int J Gen Med*. 2020;13:77-88. doi:10.2147/IJGM.S246296
13. Ambrosini GL, Oddy WH, Robinson M, et al. Adolescent dietary patterns are associated with lifestyle and family psycho-social factors. *Public Health Nutr*. 2009;12(10):1807-1815. doi:10.1017/S1368980008004618
14. Anastacio-Landa F, López-Hernández E, Argüelles-Nava VG, Dominguez-Lara SA, Rosas-Santiago FJ, Campos-Uscanga Y. Parental Hostility and its relationship with sugar-sweetened and alcoholic beverage intake among Mexican adolescents. *Revista Mexicana de Trastornos Alimentarios/Mexican Journal of Eating Disorders*. 2022;12(2):105-118. doi:10.22201/fesi.20071523e.2022.2.578
15. Appannah G, Murray K, Trapp G, Dymock M, Oddy WH, Ambrosini GL. Dietary pattern trajectories across adolescence and early adulthood and their associations with childhood and parental factors. *Am J Clin Nutr*. 2021;113(1):36-46. doi:10.1093/ajcn/nqaa281
16. Arabi-Mianrood H, Shahhosseini Z, Tabaghdehi MH. The association between body image, emotional health, relationships, and unhealthy dietary behaviors among medical sciences students: A structural equation modeling analysis. *Neuropsychopharmacol Rep*. 2022;42(4):485-491. doi:10.1002/npr2.12291
17. Arcan C, Neumark-Sztainer D, Hannan P, van den Berg P, Story M, Larson N. Parental eating behaviours, home food environment and adolescent intakes of fruits, vegetables and dairy foods: longitudinal findings from Project EAT. *Public Health Nutr*. 2007;10(11):1257-1265. doi:10.1017/S1368980007687151
18. Arora M, Nazar GP, Gupta VK, Perry CL, Reddy KS, Stigler MH. Association of breakfast intake with obesity, dietary and physical activity behavior among urban school-aged adolescents in Delhi, India: results of a cross-sectional study. *BMC Public Health*. 2012;12(1):881. doi:10.1186/1471-2458-12-881
19. Arroyo A, Segrin C, Harwood J, Bonito JA. Co-Rumination of Fat Talk and Weight Control Practices: An Application of Confirmation Theory. *Health Commun*. 2017;32(4):438-450. doi:10.1080/10410236.2016.1140263

20. Ashton LM, Hutchesson MJ, Rollo ME, Morgan PJ, Thompson DI, Collins CE. Young adult males' motivators and perceived barriers towards eating healthily and being active: a qualitative study. *International Journal of Behavioral Nutrition and Physical Activity*. 2015;12(1):93. doi:10.1186/s12966-015-0257-6
21. Azeredo CM, de Rezende LFM, Canella DS, et al. Dietary intake of Brazilian adolescents. *Public Health Nutr*. 2015;18(7):1215-1224. doi:10.1017/S1368980014001463
22. Backman DR, Haddad EH, Lee JW, Johnston PK, Hodgkin GE. Psychosocial Predictors of Healthful Dietary Behavior in Adolescents. *J Nutr Educ Behav*. 2002;34(4):184-193. doi:10.1016/S1499-4046(06)60092-4
23. Badrasawi M, Anabtawi O, Al-Zain Y. Breakfast characteristics, perception, and reasons of skipping among 8th and 9th-grade students at governmental schools, Jenin governance, West Bank. *BMC Nutr*. 2021;7(1):42. doi:10.1186/s40795-021-00451-1
24. Bagherniya M, Sharma M, Mostafavi F, Keshavarz SA. Application of Social Cognitive Theory in Predicting Childhood Obesity Prevention Behaviors in Overweight and Obese Iranian Adolescents. *Int Q Community Health Educ*. 2015;35(2):133-147. doi:10.1177/0272684X15569487
25. Baig T, Ganesan GS, Ibrahim H, Yousuf W, Mahfoud ZR. The association of parental involvement with adolescents' well-being in Oman: evidence from the 2015 Global School Health Survey. *BMC Psychol*. 2021;9(1):175. doi:10.1186/s40359-021-00677-5
26. Bailey C, Garg V, Kapoor D, Wasser H, Prabhakaran D, Jaacks LM. Food Choice Drivers in the Context of the Nutrition Transition in Delhi, India. *J Nutr Educ Behav*. 2018;50(7):675-686. doi:10.1016/j.jneb.2018.03.013
27. Baker CW, Little TD, Brownell KD. Predicting adolescent eating and activity behaviors: the role of social norms and personal agency. *Health Psychol*. 2003;22(2):189-198. http://www.ncbi.nlm.nih.gov/pubmed/12683739
28. Baldwin JN, Haslam RL, Clarke E, et al. Eating Behaviors and Diet Quality: A National Survey of Australian Young Adults. *J Nutr Educ Behav*. 2022;54(5):397-405. doi:10.1016/j.jneb.2021.12.001
29. Baltaci A, Alvarez de Davila S, Reyes Peralta AO, et al. Adolescent-Reported Latino Fathers' Food Parenting Practices and Family Meal Frequency Are Associated with Better Adolescent Dietary Intake. *Int J Environ Res Public Health*. 2021;18(15):8226. doi:10.3390/ijerph18158226
30. Barco Leme AC, Tucunduva Philippi S. Home food availability, parents'/caregivers' support, and family meals influence on dietary servings of low-income urban adolescent girls from Brazil. *Nutrire*. 2017;42(1):30. doi:10.1186/s41110-017-0053-y
31. Bau AM, Krull S, Ernert A, Babitsch B. Eating behaviour and its association with social living conditions and weight status among adolescent girls: results of the cross-sectional Berlin School Children's Cohort study. *Public Health Nutr*. 2011;14(10):1759-1767. doi:10.1017/S1368980011000541
32. Bauer KW, Neumark-Sztainer D, Fulkerson JA, Hannan PJ, Story M. Familial correlates of adolescent girls' physical activity, television use, dietary intake, weight, and body composition. *International Journal of Behavioral Nutrition and Physical Activity*. 2011;8(1):25. doi:10.1186/1479-5868-8-25
33. Bauer KW, Larson NI, Nelson MC, Story M, Neumark-Sztainer D. Socio-environmental, personal and behavioural predictors of fast-food intake among adolescents. *Public Health Nutr*. 2009;12(10):1767-1774. doi:10.1017/S1368980008004394
34. Befort C, Kaur H, Nollen N, et al. Fruit, Vegetable, and Fat Intake among Non-Hispanic Black and Non-Hispanic White Adolescents: Associations with Home Availability and Food Consumption Settings. *J Am Diet Assoc*. 2006;106(3):367-373. doi:10.1016/j.jada.2005.12.001
35. Benetou V, Kanellopoulou A, Kanavou E, et al. Diet-Related Behaviors and Diet Quality among School-Aged Adolescents Living in Greece. *Nutrients*. 2020;12(12):3804. doi:10.3390/nu12123804
36. Berge JM, Meyer C, MacLehose RF, Crichlow R, Neumark-Sztainer D. All in the family: Correlations between parents' and adolescent siblings' weight and weight-related behaviors. *Obesity*. 2015;23(4):833-839. doi:10.1002/oby.21036
37. Berge JM, Hazzard VM, Larson N, Hahn SL, Emery RL, Neumark-Sztainer D. Are there protective associations between family/shared meal routines during COVID-19 and dietary health and emotional well-being in diverse young adults? *Prev Med Rep*. 2021;24:101575. doi:10.1016/j.pmedr.2021.101575
38. Berge JM, MacLehose RF, Larson N, Laska M, Neumark-Sztainer D. Family Food Preparation and Its Effects on Adolescent Dietary Quality and Eating Patterns. *Journal of Adolescent Health*. 2016;59(5):530-536. doi:10.1016/j.jadohealth.2016.06.007
39. Berge JM, MacLehose R, Eisenberg ME, Laska MN, Neumark-Sztainer D. How significant is the 'significant other'? Associations between significant others' health behaviors and attitudes and young

adults' health outcomes. *International Journal of Behavioral Nutrition and Physical Activity*. 2012;9(1):35. doi:10.1186/1479-5868-9-35
40. Berge JM, Meyer C, MacLehose RF, Eisenberg ME, Neumark-Sztainer D. Nonresident parental influence on adolescent weight and weight-related behaviors: similar or different from resident parental influence? *International Journal of Behavioral Nutrition and Physical Activity*. 2014;11(1):131. doi:10.1186/s12966-014-0131-y
41. Berge JM, MacLehose RF, Loth KA, Eisenberg ME, Fulkerson JA, Neumark-Sztainer D. Parent-adolescent conversations about eating, physical activity and weight: prevalence across sociodemographic characteristics and associations with adolescent weight and weight-related behaviors. *J Behav Med*. 2015;38(1):122-135. doi:10.1007/s10865-014-9584-3
42. Berge JM, Jin SW, Hannan P, Neumark-Sztainer D. Structural and Interpersonal Characteristics of Family Meals: Associations with Adolescent Body Mass Index and Dietary Patterns. *J Acad Nutr Diet*. 2013;113(6):816-822. doi:10.1016/j.jand.2013.02.004
43. Berge JM, Wall M, Loth K, Neumark-Sztainer D. Parenting Style as a Predictor of Adolescent Weight and Weight-Related Behaviors. *Journal of Adolescent Health*. 2010;46(4):331-338. doi:10.1016/j.jadohealth.2009.08.004
44. Bibi Nabihah AH, Hanis Mastura Y, Suzana S, Zahara Abdul M. Fruit consumption and associated determinants in a sample of young urban Malaysian adults. *Malays J Nutr*. 2018;24(4):567-574.
45. Billon S, Lluch A, Guéguen R, Berthier A, Siest G, Herbeth B. Family resemblance in breakfast energy intake: the Stanislas Family Study. *Eur J Clin Nutr*. 2002;56(10):1011-1019. doi:10.1038/sj.ejcn.1601440
46. Bohara SS, Thapa K, Bhatt LD, Dhami SS, Wagle S. Determinants of Junk Food Consumption Among Adolescents in Pokhara Valley, Nepal. *Front Nutr*. 2021;8:644650. doi:10.3389/fnut.2021.644650
47. Borlu A, Aykut M, Celik N, Gun I, Timur A, Karaca S. Fruit and vegetable consumption of last grade medical students and related factors. *Progress in Nutrition*. 2019;21(1):86-92. doi:10.23751/pn.v21i1.6384
48. Bourdeaudhuij I De, Oost P Van. Personal and family determinants of dietary behaviour in adolescents and their parents. *Psychol Health*. 2000;15(6):751-770. doi:10.1080/08870440008405579
49. Boylan JM, Cundiff JM, Jakubowski KP, Pardini DA, Matthews KA. Pathways Linking Childhood SES and Adult Health Behaviors and Psychological Resources in Black and White Men. *Annals of Behavioral Medicine*. 2018;52(12):1023-1035. doi:10.1093/abm/kay006
50. Brindal E, Wilson C, Mohr P, Wittert G. Eating in groups: Do multiple social influences affect intake in a fast-food restaurant? *J Health Psychol*. 2015;20(5):483-489. doi:10.1177/1359105315576607
51. Bruening M, MacLehose R, Eisenberg ME, Nanney MS, Story M, Neumark-Sztainer D. Associations Between Sugar-Sweetened Beverage Consumption and Fast-Food Restaurant Frequency Among Adolescents and Their Friends. *J Nutr Educ Behav*. 2014;46(4):277-285. doi:10.1016/j.jneb.2014.02.009
52. Burgess-Champoux TL, Larson N, Neumark-Sztainer D, Hannan PJ, Story M. Are Family Meal Patterns Associated with Overall Diet Quality during the Transition from Early to Middle Adolescence? *J Nutr Educ Behav*. 2009;41(2):79-86. doi:10.1016/j.jneb.2008.03.113
53. Burke V, Beilin L, Dunbar D. Family lifestyle and parental body mass index as predictors of body mass index in Australian children: a longitudinal study. *Int J Obes*. 2001;25(2):147-157. doi:10.1038/sj.ijo.0801538
54. Campbell KJ, Crawford DA, Salmon J, Carver A, Garnett SP, Baur LA. Associations Between the Home Food Environment and Obesity-promoting Eating Behaviors in Adolescence*. *Obesity*. 2007;15(3):719-730. doi:10.1038/oby.2007.553
55. Chan V, Wellard-Cole L, Davies A, et al. The association of social and food preparation location context with the quality of meals and snacks consumed by young adults: findings from the MYMeals wearable camera study. *Eur J Nutr*. 2022;61(7):3407-3422. doi:10.1007/s00394-022-02891-2
56. Chan V, Davies A, Wellard-Cole L, Allman-Farinelli M. The energy density of meals and snacks consumed by young Australian adults (18–30 years old) are influenced by preparation location but not screen use nor social interactions: findings from the MYMeals wearable camera study. *J Nutr Sci*. 2022;11:e76. doi:10.1017/jns.2022.76
57. Chang YJ, Peng CY, Lan YC. Consumption of Energy Drinks among Undergraduate Students in Taiwan: Related Factors and Associations with Substance Use. *Int J Environ Res Public Health*. 2017;14(9):954. doi:10.3390/ijerph14090954
58. Chansukree P, Rungjindarat N. Social Cognitive Determinants of Healthy Eating Behaviors in Late Adolescents: A Gender Perspective. *J Nutr Educ Behav*. 2017;49(3):204-210.e1. doi:10.1016/j.jneb.2016.10.019

59. Chaves OC, Velasquez-Melendez G, Costa DA da S, Andrade RG de, Caiaffa WT. Cooccurrence of obesogenic risk factors in Brazilian adolescents: the role of sociodemographic characteristics and parental presence. *Cad Saude Publica*. 2021;37(3):e00013120. doi:10.1590/0102-311x00013120
60. Cheikh Ismail L, Osaili TM, Mohamad MN, et al. Psychosocial factors affecting dietary habits of university students: A cross-sectional study. *Heliyon*. 2022;8(6):e09768. doi:10.1016/j.heliyon.2022.e09768
61. Chen L, Zhu H, Gutin B, Dong Y. Race, Gender, Family Structure, Socioeconomic Status, Dietary Patterns, and Cardiovascular Health in Adolescents. *Curr Dev Nutr*. 2019;3(11):nzz117. doi:10.1093/cdn/nzz117
62. Choi EJ, Choi MK. Eating Out Status according to Skipping and Type of Breakfast among Male High School Students in Incheon. *Korean Journal of Community Nutrition*. 2020;25(2):102. doi:10.5720/kjcn.2020.25.2.102
63. Christofaro DGD, Tebar WR, Mota J, et al. Gender Analyses of Brazilian Parental Eating and Activity With Their Adolescents' Eating Habits. *J Nutr Educ Behav*. 2020;52(5):503-511. doi:10.1016/j.jneb.2019.09.015
64. Chung SJ, Ersig AL, McCarthy AM. Parent, school, and peer factors related to U.S. adolescents' diet and exercise. *Journal for Specialists in Pediatric Nursing*. 2018;23(4):e12227. doi:10.1111/jspn.12227
65. Cluskey M, Grobe D. College Weight Gain and Behavior Transitions: Male and Female Differences. *J Am Diet Assoc*. 2009;109(2):325-329. doi:10.1016/j.jada.2008.10.045
66. Cornelius T, Desrosiers A, Kershaw T. Spread of health behaviors in young couples: How relationship power shapes relational influence. *Soc Sci Med*. 2016;165:46-55. doi:10.1016/j.socscimed.2016.07.030
67. Cusatis DC, Shannon BM. Influences on adolescent eating behavior. *Journal of Adolescent Health*. 1996;18(1):27-34. doi:10.1016/1054-139X(95)00125-C
68. Cutler GJ, Flood A, Hannan P, Neumark-Sztainer D. Multiple Sociodemographic and Socioenvironmental Characteristics Are Correlated with Major Patterns of Dietary Intake in Adolescents. *J Am Diet Assoc*. 2011;111(2):230-240. doi:10.1016/j.jada.2010.10.052
69. Darfour-Oduro SA, Andrade JE, Grigsby-Toussaint DS. Do Fruit and Vegetable Policies, Socio-Environmental Factors, and Physical Activity Influence Fruit and Vegetable Intake Among Adolescents? *Journal of Adolescent Health*. 2020;66(2):172-180. doi:10.1016/j.jadohealth.2019.07.016
70. de Bruijn GJ, Kremers SPJ, de Vries H, van Mechelen W, Brug J. Associations of social-environmental and individual-level factors with adolescent soft drink consumption: results from the SMILE study. *Health Educ Res*. 2006;22(2):227-237. doi:10.1093/her/cyl066
71. de la Haye K, Robins G, Mohr P, Wilson C. Adolescents' Intake of Junk Food: Processes and Mechanisms Driving Consumption Similarities Among Friends. *Journal of Research on Adolescence*. 2013;23(3):524-536. doi:10.1111/jora.12045
72. de Souza MR, Andrade AC de S, Froelich M, Muraro AP, Rodrigues PRM. Association of household composition with dietary patterns among adolescents in Brazil. *British Journal of Nutrition*. Published online January 24, 2023:1-7. doi:10.1017/S000711452300020X
73. DeJong CS, van Lenthe FJ, van der Horst K, Oenema A. Environmental and cognitive correlates of adolescent breakfast consumption. *Prev Med (Baltim)*. 2009;48(4):372-377. doi:10.1016/j.ypmed.2009.02.009
74. Delbosq S, Velasco V, Vercesi C, Vecchio LP. Adolescents' Nutrition: The Role of Health Literacy, Family and Socio-Demographic Variables. *Int J Environ Res Public Health*. 2022;19(23):15719. doi:10.3390/ijerph192315719
75. Deliens T, Clarys P, De Bourdeaudhuij I, Deforche B. Correlates of University Students' Soft and Energy Drink Consumption According to Gender and Residency. *Nutrients*. 2015;7(8):6550-6566. doi:10.3390/nu7085298
76. Deliens T, Verhoeven H, De Bourdeaudhuij I, et al. Factors associated with fruit and vegetable and total fat intake in university students: A cross-sectional explanatory study. *Nutrition & Dietetics*. 2018;75(2):151-158. doi:10.1111/1747-0080.12399
77. Deliens T, Clarys P, De Bourdeaudhuij I, Deforche B. Determinants of eating behaviour in university students: a qualitative study using focus group discussions. *BMC Public Health*. 2014;14(1):53. doi:10.1186/1471-2458-14-53
78. Demissie Z, Eaton DK, Lowry R, et al. The Association of Meal Practices and other Dietary Correlates with Dietary Intake among High School Students in the United States, 2010. *American Journal of Health Promotion*. 2015;29(6):e203-e213. doi:10.4278/ajhp.131211-QUAN-632

79. Dewi NU, Khomsan A, Dwiriani CM, et al. Factors Associated with Diet Quality among Adolescents in a Post-Disaster Area: A Cross-Sectional Study in Indonesia. *Nutrients*. 2023;15(5):1101. doi:10.3390/nu15051101
80. Dickens E, Ogden J. The role of parental control and modelling in predicting a child's diet and relationship with food after they leave home. A prospective study. *Appetite*. 2014;76:23-29. doi:10.1016/j.appet.2014.01.013
81. Doegah PT, Acquah E. Promoting healthy lifestyles among nurse trainees: Perceptions on enablers and barriers to dietary and physical activity behaviours. Steinborn MB, ed. *PLoS One*. 2022;17(6):e0270353. doi:10.1371/journal.pone.0270353
82. Dong F, Howard AG, Herring AH, et al. Parent–child associations for changes in diet, screen time, and physical activity across two decades in modernizing China: China Health and Nutrition Survey 1991–2009. *International Journal of Behavioral Nutrition and Physical Activity*. 2016;13(1):118. doi:10.1186/s12966-016-0445-z
83. Dwyer LA, Bolger N, Laurenceau JP, et al. Autonomous Motivation and Fruit/Vegetable Intake in Parent–Adolescent Dyads. *Am J Prev Med*. 2017;52(6):863-871. doi:10.1016/j.amepre.2017.01.011
84. El Tantawi M, Bakhurji E, Al-Ansari A, Al-Khalifa KS, AlSubaie A. Influences of parents, close friends and classmates on four co-existing oral health practices in Saudi male teenagers. *Acta Odontol Scand*. 2017;75(2):137-143. doi:10.1080/00016357.2016.1269192
85. Elstgeest LEM, Mishra GD, Dobson AJ. Transitions in Living Arrangements Are Associated with Changes in Dietary Patterns in Young Women. *J Nutr*. 2012;142(8):1561-1567. doi:10.3945/jn.112.158188
86. Endalifer ML, Andargie G, Mohammed B, Endalifer BL. Factors associated with dietary diversity among adolescents in Woldia, Northeast Ethiopia. *BMC Nutr*. 2021;7:27. doi:10.1186/s40795-021-00430-6
87. Eto K, Nakanishi A, Takemi Y. Associations between Family Dinner Frequency and Voluntary Mealtime Communication with Dietary Attitudes, Dietary Behaviors, and Quality of Life: A Cross-sectional and Longitudinal Study of 5th and 8th Grade Students. *The Japanese Journal of Nutrition and Dietetics*. 2014;72(3):113-125. doi:10.5264/eiyogakuzashi.72.113
88. Feldman S, Eisenberg ME, Neumark-Sztainer D, Story M. Associations between Watching TV during Family Meals and Dietary Intake Among Adolescents. *J Nutr Educ Behav*. 2007;39(5):257-263. doi:10.1016/j.jneb.2007.04.181
89. Ferrara M, Langiano E, Falese L, Diotaiuti P, Cortis C, De Vito E. Changes in Physical Activity Levels and Eating Behaviours during the COVID-19 Pandemic: Sociodemographic Analysis in University Students. *Int J Environ Res Public Health*. 2022;19(9):5550. doi:10.3390/ijerph19095550
90. Feunekes GIJ, de Graaf C, Meyboom S, van Staveren WA. Food Choice and Fat Intake of Adolescents and Adults: Associations of Intakes within Social Networks. *Prev Med (Baltim)*. 1998;27(5):645-656. doi:10.1006/pmed.1998.0341
91. Figueroa R, Kalyoncu ZB, Saltzman JA, Davison KK. Autonomous motivation, sugar-sweetened beverage consumption and healthy beverage intake in US families: differences between mother–adolescent and father–adolescent dyads. *Public Health Nutr*. 2019;22(06):1010-1018. doi:10.1017/S136898001800383X
92. Fink SK, Racine EF, Mueffelmann RE, Dean MN, Herman-Smith R. Family Meals and Diet Quality Among Children and Adolescents in North Carolina. *J Nutr Educ Behav*. 2014;46(5):418-422. doi:10.1016/j.jneb.2014.05.004
93. Fismen AS, Smith ORF, Samdal O, Helleve A, Haug E. Associations between family structure and adolescents' food habits. *Public Health Nutr*. 2022;25(3):702-709. doi:10.1017/S1368980020004334
94. Fleary SA, Ettienne R. The relationship between food parenting practices, parental diet and their adolescents' diet. *Appetite*. 2019;135:79-85. doi:10.1016/j.appet.2019.01.008
95. Fondevila-Gascón JF, Berbel-Giménez G, Vidal-Portés E, Hurtado-Galarza K. Ultra-Processed Foods in University Students: Implementing Nutri-Score to Make Healthy Choices. *Healthcare*. 2022;10(6):984. doi:10.3390/healthcare10060984
96. Forleo MB, Tamburro M, Mastronard L, Giaccio V, Ripabelli G. Food consumption and eating habits: a segmentation of university students from Central-South Italy. *New Medit*. 2017;16(4):56-65.
97. Franko DL, Thompson D, Bauserman R, Affenito SG, Striegel-Moore RH. What's love got to do with it? Family cohesion and healthy eating behaviors in adolescent girls. *International Journal of Eating Disorders*. 2008;41(4):360-367. doi:10.1002/eat.20517
98. French S, Story M, Neumark-Sztainer D, Fulkerson J, Hannan P. Fast food restaurant use among adolescents: associations with nutrient intake, food choices and behavioral and psychosocial variables. *Int J Obes*. 2001;25(12):1823-1833. doi:10.1038/sj.ijo.0801820

99. Fu J, Liang F, Wang Y, et al. Modeling Parental Influence on Food Consumption among Chinese Adolescents through Self-Efficacy: A Path Analysis. *Nutrients*. 2021;13(12):4454. doi:10.3390/nu13124454
100. Galimov A, Hanewinkel R, Hansen J, Unger JB, Sussman S, Morgenstern M. Energy drink consumption among German adolescents: Prevalence, correlates, and predictors of initiation. *Appetite*. 2019;139:172-179. doi:10.1016/j.appet.2019.04.016
101. Gan WY, Mohamed SF, Law LS. Unhealthy Lifestyle Associated with Higher Intake of Sugar-Sweetened Beverages among Malaysian School-Aged Adolescents. *Int J Environ Res Public Health*. 2019;16(15):2785. doi:10.3390/ijerph16152785
102. Ganasegeran K, Al-Dubai SA, Qureshi AM, Al-abed A abed A, AM R, Aljunid SM. Social and psychological factors affecting eating habits among university students in a Malaysian medical school: a cross-sectional study. *Nutr J*. 2012;11(1):48. doi:10.1186/1475-2891-11-48
103. Garrido-Fernández A, García-Padilla FM, Sánchez-Ramos JL, Gómez-Salgado J, Travé-González GH, Sosa-Cordobés E. Food Consumed by High School Students during the School Day. *Nutrients*. 2020;12(2):485. doi:10.3390/nu12020485
104. Gebremariam MK, Henjum S, Terragni L, Elin Torheim L. Correlates of fruit, vegetable, soft drink, and snack intake among adolescents: the ESSENS study. *Food Nutr Res*. 2016;60:32512. doi:10.3402/fnr.v60.32512
105. Gebremariam MK, Henjum S, Hurum E, Utne J, Terragni L, Torheim LE. Mediators of the association between parental education and breakfast consumption among adolescents : the ESSENS study. *BMC Pediatr*. 2017;17(1):61. doi:10.1186/s12887-017-0811-2
106. Gebremariam MK, Lien N, Torheim LE, et al. Perceived rules and accessibility: measurement and mediating role in the association between parental education and vegetable and soft drink intake. *Nutr J*. 2015;15(1):76. doi:10.1186/s12937-016-0196-3
107. Gevers DWM, van Assema P, Sleddens EFC, de Vries NK, Kremers SPJ. Associations between general parenting, restrictive snacking rules, and adolescent's snack intake. The roles of fathers and mothers and interparental congruence. *Appetite*. 2015;87:184-191. doi:10.1016/j.appet.2014.12.220
108. Gillman MW, Rifas-Shiman S, Frazier AL, et al. Family Dinner and Diet Quality Among Older Children and Adolescents. *Arch Fam Med*. 2000;9(3):235-240. doi:10.1001/archfami.9.3.235
109. Glozah FN, Pevalin DJ. Perceived social support and parental education as determinants of adolescents' physical activity and eating behaviour: a cross-sectional survey. *Int J Adolesc Med Health*. 2015;27(3):253-259. doi:10.1515/ijamh-2014-0019
110. Goñi Murillo C, Vilches C, Ancizu Irure E, et al. The factors related to eating behaviors in a juvenile urban population. *Aten Primaria*. 1999;23(1):32-37. http://www.ncbi.nlm.nih.gov/pubmed/10079558
111. Graham DJ, Pelletier JE, Neumark-Sztainer D, Lust K, Laska MN. Perceived Social-Ecological Factors Associated with Fruit and Vegetable Purchasing, Preparation, and Consumption among Young Adults. *J Acad Nutr Diet*. 2013;113(10):1366-1374. doi:10.1016/j.jand.2013.06.348
112. Granner ML, Sargent RG, Calderon KS, Hussey JR, Evans AE, Watkins KW. Factors of Fruit and Vegetable Intake by Race, Gender, and Age among Young Adolescents. *J Nutr Educ Behav*. 2004;36(4):173-180. doi:10.1016/S1499-4046(06)60231-5
113. Grunseit AC, Cook AS, Conti J, Gwizd M, Allman-Farinelli M. "Doing a good thing for myself": a qualitative study of young adults' strategies for reducing takeaway food consumption. *BMC Public Health*. 2019;19(1):525. doi:10.1186/s12889-019-6731-3
114. Gulati S, Misra A, Colles SL, et al. Dietary Intakes and Familial Correlates of Overweight/Obesity: A Four-Cities Study in India. *Ann Nutr Metab*. 2013;62(4):279-290. doi:10.1159/000346554
115. Gummeson L, Jonsson I, Conner M. Predicting intentions and behaviour of Swedish 10–16-year-olds at breakfast. *Food Qual Prefer*. 1997;8(4):297-306. doi:10.1016/S0950-3293(97)00013-X
116. Haddad MR, Sarti FM, Nishijima M. Association between selected individual and environmental characteristics in relation to health behavior of Brazilian adolescents. *Eating and Weight Disorders - Studies on Anorexia, Bulimia and Obesity*. 2021;26(1):331-343. doi:10.1007/s40519-020-00856-0
117. Haddad MR, Sarti FM. Sociodemographic determinants of health behaviors among Brazilian adolescents: Trends in physical activity and food consumption, 2009–2015. *Appetite*. 2020;144:104454. doi:10.1016/j.appet.2019.104454
118. Haidar A, Ranjit N, Saxton D, Hoelscher DM. Perceived Parental and Peer Social Support Is Associated With Healthier Diets in Adolescents. *J Nutr Educ Behav*. 2019;51(1):23-31. doi:10.1016/j.jneb.2018.10.003

119. Haines J, Rifas-Shiman SL, Horton NJ, et al. Family functioning and quality of parent-adolescent relationship: cross-sectional associations with adolescent weight-related behaviors and weight status. *International Journal of Behavioral Nutrition and Physical Activity*. 2016;13(1):68. doi:10.1186/s12966-016-0393-7
120. Haley M, Aucoin D, Rae J. A comparative study of food habits: influence of age, sex, and selected family characteristics. II. *Canadian Journal of Public Health*. 1977;68(4):301-306. Accessed August 9, 2023. https://www.jstor.org/stable/41987600
121. Hallström L, Vereecken CA, Labayen I, et al. Breakfast habits among European adolescents and their association with sociodemographic factors: the HELENA (Healthy Lifestyle in Europe by Nutrition in Adolescence) study. *Public Health Nutr*. 2012;15(10):1879-1889. doi:10.1017/S1368980012000341
122. Hamade H, Moriyasu A, Kushida O. Associations between Cooking at Home and Nutrient and Food Group Intake among Female University Students: A Cross-Sectional Analysis on Living Arrangements. *Nutrients*. 2023;15(4):1029. doi:10.3390/nu15041029
123. Hanson NI, Neumark-Sztainer D, Eisenberg ME, Story M, Wall M. Associations between parental report of the home food environment and adolescent intakes of fruits, vegetables and dairy foods. *Public Health Nutr*. 2005;8(1):77-85. doi:10.1079/PHN2005661
124. Harker D, Sharma B, Harker M, Reinhard K. Leaving home: Food choice behavior of young German adults. *J Bus Res*. 2010;63(2):111-115. doi:10.1016/j.jbusres.2009.02.007
125. Hasan T, Sultana M. Actitud de los adolescentes hacia los refrescos y factores asociados a su consumo. *Enfermería Clínica (English Edition)*. 2022;32(6):431-439. doi:10.1016/j.enfcle.2021.12.006
126. Hassan MR, Ghazi HF, Umar NS, et al. Knowledge, Attitude and Practice of Healthy Eating and Associated Factors among University Students in Selangor, Malaysia. *Pakistan Journal of Nutrition*. 2015;14(12):892-897. doi:10.3923/pjn.2015.892.897
127. Hattersley L, Irwin M, King L, Allman-Farinelli M. Determinants and patterns of soft drink consumption in young adults: a qualitative analysis. *Public Health Nutr*. 2009;12(10):1816-1822. doi:10.1017/S136898000800462X
128. Haugland SH, Coombes L, Stea TH. Associations between parenting and substance use, meal pattern and food choices: A cross-sectional survey of 13,269 Norwegian adolescents. *Prev Med Rep*. 2019;14:100862. doi:10.1016/j.pmedr.2019.100862
129. Hawkins LK, Farrow C, Thomas JM. Do perceived norms of social media users' eating habits and preferences predict our own food consumption and BMI? *Appetite*. 2020;149((Hawkins, Farrow, Thomas) Department of Psychology, Aston University, Birmingham B4 7ET, United Kingdom):104611. doi:http://dx.doi.org/10.1016/j.appet.2020.104611
130. Hayek J, Tueni M, Schneider F, de Vries H. Parenting style as longitudinal predictor of adolescents' health behaviors in Lebanon. *Health Educ Res*. 2021;36(1):100-115. doi:10.1093/her/cyaa045
131. Hong S, Bae HC, Kim HS, Park EC. Variation in Meal-skipping Rates of Korean Adolescents According to Socio-economic Status: Results of the Korea Youth Risk Behavior Web-based Survey. *Journal of Preventive Medicine & Public Health*. 2014;47(3):158-168. doi:10.3961/jpmph.2014.47.3.158
132. Hormenu T. Dietary intake and its associated factors among in-school adolescents in Ghana. Mohammadi S, ed. *PLoS One*. 2022;17(5):e0268319. doi:10.1371/journal.pone.0268319
133. Huamancayo-Espíritu A, Pérez-Cárdenas L. Prevalence and factors associated with the low consumption of fruits and vegetables in students of the professional career of human medicine of a Peruvian university. *Revista de la Facultad de Medicina Humana*. 2020;20(1):123-129. doi:10.25176/RFMH.v20i1.2660
134. Ilesanmi OS, Ilesanmi FF, Ijarotimi IT. Determinants of fruit consumption among in-school adolescents in Ibadan, South West Nigeria. *Eur J Nutr Food Saf*. 2014;4(2):100-109.
135. Imoisili OE, Park S, Lundeen EA, Yaroch AL, Blanck HM. Daily Adolescent Sugar-Sweetened Beverage Intake Is Associated With Select Adolescent, Not Parent, Attitudes About Limiting Sugary Drink and Junk Food Intake. *American Journal of Health Promotion*. 2020;34(1):76-82. doi:10.1177/0890117119868382
136. Isabirye N, Bukenya JN, Nakafeero M, Ssekamatte T, Guwatudde D, Fawzi W. Dietary diversity and associated factors among adolescents in eastern Uganda: a cross-sectional study. *BMC Public Health*. 2020;20(1):534. doi:10.1186/s12889-020-08669-7
137. Johnson CC, Shi R, Farris R, Webber LS, Nicklas TA. Social support and self-efficacy for the consumption of fruit and vegetables by adolescents: "Gimme 5"—A fresh nutrition concept for students. *Ecol Food Nutr*. 2000;39(5):357-374. doi:10.1080/03670244.2000.9991624

138. Jones A, Robinson E. The Longitudinal Associations between Perceived Descriptive Peer Norms and Eating and Drinking Behavior: An Initial Examination in Young Adults. *Front Psychol*. 2017;8. doi:10.3389/fpsyg.2017.00002
139. Jorgensen A, Pedersen TP, Meilstrup CR, Rasmussen M. The influence of family structure on breakfast habits among adolescents. *Dan Med Bull*. 2011;58(5):A4262.
140. Karimi-Shahanjarini A, Rashidian A, Majdzadeh R, Omidvar N, Tabatabai M, Shojaeezadeh D. Parental Control and Junk-Food Consumption: A Mediating and Moderating Effect Analysis1. *J Appl Soc Psychol*. 2012;42(5):1241-1265. doi:10.1111/j.1559-1816.2011.00885.x
141. Kassem NO, Lee JW. Understanding Soft Drink Consumption Among Male Adolescents Using the Theory of Planned Behavior. *J Behav Med*. 2004;27(3):273-296. doi:10.1023/B:JOBM.0000028499.29501.8f
142. Kawasaki Y, Akamatsu R, Fujiwara Y, et al. Association of healthy eating literacy and resident status with energy, nutrients, and food consumption among lean and normal-weight female university students. *Clin Nutr ESPEN*. 2022;51:419-423. doi:10.1016/j.clnesp.2022.07.007
143. Kelles A, Adair L. Offspring consume a more obesogenic diet than mothers in response to changing socioeconomic status and urbanization in Cebu, Philippines. *International Journal of Behavioral Nutrition and Physical Activity*. 2009;6(1):47. doi:10.1186/1479-5868-6-47
144. Keski-Rahkonen A, Kaprio J, Rissanen A, Virkkunen M, Rose RJ. Breakfast skipping and health-compromising behaviors in adolescents and adults. *Eur J Clin Nutr*. 2003;57(7):842-853. doi:10.1038/sj.ejcn.1601618
145. Khalaf A, Westergren A, Berggren V, Ekblom Ö, Al-Hazzaa HM. Prevalence and association of female weight status and dietary habits with sociodemographic factors: a cross-sectional study in Saudi Arabia. *Public Health Nutr*. 2015;18(5):784-796. doi:10.1017/S1368980014001797
146. Kilanowski JF. Influences on Healthy-Eating Decision Making in Latino Adolescent Children of Migrant and Seasonal Agricultural Workers. *Journal of Pediatric Health Care*. 2016;30(3):224-230. doi:10.1016/j.pedhc.2015.07.004
147. Kim MJ, McIntosh WA, Anding J, Kubena KS, Reed DB, Moon GS. Perceived parenting behaviours predict young adolescents' nutritional intake and body fatness. *Matern Child Nutr*. 2008;4(4):287-303. doi:10.1111/j.1740-8709.2008.00142.x
148. Kim SJ, Bu SY, Choi MK. Preference and the Frequency of Processed Food Intake according to the Type of Residence of College Students in Korea. *Korean Journal of Community Nutrition*. 2015;20(3):188. doi:10.5720/kjcn.2015.20.3.188
149. Klaiber P, Whillans A V., Chen FS. Long-Term Health Implications of Students' Friendship Formation during the Transition to University. *Appl Psychol Health Well Being*. 2018;10(2):290-308. doi:10.1111/aphw.12131
150. Kobayashi S, Asakura K, Suga H, Sasaki S. Cohabitational effect of grandparents on dietary intake among young Japanese women and their mothers living together. A multicenter cross-sectional study. *Appetite*. 2015;91:287-297. doi:10.1016/j.appet.2015.04.059
151. Kobayashi S, Asakura K, Suga H, Sasaki S. Living status and frequency of eating out-of-home foods in relation to nutritional adequacy in 4,017 Japanese female dietetic students aged 18–20 years: A multicenter cross-sectional study. *J Epidemiol*. 2017;27(6):287-293. doi:10.1016/j.je.2016.07.002
152. Koehn S, Gillison F, Standage M, Bailey J. Life transitions and relevance of healthy living in late adolescence. *J Health Psychol*. 2016;21(6):1085-1095. doi:10.1177/1359105314546340
153. Kremers SPJ, Brug J, de Vries H, Engels RCME. Parenting style and adolescent fruit consumption. *Appetite*. 2003;41(1):43-50. doi:10.1016/S0195-6663(03)00038-2
154. Kumari A, Jain M. Association of Dietary Behaviour with Demographic and Socio-economic Factors and Physical Activity among Rural Adolescents: A Cross-Sectional Study. *Indian J Public Health Res Dev*. 2022;13(2):13-22. doi:10.37506/ijphrd.v13i2.17887
155. Kwon HS, Kang SH, Park YS, Kang JG, Park EC. Association of Household Type and Fast-Food Consumption in Korean Adolescents. *Nutrients*. 2022;14(15):3024. doi:10.3390/nu14153024
156. LaCaille LJ, Dauner KN, Krambeer RJ, Pedersen J. Psychosocial and Environmental Determinants of Eating Behaviors, Physical Activity, and Weight Change Among College Students: A Qualitative Analysis. *Journal of American College Health*. 2011;59(6):531-538. doi:10.1080/07448481.2010.523855
157. Lahmann PH, Williams GM, Najman JM, Mamun AA. Mother–adult offspring resemblance in dietary intake: a community-based cohort study in Australia. *Am J Clin Nutr*. 2017;105(1):185-193. doi:10.3945/ajcn.116.137539

158. Lally P, Bartle N, Wardle J. Social norms and diet in adolescents. *Appetite*. 2011;57(3):623-627. doi:10.1016/j.appet.2011.07.015
159. Larson N, MacLehose R, Fulkerson JA, Berge JM, Story M, Neumark-Sztainer D. Eating Breakfast and Dinner Together as a Family: Associations with Sociodemographic Characteristics and Implications for Diet Quality and Weight Status. *J Acad Nutr Diet*. 2013;113(12):1601-1609. doi:10.1016/j.jand.2013.08.011
160. Larson N, Wang Q, Berge JM, Shanafelt A, Nanney MS. Eating breakfast together as a family: mealtime experiences and associations with dietary intake among adolescents in rural Minnesota, USA. *Public Health Nutr*. 2016;19(9):1565-1574. doi:10.1017/S1368980016000379
161. Larson NI, Story M, Eisenberg ME, Neumark-Sztainer D. Food Preparation and Purchasing Roles among Adolescents: Associations with Sociodemographic Characteristics and Diet Quality. *J Am Diet Assoc*. 2006;106(2):211-218. doi:10.1016/j.jada.2005.10.029
162. Larson NI, Nelson MC, Neumark-Sztainer D, Story M, Hannan PJ. Making Time for Meals: Meal Structure and Associations with Dietary Intake in Young Adults. *J Am Diet Assoc*. 2009;109(1):72-79. doi:10.1016/j.jada.2008.10.017
163. Larson N, Miller JM, Eisenberg ME, Watts AW, Story M, Neumark-Sztainer D. Multicontextual correlates of energy-dense, nutrient-poor snack food consumption by adolescents. *Appetite*. 2017;112:23-34. doi:10.1016/j.appet.2017.01.008
164. Larson NI, Neumark-Sztainer D, Hannan PJ, Story M. Family Meals during Adolescence Are Associated with Higher Diet Quality and Healthful Meal Patterns during Young Adulthood. *J Am Diet Assoc*. 2007;107(9):1502-1510. doi:10.1016/j.jada.2007.06.012
165. Larson NI, Neumark-Sztainer DR, Story MT, Wall MM, Harnack LJ, Eisenberg ME. Fast Food Intake: Longitudinal Trends during the Transition to Young Adulthood and Correlates of Intake. *Journal of Adolescent Health*. 2008;43(1):79-86. doi:10.1016/j.jadohealth.2007.12.005
166. Larson NI, Neumark-Sztainer DR, Harnack LJ, Wall MM, Story MT, Eisenberg ME. Fruit and Vegetable Intake Correlates During the Transition to Young Adulthood. *Am J Prev Med*. 2008;35(1):33-37.e3. doi:10.1016/j.amepre.2008.03.019
167. Larson N, Laska MN, Story M, Neumark-Sztainer D. Predictors of Fruit and Vegetable Intake in Young Adulthood. *J Acad Nutr Diet*. 2012;112(8):1216-1222. doi:10.1016/j.jand.2012.03.035
168. Larson N, Fulkerson J, Story M, Neumark-Sztainer D. Shared meals among young adults are associated with better diet quality and predicted by family meal patterns during adolescence. *Public Health Nutr*. 2013;16(5):883-893. doi:10.1017/S1368980012003539
169. Lau RR, Quadrel MJ, Hartman KA. Development and Change of Young Adults' Preventive Health Beliefs and Behavior: Influence from Parents and Peers. *J Health Soc Behav*. 1990;31(3):240. doi:10.2307/2136890
170. Lazzeri G, Ahluwalia N, Niclasen B, et al. Trends from 2002 to 2010 in Daily Breakfast Consumption and its Socio-Demographic Correlates in Adolescents across 31 Countries Participating in the HBSC Study. Rosenfeld CS, ed. *PLoS One*. 2016;11(3):e0151052. doi:10.1371/journal.pone.0151052
171. Lebron CN, Agosto Y, Lee TK, et al. Family Mealtime Communication in Single- and Dual-Headed Households Among Hispanic Adolescents With Overweight and Obesity. *J Nutr Educ Behav*. 2020;52(9):840-849. doi:10.1016/j.jneb.2020.03.003
172. Lebron CN, Lee TK, Prado G, St. George SM, Pantin H, Messiah SE. Psychometric properties of an abbreviated Childhood Family Mealtime Questionnaire among overweight and obese Hispanic adolescents. *Appetite*. 2019;140:169-179. doi:10.1016/j.appet.2019.05.011
173. LeCroy MN, Siega-Riz AM, Albrecht SS, et al. Association of food parenting practice patterns with obesogenic dietary intake in Hispanic/Latino youth: Results from the Hispanic Community Children's Health Study/Study of Latino Youth (SOL Youth). *Appetite*. 2019;140:277-287. doi:10.1016/j.appet.2019.05.006
174. LeCroy MN, Mossavar-Rahmani Y, Xue X, et al. Diet quality comparisons in Hispanic/Latino siblings: Results from the Hispanic Community Children's Health Study/Study of Latino Youth (SOL Youth). *Appetite*. 2022;169:105809. doi:10.1016/j.appet.2021.105809
175. Lenne RL, Joyal-Desmarais K, Jones RE, et al. Parenting styles moderate how parent and adolescent beliefs shape each other's eating and physical activity: Dyadic evidence from a cross-sectional, U.S. National Survey. *J Exp Soc Psychol*. 2019;81:76-84. doi:10.1016/j.jesp.2018.06.003
176. Levin KA, Kirby J, Currie C. Family structure and breakfast consumption of 11-15 year old boys and girls in Scotland, 1994-2010: a repeated cross-sectional study. *BMC Public Health*. 2012;12(1):228. doi:10.1186/1471-2458-12-228

177. Lien N, Jacobs DR, Klepp KI. Exploring predictors of eating behaviour among adolescents by gender and socio-economic status. *Public Health Nutr*. 2002;5(5):671-681. doi:10.1079/PHN2002334
178. Lipsky LM, Nansel TR, Haynie DL, et al. Diet quality of US adolescents during the transition to adulthood: changes and predictors ,. *Am J Clin Nutr*. 2017;105(6):1424-1432. doi:10.3945/ajcn.116.150029
179. Lipsky LM, Haynie DL, Liu D, et al. Trajectories of eating behaviors in a nationally representative cohort of U.S. adolescents during the transition to young adulthood. *International Journal of Behavioral Nutrition and Physical Activity*. 2015;12(1):138. doi:10.1186/s12966-015-0298-x
180. Loth KA, MacLehose RF, Larson N, Berge JM, Neumark-Sztainer D. Food availability, modeling and restriction: How are these different aspects of the family eating environment related to adolescent dietary intake? *Appetite*. 2016;96:80-86. doi:10.1016/j.appet.2015.08.026
181. Loucaides CA, Jago R, Theophanous M. Social, attitudinal and behavioural correlates of fruit and vegetable consumption among Cypriot adolescents. *Public Health Nutr*. 2011;14(12):2139-2147. doi:10.1017/S1368980011000784
182. Lundeen EA, Park S, Onufrak S, Cunningham S, Blanck HM. Adolescent Sugar-Sweetened Beverage Intake is Associated With Parent Intake, Not Knowledge of Health Risks. *American Journal of Health Promotion*. 2018;32(8):1661-1670. doi:10.1177/0890117118763008
183. Ma Z, Hample D. Modeling Parental Influence on Teenagers' Food Consumption: An Analysis Using the Family Life, Activity, Sun, Health, and Eating (FLASHE) Survey. *J Nutr Educ Behav*. 2018;50(10):1005-1014. doi:10.1016/j.jneb.2018.07.005
184. MacFarlane A, Crawford D, Worsley A. Associations Between Parental Concern for Adolescent Weight and the Home Food Environment and Dietary Intake. *J Nutr Educ Behav*. 2010;42(3):152-160. doi:10.1016/j.jneb.2008.11.004
185. Madani KA, Jambi HA, Sadiq BM Bin, Malky SA, Salah MK. Factors associated with soft drink consumption in school-aged girls in Saudi Arabia. *International Journal of Food Safety, Nutrition and Public Health*. 2008;1(2):150. doi:10.1504/IJFSNPH.2008.023015
186. Mahjabin T, Nowar A, Islam MH, Jubayer A. Effect of Mother's Nutritional Knowledge and Hygiene Practices on School-Going Adolescents Living in Dhaka City of Bangladesh. *Indian J Community Med*. 2022;47(3):391-395. doi:10.4103/ijcm.ijcm_1240_21
187. Maia EG, Silva LES da, Santos MAS, Barufaldi LA, Silva SU da, Claro RM. Dietary patterns, sociodemographic and behavioral characteristics among Brazilian adolescents. *Revista Brasileira de Epidemiologia*. 2018;21(suppl 1):e180009. doi:10.1590/1980-549720180009.supl.1
188. Mario Arango-Paternina C, Lema-Gómez L, Eusse-López C, et al. Composition of Friendship Networks and Health-Related Behaviors in Adolescents. *Merrill Palmer Q*. 2022;68(1):1-24. doi:10.1353/mpq.2022.0001
189. Martens M, Assema P van, Brug J. Why do adolescents eat what they eat? Personal and social environmental predictors of fruit, snack and breakfast consumption among 12– 14-year-old Dutch students. *Public Health Nutr*. 2005;8(8):1258-1265. doi:10.1079/PHN2005828
190. Martins BG, Ricardo CZ, Machado PP, Rauber F, Azeredo CM, Levy RB. Eating meals with parents is associated with better quality of diet for Brazilian adolescents. *Cad Saude Publica*. 2019;35(7):e00153918. doi:10.1590/0102-311x00153918
191. Mathur M, George M, Nagrath D, Watt R. Exploring the perceptions, attitude and experiences of adolescents, their parents and teachers towards sugar sweetened beverages consumption in the National Capital Region of Delhi. *Indian Journal of Dental Research*. 2021;32(1):39. doi:10.4103/ijdr.IJDR_607_20
192. Matsumoto M, Hatamoto Y, Masumoto A, Sakamoto A, Ikemoto S. Mothers' Nutrition Knowledge Is Unlikely to Be Related to Adolescents' Habitual Nutrient Intake Inadequacy in Japan: A Cross-Sectional Study of Japanese Junior High School Students. *Nutrients*. 2020;12(9):2801. doi:10.3390/nu12092801
193. Mehlig K, Holmberg C, Bogl LH, et al. Weight Status and BMI-Related Traits in Adolescent Friendship Groups and Role of Sociodemographic Factors: The European IDEFICS/I.Family Cohort. *Obes Facts*. 2021;14(1):121-130. doi:10.1159/000512356
194. Melbye EL, Hausken-Sutter SE, Lien N, Bjelland M. Adolescent vegetable consumption: the role of socioemotional family characteristics. *Public Health Nutr*. 2021;24(17):5710-5719. doi:10.1017/S1368980021001658
195. Mellin AE, Neumark-Sztainer D, Story M, Ireland M, Resnick MD. Unhealthy behaviors and psychosocial difficulties among overweight adolescents: the potential impact of familial factors. *Journal of Adolescent Health*. 2002;31(2):145-153. doi:10.1016/S1054-139X(01)00396-2

196. Merten MJ, Williams AL, Shriver LH. Breakfast Consumption in Adolescence and Young Adulthood: Parental Presence, Community Context, and Obesity. *J Am Diet Assoc*. 2009;109(8):1384-1391. doi:10.1016/j.jada.2009.05.008
197. Michels N, Vynckier L, Moreno LA, et al. Mediation of psychosocial determinants in the relation between socio-economic status and adolescents' diet quality. *Eur J Nutr*. 2018;57(3):951-963. doi:10.1007/s00394-017-1380-8
198. Mieziene B, Emeljanovas A, Novak D, Kawachi I. Social Capital Promotes a Healthier Diet among Young Adults by Reducing Psychological Distress. *Nutrients*. 2022;14(23):5187. doi:10.3390/nu14235187
199. Mieziene B, Emeljanovas A, Novak D, Kawachi I. The Relationship between Social Capital within Its Different Contexts and Adherence to a Mediterranean Diet Among Lithuanian Adolescents. *Nutrients*. 2019;11(6):1332. doi:10.3390/nu11061332
200. Miller C, Braunack-Mayer A, Wakefield M, et al. "When we were young, it really was a treat; now sugar is just the norm every day" A qualitative study of parents' and young adults' perceptions and consumption of sugary drinks. *Health Promotion Journal of Australia*. 2020;31(1):47-57. doi:10.1002/hpja.257
201. Mirhadyan L, Moradi Latreyi S, Pasha A, Kazem Nejad Leili E. Junk Food Consumption and its Associated Factors in High School Students in Rasht in 2017. *Journal of Research Development in Nursing and Midwifery*. 2020;17(1):52-66. doi:10.29252/jgbfnm.17.1.52
202. Mirkarimi K, Bagheri D, Honarvar MR, Kabir MJ, Ozouni-Davaji RB, Eri M. Effective factors on fast food consumption among high-school students based on planned behavior theory. *Journal of Gorgan University of Medical Sciences*. 2017;18(4).
203. Mitri RN, Boulos C, Ziade F. Mediterranean diet adherence amongst adolescents in North Lebanon: the role of skipping meals, meals with the family, physical activity and physical well-being. *British Journal of Nutrition*. 2022;128(7):1349-1356. doi:10.1017/S0007114521002269
204. Monge-Rojas R, Colón-Ramos U, Chinnock A, Smith-Castro V, Reyes-Fernández B. Gender-based eating norms, the family environment and food intake among Costa Rican adolescents. *Public Health Nutr*. 2021;24(15):4840-4850. doi:10.1017/S1368980021000835
205. Moraes CH de C, Alvarenga M dos S, Moraes JMM, Cyrillo DC. Exploring Psychosocial Determinants of Eating Behavior: Fruit and Vegetable Intake Among Brazilian Adolescents. *Front Nutr*. 2021;8. doi:10.3389/fnut.2021.796894
206. Morton KL, Wilson AH, Perlmutter LS, Beauchamp MR. Family leadership styles and adolescent dietary and physical activity behaviors: a cross-sectional study. *International Journal of Behavioral Nutrition and Physical Activity*. 2012;9(1):48. doi:10.1186/1479-5868-9-48
207. Mwafi NR, Al-Rawashdeh IM, Al-Kubaisy WAQ, Ezzat WR, Al-Qazaqi RA, Salameh MH. Prevalence and factors related to fast food consumption among Mutah University Students, Jordan. *J Pak Med Assoc*. 2021;71(6):1-13. doi:10.47391/JPMA.274
208. Nagahata T, Nakade M, Hasegawa J, Kanehira N, Nishibori S. The Relationship between Skipping Breakfast and the Living Arrangements of University Students. *The Japanese Journal of Nutrition and Dietetics*. 2014;72(4):212-219. doi:10.5264/eiyogakuzashi.72.212
209. Nakade M, Shiozawa Y, Aiba N. Vegetable Intake at Breakfast and Associated Factors among Young Adults in Japan. *J Nutr Sci Vitaminol (Tokyo)*. 2020;66(Supplement):S406-S411. doi:10.3177/jnsv.66.S406
210. Nasirzadeh M, Abdolkarimi M, Asadollahi Z, Hashemian M. Investigating Factors Effect on Fruit and Vegetable Consumption: Applying Social Cognitive Theory. *J Community Health Res*. 2020;9(3):159-171. doi:10.18502/jchr.v9i3.4259
211. Navarro-Gonzalez I, Lopez-Nicolas R, Rodriguez-Tadeo A, Ros-Berruezo G, Martinez-Marin M, Domenech-Asensi G. Adherence to the Mediterranean diet by nursing students of Murcia (Spain). *Nutr Hosp*. 2014;30(1):165-172. doi:10.3305/nh.2014.30.1.7413
212. Nelson Laska M, Larson NI, Neumark-Sztainer D, Story M. Dietary patterns and home food availability during emerging adulthood: do they differ by living situation? *Public Health Nutr*. 2010;13(2):222-228. doi:10.1017/S1368980009990760
213. Neumark-Sztainer D, Wall M, Perry C, Story M. Correlates of fruit and vegetable intake among adolescents. *Prev Med (Baltim)*. 2003;37(3):198-208. doi:10.1016/S0091-7435(03)00114-2
214. Neumark-Sztainer D, Story M, Resnick MD, Blum RWm. Correlates of Inadequate Fruit and Vegetable Consumption among Adolescents. *Prev Med (Baltim)*. 1996;25(5):497-505. doi:10.1006/pmed.1996.0082

215. Neumark-Sztainer D, Hannan PJ, Story M, Croll J, Perry C. Family meal patterns: Associations with sociodemographic characteristics and improved dietary intake among adolescents. *J Am Diet Assoc*. 2003;103(3):317-322. doi:10.1053/jada.2003.50048
216. Nickelson J, Roseman MG, Forthofer MS. Associations between Parental Limits, School Vending Machine Purchases, and Soft Drink Consumption among Kentucky Middle School Students. *J Nutr Educ Behav*. 2010;42(2):115-122. doi:10.1016/j.jneb.2009.02.005
217. Niermann CYN, Kremers SPJ, Renner B, Woll A. Family Health Climate and Adolescents' Physical Activity and Healthy Eating: A Cross-Sectional Study with Mother-Father-Adolescent Triads. Buchowski M, ed. *PLoS One*. 2015;10(11):e0143599. doi:10.1371/journal.pone.0143599
218. O'Leary C, Cummins S, Smith RD, Cornelsen L. Like parent, like child: a cross-sectional study of intra-household consumption patterns of non-alcoholic beverages among British households with children. *Public Health Nutr*. 2022;25(7):1771-1779. doi:10.1017/S1368980021005061
219. Oliveira S S de, Bauermann G M, Alves M R, Toral N. Association of dietary intake with eating behavior, screen time, and physical activity among Brazilian adolescents. *Revista Chilena de Nutrición*. 2018;45(4):349-355. doi:10.4067/S0717-75182018000500349
220. Orlowski M, Shermadou S, Lee M, Hinson-Enslin A, Nahhas RW. Adolescent self-efficacy mediates the relationship between perceived parenting practices and fruit and vegetable consumption in the FLASHE study. *Health Educ J*. 2022;81(7):835-847. doi:10.1177/00178969221125637
221. Overcash F, Davey C, Zhang Y, Reicks M. Evening Meal Types and Family Meal Characteristics: Associations with Demographic Characteristics and Food Intake among Adolescents. *Nutrients*. 2020;12(4):886. doi:10.3390/nu12040886
222. Pandey S, Budhathoki M, Yadav DK. Psychosocial Determinants of Vegetable Intake Among Nepalese Young Adults: An Exploratory Survey. *Front Nutr*. 2021;8. doi:10.3389/fnut.2021.688059
223. Papadaki S, Mavrikaki E. Greek adolescents and the Mediterranean diet: factors affecting quality and adherence. *Nutrition*. 2015;31(2):345-349. doi:10.1016/j.nut.2014.09.003
224. Parikka S, Levälahti E, Martelin T, Laatikainen T. Single-parenthood and perceived income insufficiency as challenges for meal patterns in childhood. *Appetite*. 2018;127:10-20. doi:10.1016/j.appet.2018.04.005
225. Park S. Comparing the nutrient intake, quality of diet, eating habit scores and dietary behaviors of university students in Iksan, according to their type of residence. *Korean Journal of Community Nutrition*. 2003;8(6):876-888.
226. Parks CA, Blaser C, Smith TM, et al. Correlates of fruit and vegetable intake among parents and adolescents: findings from the Family Life, Activity, Sun, Health, and Eating (FLASHE) study. *Public Health Nutr*. 2018;21(11):2079-2087. doi:10.1017/S1368980018000770
227. Pearson N, Atkin AJ, Biddle SJ, Gorely T, Edwardson C. Parenting styles, family structure and adolescent dietary behaviour. *Public Health Nutr*. 2010;13(8):1245-1253. doi:10.1017/S1368980009992217
228. Pearson N, Ball K, Crawford D. Predictors of changes in adolescents' consumption of fruits, vegetables and energy-dense snacks. *British Journal of Nutrition*. 2011;105(5):795-803. doi:10.1017/S0007114510004290
229. Pedersen TP, Holstein BE, Damsgaard MT, Rasmussen M. Breakfast frequency among adolescents: associations with measures of family functioning. *Public Health Nutr*. 2016;19(9):1552-1564. doi:10.1017/S1368980016000112
230. Pelletier JE, Graham DJ, Laska MN. Social Norms and Dietary Behaviors among Young Adults. *Am J Health Behav*. 2014;38(1):144-152. doi:10.5993/AJHB.38.1.15
231. Peltzer K, Pengpid S. Correlates of healthy fruit and vegetable diet in students in low, middle and high income countries. *Int J Public Health*. 2015;60(1):79-90. doi:10.1007/s00038-014-0631-1
232. Pendergast FJ, Livingstone KM, Worsley A, McNaughton SA. Examining the correlates of meal skipping in Australian young adults. *Nutr J*. 2019;18(1):24. doi:10.1186/s12937-019-0451-5
233. Pengpid S, Peltzer K. Behavioral Risk Factors Of Non-Communicable Diseases Among A Nationally Representative Sample Of School-Going Adolescents In Indonesia. *Int J Gen Med*. 2019;12:387-394. doi:10.2147/IJGM.S226633
234. Pengpid S, Peltzer K. Prevalence and associated factors of skipping breakfast among university students from 28 countries: A cross-sectional study. *Int J Adolesc Med Health*. 2022;34(2):97-103. doi:10.1515/ijamh-2019-0256
235. Pengpid S, Peltzer K. Prevalence and correlates of fruit and vegetable consumption among adolescents in Laos. *Int J Adolesc Med Health*. 2021;33(6):555-560. doi:10.1515/ijamh-2019-0142

236. Perkins JM, Perkins HW, Craig DW. Misperceived norms and personal sugar-sweetened beverage consumption and fruit and vegetable intake among students in the United States. *Appetite*. 2018;129:82-93. doi:10.1016/j.appet.2018.06.012
237. Perkins JM, Perkins HW, Craig DW. Misperceptions of Peer Norms as a Risk Factor for Sugar-Sweetened Beverage Consumption among Secondary School Students. *J Am Diet Assoc*. 2010;110(12):1916-1921. doi:10.1016/j.jada.2010.09.008
238. Pokorski P, Nicewicz R, Jeżewska-Zychowicz M. Diet Quality and Changes in Food Intake during the University Studies in Polish Female Young Adults: Linkages with Food Experiences from Childhood and Perceived Nutrition Concerns. *Nutrients*. 2022;14(16):3399. doi:10.3390/nu14163399
239. Prichard I, Hodder K, Hutchinson A, Wilson C. Predictors of mother–daughter resemblance in dietary intake The role of eating styles, mothers' consumption, and closeness. *Appetite*. 2012;58(1):271-276. doi:10.1016/j.appet.2011.10.012
240. Qiu N, Moore JB, Wang Y, Fu J, Ding K, Li R. Perceived Parental Attitudes Are Indirectly Associated with Consumption of Junk Foods and Sugar-Sweetened Beverages among Chinese Adolescents through Home Food Environment and Autonomous Motivation: A Path Analysis. *Nutrients*. 2021;13(10):3403. doi:10.3390/nu13103403
241. Rafiroiu AC, Anderson EP, Sargent RG, Evans A. Dietary Practices of South Carolina Adolescents and Their Parents. *Am J Health Behav*. 2002;26(3):200-212. doi:10.5993/AJHB.26.3.5
242. Rezaeipour A, Youssefi F, Mahmoudi M, Shakeri M. Relationship between adolescents' nutritional and physical activity behaviors with their perceptions about parents' lifestyle behaviors. *Journal of Hayat*. 2007;13(3).
243. Riebl SK, MacDougal C, Hill C, et al. Beverage Choices of Adolescents and Their Parents Using the Theory of Planned Behavior: A Mixed Methods Analysis. *J Acad Nutr Diet*. 2016;116(2):226-239.e1. doi:10.1016/j.jand.2015.10.019
244. Riediger ND, Shooshtari S, Moghadasian MH. The Influence of Sociodemographic Factors on Patterns of Fruit and Vegetable Consumption in Canadian Adolescents. *J Am Diet Assoc*. 2007;107(9):1511-1518. doi:10.1016/j.jada.2007.06.015
245. Riggsbee K, Riggsbee J, Vilaro M, et al. More than Fast Food: Development of a Story Map to Compare Adolescent Perceptions and Observations of Their Food Environments and Related Food Behaviors. *Int J Environ Res Public Health*. 2018;16(1):76. doi:10.3390/ijerph16010076
246. Rimal RN. Intergenerational Transmission of Health: The Role of Intrapersonal, Interpersonal, and Communicative Factors. *Health Education & Behavior*. 2003;30(1):10-28. doi:10.1177/1090198102239256
247. Robinson E, Otten R, Hermans RCJ. Descriptive peer norms, self-control and dietary behaviour in young adults. *Psychol Health*. 2016;31(1):9-20. doi:10.1080/08870446.2015.1067705
248. Roesler A, Rojas N, Falbe J. Sugar-Sweetened Beverage Consumption, Perceptions, and Disparities in Children and Adolescents. *J Nutr Educ Behav*. 2021;53(7):553-563. doi:10.1016/j.jneb.2021.04.004
249. Rosenrauch S, Ball K, Lamb KE. Associations between perceived friends' support of healthy eating and meal skipping in adolescence. *Public Health Nutr*. 2017;20(18):3266-3274. doi:10.1017/S136898001700235X
250. Rossow I, Rise J. Concordance of parental and adolescent health behaviors. *Soc Sci Med*. 1994;38(9):1299-1305. doi:10.1016/0277-9536(94)90193-7
251. Salvy SJ, Miles JN V., Shih RA, Tucker JS, D'Amico EJ. Neighborhood, Family and Peer-Level Predictors of Obesity-Related Health Behaviors Among Young Adolescents. *J Pediatr Psychol*. 2016;42(2):jsw035. doi:10.1093/jpepsy/jsw035
252. San Román Mata S, Zurita Ortega F, Martínez Martínez A, Padial Ruz R, Chacón Cuberos R, Linares Manrique M. Adherence to Mediterranean Diet in university students from southern Spain depending on social and academic factors and religious. *Revista Española de Nutrición Humana y Dietética*. 2018;22(2):141-148. doi:10.14306/renhyd.22.2.446
253. Santaliestra-Pasías AM, Felez AP, Huybrechts I, et al. Social Environment and Food and Beverage Intake in European Adolescents: The HELENA Study. *Journal of the American Nutrition Association*. 2022;41(5):468-480. doi:10.1080/07315724.2021.1917462
254. Santomauro F, Lorini C, Tanini T, et al. Adherence to Mediterranean diet in a sample of Tuscan adolescents. *Nutrition*. 2014;30(11-12):1379-1383. doi:10.1016/j.nut.2014.04.008
255. Sato Y, Miyanaga M, Wang DH. Psychosocial Determinants of Fruit and Vegetable Intake in Japanese Adolescents: A School-Based Study in Japan. *Int J Environ Res Public Health*. 2020;17(15):5550. doi:10.3390/ijerph17155550

256. Schnettler B, Lobos G, Miranda-Zapata E, Denegri M, Ares G, Hueche C. Diet Quality and Satisfaction with Life, Family Life, and Food-Related Life across Families: A Cross-Sectional Pilot Study with Mother-Father-Adolescent Triads. *Int J Environ Res Public Health*. 2017;14(11):1313. doi:10.3390/ijerph14111313
257. Schnettler B, Grunert KG, Lobos G, Miranda-Zapata E, Denegri M, Hueche C. Maternal Food-Related Practices, Quality of Diet, and Well-Being: Profiles of Chilean Mother-Adolescent Dyads. *J Nutr Educ Behav*. 2018;50(8):776-787. doi:10.1016/j.jneb.2018.03.003
258. Schnettler B, Miranda-Zapata E, Orellana L, et al. Parents' Modeling During the COVID-19 Pandemic: Influences on Family Members' Diet Quality and Satisfaction With-Food-Related Life in Dual-Earner Parents With Adolescent Children. *Front Nutr*. 2022;9. doi:10.3389/fnut.2022.902103
259. Scully M, Morley B, Niven P, Crawford D, Pratt IS, Wakefield M. Factors associated with frequent consumption of fast food among Australian secondary school students. *Public Health Nutr*. 2020;23(8):1340-1349. doi:10.1017/S1368980019004208
260. Seedat R, Pillay K. Breakfast consumption and its relationship to sociodemographic and lifestyle factors of undergraduate students in the School of Health Sciences at the University of KwaZulu-Natal. *South African Journal of Clinical Nutrition*. 2020;33(3):79-85. doi:10.1080/16070658.2018.1564470
261. Sexton-Dhamu MJ, Livingstone KM, Pendergast FJ, Worsley A, McNaughton SA. Individual, social–environmental and physical–environmental correlates of diet quality in young adults aged 18–30 years. *Appetite*. 2021;162:105175. doi:10.1016/j.appet.2021.105175
262. Shahanjarini AK, Shojaezadeh D, Majdzadeh R, Rashidian A, Omidvar N. Application of an integrative approach to identify determinants of junk food consumption among female adolescents. *Iranian Journal of Nutrition Sciences & Food Technology*. 2009;4(2):61-70.
263. Sharma B, Harker M, Harker D, Reinhard K. Living independently and the impact on young adult eating behaviour in Germany. *British Food Journal*. 2009;111(5):436-451. doi:10.1108/00070700910957285
264. Silva PAS da, Froelich M, Rodrigues PRM, et al. Skipping breakfast associated with socioeconomic and lifestyle factors in Brazilian adolescents. *Cien Saude Colet*. 2022;27(10):4051-4062. doi:10.1590/1413-812320222710.04702022
265. Silva JB, Elias BC, Warkentin S, Mais LA, Konstantyner T. Factors associated with the consumption of ultra-processed food by Brazilian adolescents: National Survey of School Health, 2015. *Revista Paulista de Pediatria*. 2022;40:e2020362. doi:10.1590/1984-0462/2022/40/2020362
266. Simões AM, Machado CO, Höfelmann DA. Association of regular consumption of breakfast and health-related behavior among adolescents. *Cien Saude Colet*. 2021;26(6):2243-2251. doi:10.1590/1413-81232021266.15042019
267. Small ML, Morgan N, Bailey-Davis L, Maggs JL. The Protective Effects of Parent-College Student Communication on Dietary and Physical Activity Behaviors. *Journal of Adolescent Health*. 2013;53(2):300-302. doi:10.1016/j.jadohealth.2013.03.010
268. Smith TM, Pinard CA, Byker Shanks C, Wethington H, Blanck HM, Yaroch AL. Fruits and vegetables as a healthier snack throughout the day among families with older children: Findings from a survey of parent–child dyads. *Eat Behav*. 2015;17:136-139. doi:10.1016/j.eatbeh.2015.01.006
269. Stephens LDA, McNaughton SA, Crawford D, MacFarlane A, Ball K. Correlates of dietary resilience among socioeconomically disadvantaged adolescents. *Eur J Clin Nutr*. 2011;65(11):1219-1232. doi:10.1038/ejcn.2011.107
270. Stephens LD, McNaughton SA, Crawford D, Ball K. Longitudinal predictors of frequent vegetable and fruit consumption among socio-economically disadvantaged Australian adolescents. *Appetite*. 2014;78:165-171. doi:10.1016/j.appet.2014.03.023
271. Stephens LD, McNaughton SA, Crawford D, Ball K. Predictors of high-energy foods and beverages: a longitudinal study among socio-economically disadvantaged adolescents. *Public Health Nutr*. 2014;17(02):324-337. doi:10.1017/S136898001200482X
272. Stewart SD, Menning CL. Family Structure, Nonresident Father Involvement, and Adolescent Eating Patterns. *Journal of Adolescent Health*. 2009;45(2):193-201. doi:10.1016/j.jadohealth.2009.01.005
273. Stok FM, de Vet E, de Wit JB, Luszczynska A, Safron M, de Ridder DT. The proof is in the eating: subjective peer norms are associated with adolescents' eating behaviour. *Public Health Nutr*. 2015;18(6):1044-1051. doi:10.1017/S1368980014001268
274. Sugiyama S, Okuda M, Sasaki S, Kunitsugu I, Hobara T. Breakfast habits among adolescents and their association with daily energy and fish, vegetable, and fruit intake: a community-based cross-sectional study. *Environ Health Prev Med*. 2012;17(5):408-414. doi:10.1007/s12199-012-0270-1

275. Tabak I, Jodkowska M, Oblacińska A, Mikiel-Kostyra K. Can family meals protect adolescents from obesity? *Med Wieku Rozwoj*. 2012;16(4):313-321. http://www.ncbi.nlm.nih.gov/pubmed/23378411
276. Tak NI, Te Velde SJ, Oenema A, et al. The association between home environmental variables and soft drink consumption among adolescents. Exploration of mediation by individual cognitions and habit strength. *Appetite*. 2011;56(2):503-510. doi:10.1016/j.appet.2011.01.013
277. Tang JS, Haslam RL, Ashton LM, Fenton S, Collins CE. Gender differences in social desirability and approval biases, and associations with diet quality in young adults. *Appetite*. 2022;175:106035. doi:10.1016/j.appet.2022.106035
278. Tassitano RM, Martins CM de L, Cabral PC, Mota J, Tenório MCM, Silva GAP da. Psychosocial factors and physical activity as predictors of fruit and vegetable intake in college students. *Revista de Nutrição*. 2016;29(2):173-183. doi:10.1590/1678-98652016000200003
279. Thomson JL, Hennessy E, Landry AS, Goodman MH. Patterns of food parenting practices regarding junk food and sugary drinks among parent-child dyads. *Nutr J*. 2020;19(1):91. doi:10.1186/s12937-020-00610-3
280. Toumpakari Z, Haase AM, Johnson L. Adolescents' non-core food intake: a description of what, where and with whom adolescents consume non-core foods. *Public Health Nutr*. 2016;19(9):1645-1653. doi:10.1017/S1368980016000124
281. Vågstrand K, Linné Y, Karlsson J, Elfhag K, Karin Lindroos A. Correlates of soft drink and fruit juice consumption among Swedish adolescents. *British Journal of Nutrition*. 2009;101(10):1541. doi:10.1017/S0007114508083542
282. Vågstrand K. Sex differences among Swedish adolescents in mother–child relationships in the intake of different food groups. *British Journal of Nutrition*. 2010;103(8):1205-1211. doi:10.1017/S0007114509992972
283. van den Bogerd N, Maas J, Seidell JC, Dijkstra SC. Fruit and vegetable intakes, associated characteristics and perceptions of current and future availability in Dutch university students. *Public Health Nutr*. 2019;22(11):1951-1959. doi:10.1017/S136898001800174X
284. van den Broek N, Larsen JK, Verhagen M, Eisinga R, Burk WJ, Vink JM. The longitudinal link between mothers' and adolescents' snacking: The moderating role of television viewing. *Appetite*. 2018;120:565-570. doi:10.1016/j.appet.2017.10.010
285. van der Horst K, Kremers S, Ferreira I, Singh A, Oenema A, Brug J. Perceived parenting style and practices and the consumption of sugar-sweetened beverages by adolescents. *Health Educ Res*. 2006;22(2):295-304. doi:10.1093/her/cyl080
286. van der Horst K, Timperio A, Crawford D, Roberts R, Brug J, Oenema A. The School Food Environment: Associations with Adolescent Soft Drink and Snack Consumption. *Am J Prev Med*. 2008;35(3):217-223. doi:10.1016/j.amepre.2008.05.022
287. Van Hulst A, Jayanetti S, Sanson-Rosas AM, et al. Adolescents' reports of chaos within the family home environment: Investigating associations with lifestyle behaviours and obesity. Xie L, ed. *PLoS One*. 2023;18(1):e0280737. doi:10.1371/journal.pone.0280737
288. Van Lippevelde W, Vervoort L, Vangeel J, Goossens L. Can Parenting Practices Moderate the Relationship between Reward Sensitivity and Adolescents' Consumption of Snacks and Sugar-Sweetened Beverages? *Nutrients*. 2020;12(1):178. doi:10.3390/nu12010178
289. Vanhelst J, Béghin L, Drumez E, et al. Adolescents' diet quality in relation to their relatives' and peers' diet engagement and encouragement: the Healthy Lifestyle in Europe by Nutrition in Adolescence (HELENA) study. *Public Health Nutr*. 2018;21(17):3192-3201. doi:10.1017/S1368980018001787
290. Vaterlaus JM, Patten E V., Roche C, Young JA. #Gettinghealthy: The perceived influence of social media on young adult health behaviors. *Comput Human Behav*. 2015;45:151-157. doi:10.1016/j.chb.2014.12.013
291. Vejrup K, Lien N, Klepp KI, Bere E. Consumption of vegetables at dinner in a cohort of Norwegian adolescents. *Appetite*. 2008;51(1):90-96. doi:10.1016/j.appet.2007.12.004
292. Verstraeten R, Leroy JL, Pieniak Z, et al. Individual and Environmental Factors Influencing Adolescents' Dietary Behavior in Low- and Middle-Income Settings. Zeeb H, ed. *PLoS One*. 2016;11(7):e0157744. doi:10.1371/journal.pone.0157744
293. Verzeletti C, Maes L, Santinello M, Baldassari D, Vereecken CA. Food-related family lifestyle associated with fruit and vegetable consumption among young adolescents in Belgium Flanders and the Veneto Region of Italy. *Appetite*. 2010;54(2):394-397. doi:10.1016/j.appet.2009.12.010

294. Verzeletti C, Maes L, Santinello M, Vereecken CA. Soft drink consumption in adolescence: associations with food-related lifestyles and family rules in Belgium Flanders and the Veneto Region of Italy. *The European Journal of Public Health*. 2010;20(3):312-317. doi:10.1093/eurpub/ckp150
295. Videon TM, Manning CK. Influences on adolescent eating patterns: the importance of family meals. *Journal of Adolescent Health*. 2003;32(5):365-373. doi:10.1016/S1054-139X(02)00711-5
296. Vogel C, Shaw S, Strömmer S, et al. Inequalities in energy drink consumption among UK adolescents: a mixed-methods study. *Public Health Nutr*. 2023;26(3):575-585. doi:10.1017/S1368980022002592
297. Walton K, Horton NJ, Rifas-Shiman SL, et al. Exploring the Role of Family Functioning in the Association Between Frequency of Family Dinners and Dietary Intake Among Adolescents and Young Adults. *JAMA Netw Open*. 2018;1(7):e185217. doi:10.1001/jamanetworkopen.2018.5217
298. Wang DH, Kogashiwa M, Mori N, et al. Psychosocial Determinants of Fruit and Vegetable Consumption in a Japanese Population. *Int J Environ Res Public Health*. 2016;13(8):786. doi:10.3390/ijerph13080786
299. Watts AW, Valente M, Tu A, Mâsse LC. Eating Away from Home: Influences on the Dietary Quality of Adolescents with Overweight or Obesity. *Canadian Journal of Dietetic Practice and Research*. 2017;78(4):166-171. doi:10.3148/cjdpr-2017-010
300. Watts AW, Miller J, Larson NI, Eisenberg ME, Story MT, Neumark-Sztainer D. Multicontextual correlates of adolescent sugar-sweetened beverage intake. *Eat Behav*. 2018;30:42-48. doi:10.1016/j.eatbeh.2018.04.003
301. Watts AW, Loth K, Berge JM, Larson N, Neumark-Sztainer D. No Time for Family Meals? Parenting Practices Associated with Adolescent Fruit and Vegetable Intake When Family Meals Are Not an Option. *J Acad Nutr Diet*. 2017;117(5):707-714. doi:10.1016/j.jand.2016.10.026
302. Watts AW, Mâsse LC, Barr SI, Lovato CY, Hanning RM. Parent–Child Associations in Selected Food Group and Nutrient Intakes among Overweight and Obese Adolescents. *J Acad Nutr Diet*. 2014;114(10):1580-1586. doi:10.1016/j.jand.2014.04.018
303. Welsh EM, French SA, Wall M. Examining the Relationship Between Family Meal Frequency and Individual Dietary Intake: Does Family Cohesion Play a Role? *J Nutr Educ Behav*. 2011;43(4):229-235. doi:10.1016/j.jneb.2010.03.009
304. Wills WJ. Food and Eating Practices During the Transition from Secondary School to New Social Contexts. *J Youth Stud*. 2005;8(1):97-110. doi:10.1080/13676260500063728
305. Winpenny EM, Winkler MR, Stochl J, van Sluijs EMF, Larson N, Neumark-Sztainer D. Associations of early adulthood life transitions with changes in fast food intake: a latent trajectory analysis. *International Journal of Behavioral Nutrition and Physical Activity*. 2020;17(1):130. doi:10.1186/s12966-020-01024-4
306. Winpenny EM, van Sluijs EMF, White M, Klepp KI, Wold B, Lien N. Changes in diet through adolescence and early adulthood: longitudinal trajectories and association with key life transitions. *International Journal of Behavioral Nutrition and Physical Activity*. 2018;15(1):86. doi:10.1186/s12966-018-0719-8
307. Woodruff SJ, Hanning RM, McGoldrick K, Brown KS. Healthy eating index-C is positively associated with family dinner frequency among students in grades 6–8 from Southern Ontario, Canada. *Eur J Clin Nutr*. 2010;64(5):454-460. doi:10.1038/ejcn.2010.14
308. Woodward D, Boon J, Cumming F, Ball P, Williams H, Hornsby H. Adolescents' Reported Usage of Selected Foods in Relation to Their Perceptions and Social Norms for Those Foods. *Appetite*. 1996;27(2):109-117. doi:10.1006/appe.1996.0039
309. Wouters EJ, Larsen JK, Kremers SP, Dagnelie PC, Geenen R. Peer influence on snacking behavior in adolescence. *Appetite*. 2010;55(1):11-17. doi:10.1016/j.appet.2010.03.002
310. Wrobleski MM, Parker EA, Hager E, et al. Friends and Family: How African-American Adolescents' Perceptions of Dietary Beliefs and Behaviors of Others Relate to Diet Quality. *J Acad Nutr Diet*. 2018;118(12):2302-2310. doi:10.1016/j.jand.2018.07.021
311. Wu T, Stoots JM, Florence JE, Floyd MR, Snider JB, Ward RD. Eating Habits among Adolescents in Rural Southern Appalachia. *Journal of Adolescent Health*. 2007;40(6):577-580. doi:10.1016/j.jadohealth.2006.12.018
312. Xiang C, Lian C. The Prevalence of Unhealthy Snacking Behaviour and its Association with Individual and Environmental Factors Among College Students in Kuching, Sarawak. *Malaysian Journal of Public Health Medicine*. 2021;21(2):275-284. doi:10.37268/mjphm/vol.21/no.2/art.1001
313. Xu L, Odum M, Rogers CR, Wu Q, Wilmouth L. Association Between Family/Neighborhood Cancer Risk Factors and Adolescent Dietary Behaviors: A Parent-Adolescent Dyadic Analysis. *Am J Lifestyle Med*. 2022;16(5):622-632. doi:10.1177/1559827620949215

314. Yasugi H, Nishiyama M, Ohishi K. Lifestyle and breakfast-skipping among Japanese co-medical university students. *Dokkyo Journal of Medical Sciences*. 2008;35(2):101-107.
315. Yazdi Feyzabadi V, Keshavarz Mohammadi N, Omidvar N, Karimi-Shahanjarini A, Nedjat S, Rashidian A. Factors Associated With Unhealthy Snacks Consumption Among Adolescents in Iran's Schools. *Int J Health Policy Manag*. 2017;6(9):519-528. doi:10.15171/ijhpm.2017.09
316. Young EM, Fors SW, Hayes DM. Associations between Perceived Parent Behaviors and Middle School Student Fruit and Vegetable Consumption. *J Nutr Educ Behav*. 2004;36(1):2-12. doi:10.1016/S1499-4046(06)60122-X
317. Young EM, Fors SW. Factors Related to the Eating Habits of Students in Grades 9-12. *Journal of School Health*. 2001;71(10):483-488. doi:10.1111/j.1746-1561.2001.tb07285.x
318. Yuan C, Lv J, VanderWeele TJ. An Assessment of Health Behavior Peer Effects in Peking University Dormitories: A Randomized Cluster-Assignment Design for Interference. Kazembe L, ed. *PLoS One*. 2013;8(9):e75009. doi:10.1371/journal.pone.0075009
319. Yuhas M, Porter KJ, Hedrick V, Zoellner JM. Using a Socioecological Approach to Identify Factors Associated with Adolescent Sugar-Sweetened Beverage Intake. *J Acad Nutr Diet*. 2020;120(9):1557-1567. doi:10.1016/j.jand.2020.01.019
320. Zaborskis A, Grincaitė M, Kavaliauskienė A, Tesler R. Family structure and affluence in adolescent eating behaviour: a cross-national study in forty-one countries. *Public Health Nutr*. 2021;24(9):2521-2532. doi:10.1017/S1368980020003584
321. Zaborskis A, Lagunaite R, Busha R, Lubiene J. Trend in eating habits among Lithuanian school-aged children in context of social inequality: three cross-sectional surveys 2002, 2006 and 2010. *BMC Public Health*. 2012;12(1):52. doi:10.1186/1471-2458-12-52
322. Zahra J, Ford T, Jodrell D. Cross-sectional survey of daily junk food consumption, irregular eating, mental and physical health and parenting style of British secondary school children. *Child Care Health Dev*. 2014;40(4):481-491. doi:10.1111/cch.12068
323. Zarychta K, Mullan B, Luszczynska A. It doesn't matter what they say, it matters how they behave: Parental influences and changes in body mass among overweight and obese adolescents. *Appetite*. 2016;96:47-55. doi:10.1016/j.appet.2015.08.040
324. Zeinivanmoghadam L, Jalilian M, Mirzaei A. Predictors of Fruits and Vegetable Consumption in Adolescent Girls Based on Social Cognitive Theory. *Journal of Education and Community Health*. 2020;7(4):285-291. doi:10.29252/jech.7.4.285
325. Zhang R, Yang Q, Tang Q, Xi Y, Lin Q, Yang L. Is Adolescents' Free Sugar Intake Associated with the Free Sugar Intake of Their Parents? *Nutrients*. 2022;14(22):4741. doi:10.3390/nu14224741
326. Ziaei R, Shahi H, Dastgiri S, Mohammadi R, Viitasara E. Fruit and vegetable intake and its correlates among high-school adolescents in Iran: a cross-sectional study. *J Public Health (Bangkok)*. 2020;28(6):711-718. doi:10.1007/s10389-019-01084-2
327. Zolghadr R, Shojaeizadeh D, Sadeghi R, Majlesi F, Yekaninejad MS, Nejadsadeghi E. Effective factors of the healthy nutritional behavior based on the application of social cognitive theory among 13-15-year-old students. *Int J Pediatr*. 2019;7(3):9115-9123. doi:10.22038/ijp.2017.26462.2269
328. Zuercher JL, Wagstaff DA, Kranz S. Associations of food group and nutrient intake, diet quality, and meal sizes between adults and children in the same household: a cross-sectional analysis of U.S. households. *Nutr J*. 2011;10(1):131. doi:10.1186/1475-2891-10-131
329. Zytnick D, Park S, Onufrak SJ. Child and Caregiver Attitudes about Sports Drinks and Weekly Sports Drink Intake among U.S. Youth. *American Journal of Health Promotion*. 2016;30(3):e110-e119. doi:10.4278/ajhp.140103-QUAN-8
